# Extreme events and gender-based violence: a mixed-methods systematic review

**DOI:** 10.1016/S2542-5196(22)00088-2

**Published:** 2022-06-13

**Authors:** Kim Robin van Daalen, Sarah Savić Kallesøe, Fiona Davey, Sara Dada, Laura Jung, Lucy Singh, Rita Issa, Christina Alma Emilian, Isla Kuhn, Ines Keygnaert, Maria Nilsson

**Affiliations:** a Cardiovascular Epidemiology Unit, University of Cambridge, Cambridge, UK; b Department of Public Health and Primary Care, School of Clinical Medicine, University of Cambridge, Cambridge, UK; c Health Equity Network, University of Cambridge, Cambridge, UK; d Centre for Infectious Disease Genomics and One Health, Faculty of Health Sciences, Simon Fraser University, Burnaby, BC, Canada; e UCD Centre for Interdisciplinary Research, Education, and Innovation in Health Systems, School of Nursing, Midwifery, and Health Systems, University College Dublin, Dublin, Ireland; f Medical Faculty, Leipzig University, Leipzig, Germany; g London School of Hygiene & Tropical Medicine, London, UK; h Institute for Global Health, University College London, London, UK; i Medical Library, School of Clinical Medicine, University of Cambridge, Cambridge, UK; j International Centre for Reproductive Health, Department of Public Health and Primary Care, Ghent University, Ghent, Belgium; k Department of Epidemiology and Global Health, Umeå University, Umeå, Sweden

## Abstract

The intensity and frequency of extreme weather and climate events are expected to increase due to anthropogenic climate change. This systematic review explores extreme events and their effect on gender-based violence (GBV) experienced by women, girls, and sexual and gender minorities. We searched ten databases until February, 2022. Grey literature was searched using the websites of key organisations working on GBV and Google. Quantitative studies were described narratively, whereas qualitative studies underwent thematic analysis. We identified 26 381 manuscripts. 41 studies were included exploring several types of extreme events (ie, storms, floods, droughts, heatwaves, and wildfires) and GBV (eg, sexual violence and harassment, physical violence, witch killing, early or forced marriage, and emotional violence). Studies were predominantly cross-sectional. Although most qualitative studies were of reasonable quality, most quantitative studies were of poor quality. Only one study included sexual and gender minorities. Most studies showed an increase in one or several GBV forms during or after extreme events, often related to economic instability, food insecurity, mental stress, disrupted infrastructure, increased exposure to men, tradition, and exacerbated gender inequality. These findings could have important implications for sexual-transformative and gender-transformative interventions, policies, and implementation. High-quality evidence from large, ethnographically diverse cohorts is essential to explore the effects and driving factors of GBV during and after extreme events.

## Introduction

As a result of climate change, the intensity, frequency, duration, timing, and spatial extent of extreme weather and climate events are changing.^1^ Between 2000 and 2019, floods, droughts, and storms alone have affected nearly 4 billion people worldwide, costing over 300 000 lives.^2^ The occurances of these extreme events represents a drastic change since the period 1980–99, with the frequency of floods increasing by 134%, storms by 40%, and droughts by 29%.^2^ Without implementation of appropriate adaptation and mitigation measures, these tolls are expected to rise further as climate change progresses.

Extreme weather and climate events negatively affect human lives, ecosystems, and economies. These effects are diverse and mediated both through the environment (eg, floods increasing the risk of infectious diseases and wildfire smoke resulting in respiratory symptoms) and social systems (eg, disruption of essential services, violence, and resource loss).^3^, ^4^ However, such visible implications often overshadow more veiled consequences, including gender-based violence (GBV) experienced by women, girls, and sexual and gender minorities.^5^

Increased GBV has been observed in both natural and human-caused crises and disasters, due to socioeconomic instability, structural power inequalities, health-care inaccessibility, resource scarcity, breakdowns in safety and law enforcement, and increases in (perceived) stress.^6^ Compounding effects on gender equity, the short-term and long-term consequences of GBV also have myriad global public health implications including physical injury, unwanted pregnancy, exposure to HIV or other sexually transmitted infections, fertility problems, internalised stigma, mental health conditions (eg, depression, anxiety, suicidal ideation, and post-traumatic stress disorder), and ramifications for children (including those born out of rape).^7^ However, full understanding of the effect of extreme weather and climate events on GBV is limited. Failing to address GBV in the context of extreme events might undermine efforts towards gender equality and sustainable interventions.^8^, ^9^, ^10^

Previous systematic reviews and reports have revealed the effects of natural disasters on violence against women,^11^, ^12^ as well as the association between GBV and other disaster settings and emergencies,^13^, ^14^ including COVID-19.^15^, ^16^ This systematic review adds an assessessment of the effect of extreme weather and climate events on GBV experienced by women, girls, and sexual and gender minorities (an often overlooked group in existing literature despite possible unique GBV risks associated with frequent marginalisation). This Review intends to inform future research, planning, intervention, and policy efforts to reduce the burden and incidence of GBV as well as its associated harms in the context of predicted increases in the rates and intensity of extreme weather and climate events.

**Key messages**

• This mixed-methods systematic review examines how gender-based violence (GBV) experienced by women, girls, and sexual and gender minorities could be affected by extreme weather and climate events
• Although often overlooked in the literature, sexual and gender minorities might have unique GBV risks due to their frequent marginalisation
• The findings suggest a potential increase of GBV during or after extreme events related to factors such as economic instability, food insecurity, mental stress, loss of control, disrupted infrastructure (including health and judicial services), increased exposure to men, culture or tradition, and exacerbated gender inequities within patriarchal societies
• Our Review highlights the need for high-quality evidence from large, ethnographically diverse cohorts to explore the effects and underlying driving factors of GBV during and after extreme events
• Interventions should consider contextually relevant factors such as local norms, traditions, and social attitudes related to gender roles

## Methods

This Review protocol was prospectively registered with PROSPERO (CRD-42021237271). The findings were reported following PRISMA guidelines (appendix pp 1–3).^17^, ^18^ Key definitions of GBV and extreme events applied throughout this Review can be found in the panel.

Panel **Key definitions**

**Gender based violence (GBV)**

GBV is defined as violence directed towards a person because of their gender or violence that affects persons of particular genders disproportionately due to structural and societal power imbalances. This violence includes interpersonal or intimate partner violence, domestic violence, physical violence, emotional violence, sexual violence (eg, rape, attempt to rape, and sexual harassment), technological violence (eg, online stalking and cyberbullying), early (ie, <18 years old) or forced marriage, human trafficking, and witch killings.^5^, ^19^ We specifically focus on women, girls, and sexual and gender minorities. Sexual and gender minorities (SGMs) are defined as individuals whose sexuality, biological sex, gender identity, or gender expression are different than the majority norms in a given society. SGMs include, but are not limited to, people that identify as queer, lesbian, gay, bisexual, asexual, gender non-conforming, intersex, transgender, and Two-Spirited.^20^

**Extreme events**

The Intergovernmental Panel on Climate Change defines extreme events as extreme weather or climate variables that are substantially different from average or usual weather or climate patterns and have adverse effects on human health or livelihood and related events (eg, heatwaves, storms, floods, droughts, and wildfires).^21^, ^22^ Although the 2021 Intergovernmental Panel on Climate Change report^22^ does not dismiss a potential link between geophysical natural disasters and human-induced climate change, the purpose of this Review is to examine how extreme events known to be related to human-induced climate change affect GBV. As such, geophysical natural disasters such as earthquakes, tsunamis, and volcanoes have not been included. The included extreme weather and climate events are collectively referred to as extreme events.

### Search strategy and selection criteria

Ten electronic databses were searched with English search terms: PubMed, Embase via Ovid, MEDLINE via Ovid, CINAHL via EBSCOhost, PsycINFO via EBSCOhost, Global Health via EBSCOhost, Scopus, Web of Science Core Collection, SciELO via Web of Science, and LiLACS. No restrictions on the language or date of included studies was applied. Databases were searched from inception through to Feb 26, 2021, with an update to Feb 2, 2022. Using a combination of free-text terms and medical subject headings, we used vocabulary related to “women”, “girls”, “sexual and gender minority”, “violence”, “extreme weather”, and “extreme climate” informed by previous systematic reviews.^6^, ^17^ The full search strategy for each database is included in the appendix (pp 4–6). To ensure comprehensive information synthesis,^23^ grey literature and information was searched using Google and 17 websites of relevant organisations working on GBV or climate change (appendix p 7). Forward and backward screening of all records included in the full-texts and relevant publications (eg, reviews, commentaries, and grey literature reports) was used to find any additional records fitting the inclusion criteria. We included studies that reported on extreme events and GBV towards women, girls, and sexual and gender minorities without restriction on age-group, and primary peer-reviewed quantitative, qualitative, or mixed-method studies, or grey literature containing primary data (eg, non-governmental organisation or government reports).

### Study selection

After de-duplication, titles and abstracts were double-screened following the selection criteria by eight researchers using Rayyan. Studies meeting the inclusion criteria were double-screened in full text. Conflicts emerging in both stages were resolved among authors by consensus. We excluded (1) non-human studies, (2) studies on violence against cisgender heterosexual men and boys, (3) studies on extreme events or natural or human disasters without an established relationship to anthropogenic climate change (eg, earthquakes, volcanoes, tsunamis), (4) conference proceedings, studies that lacked access to the full text, and single case reports or news articles, and (5) peer-reviewed studies with secondary study designs (eg, reviews), and grey literature without primary data. We included non-published theses if they showed up in our search strategy, but did not search thesis repositories. All non-English records were translated or reviewed by a native or fluent speaker of the research team, which included members fluent in several languages, including Arabic, Dutch, English, French, German, Spanish, and Swedish.

### Data extraction and study quality assessment

Data from included studies were independently extracted in duplicate by six researchers. Discrepancies were adjudicated by consensus. Extracted information included: author, year, study title, study design, study population, participant demographics, extreme event type, exposure ascertainment, recruitment procedure, number of participants, GBV type, outcomes ascertainment, percentage or number of individuals reporting outcome (ie, GBV), association measures with summary estimate, and 95% CIs. An open field to record additional relevant information was also available.

Quality assessment was performed in duplicate using the Newcastle Ottawa scale for quantitative studies and the Critical Appraisal Skills Programme tool for qualitative studies.^24^, ^25^ Newcastle Ottawa scale summary scores were converted into Healthcare Research and Quality scores (categorised as good, fair, or poor).^25^ No summary scores were employed for Critical Appraisal Skills Programme scores following previous studies using this tool and Cochrane's recommendation to avoid scoring.^26^, ^27^ Mixed-methods studies were assessed with both the Newcastle Ottawa scale and the Critical Appraisal Skills Programme. Grey literature was appraised using the Authority, Accuracy, Coverage, Objectivity, Date, Significance checklist.^28^

### Data analysis and synthesis

Due to heterogeneity in study design, exposures, and outcomes of included studies, meta-analyses were not possible. Hence, quantitative data were narratively synthesised. When available, we summarised the direction of effect.

Quantitative and qualitative studies were grouped and synthesised by the type of extreme event reported (eg, storms and drought). Studies were further grouped on the basis of the same extreme event (eg, Hurricane Katrina and Black Friday Bushfires) or the same region or country. Qualitative data underwent thematic analysis. Two authors independently used inductive analysis to generate and agree on a codebook, which was then applied to all qualitative studies.

### Patient involvement

Due to the nature of the study, no patients were involved in conceptualising or conducting the study.

## Results

**Figure 1:**
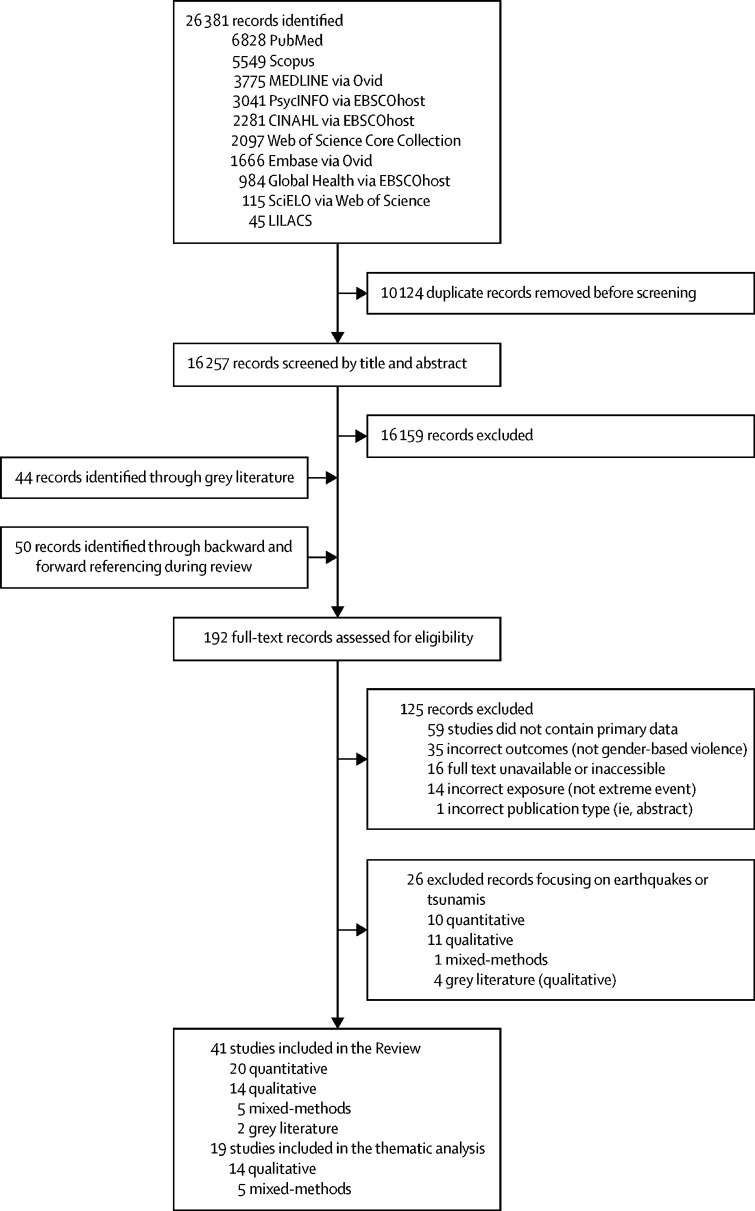
Study selection process

**Figure 2:**
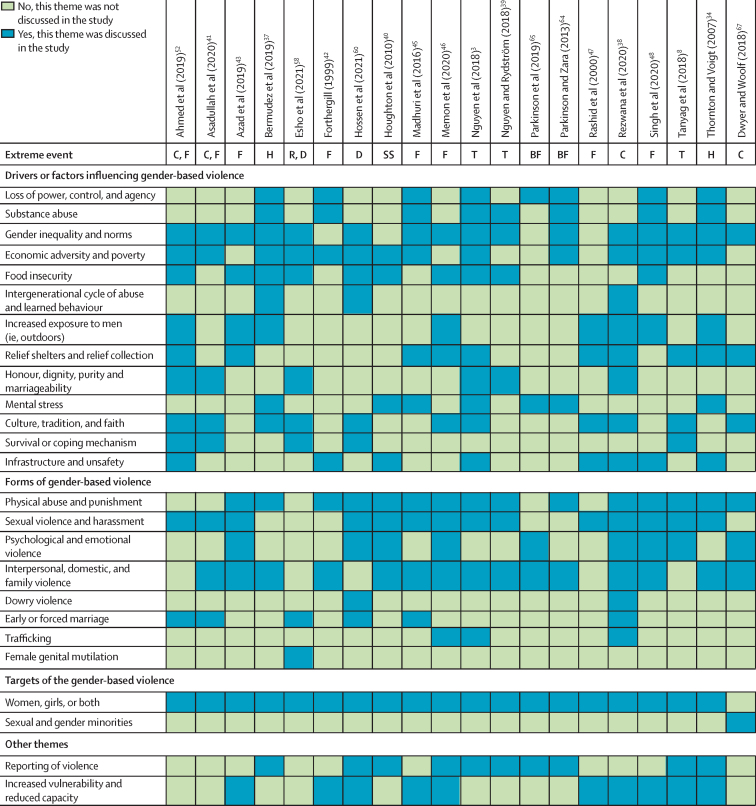
Thematic analysis C=Cyclones. F=Floods. H=Hurricane. R=Rainfall. D=Drought. SS=Snowstorm. T=Typhoon. BF=Bushfire.

The database search strategy yielded 26 381 publications. After de-duplication, 16 257 records were screened by title and abstract and 192 as full-text (figure 1). 41 records (ie, 39 peer-reviewed articles, two grey literature records) were included, with summary characteristics reported in the table by extreme event type and results in the appendix (pp 8–17). The different themes reported are grouped into three domains: type of GBV experienced, driving or influencing factors of GBV, and targets of GBV (figure 2). We outline the articles excluded in full-text screening and reasons for exclusion (n=125; appendix pp 18–26). Although not the focus of this Review, we provide an overview with summary characteristics of the excluded studies exploring the effects of earthquakes, tsunamis, or unspecified natural disasters on GBV (n=26; appendix pp 27–32).^68^, ^69^, ^70^, ^71^, ^72^, ^73^, ^74^, ^75^, ^76^, ^77^, ^78^, ^79^, ^80^, ^81^, ^82^, ^83^, ^84^, ^85^, ^86^, ^87^, ^88^, ^89^, ^90^, ^91^, ^92^, ^93^

**Table:** Summary characteristics of the included studies and reports (n=41)

	**Study design**	**Study period**	**Country**	**Extreme event**	**Type or definition of gender-based violence**	**Population source**	**Perpetrator**	**Participants (n)**
**Peer-reviewed**								
Anastario et al (2009)^29^	Quantitative, cross-sectional study and a verbally administered randomised questionnaire	2006 and 2007	Mississippi, USA	Hurricane Katrina (2005)	Physical and sexual gender-based violence, classified as intimate partner violence or sexual violence	People who were internally displaced due to Hurricane Katrina and living in Federal Emergency Management Agency travel trailer parks since the 2005, Gulf Coast hurricane season*;* mean age 42·7 years	Partner or ex-partner; no specified perpetrator	106 women in 2006 and 314 women in 2007
Fagen et al (2011)^30^	Quantitative, cross-sectional study measuring point prevalence twice	Spring 2005 (first cross-section) and spring 2006, (second cross-section)	USA	Hurricane Katrina (2005)	Sexual violence, defined as being taken advantage of sexually, forced sexual touching, unwanted sexual intercourse, experiencing sexual assault or sexual harassment, and other known correlates of sexual violence	Undergraduate students at the University of New Orleans in the spring semester before Hurricane Katrina (2005) and the spring semester following Hurricane Katrina (2006) that were part of the CORE Alcohol and Drug Survey; age not reported	No specific perpetrators reported	237 female students in 2005 and 215 female students in 2006
Harville et al (2011)^31^	Quantitative, cross-sectional study using the Conflicts Tactics Scales-2	March, 2006, and May, 2007	USA	Hurricane Katrina (2005)	Intimate partner violence; four included scales measuring negotiation behaviours (eg, suggesting a compromise); psychologically aggressive behaviours (eg, shouting and yelling); physical assault (eg, punching and kicking); and sexual coercion (eg, insisting on sex and sex without a condom)	Post-partum women affected by Hurricane Katrina who were admitted to Tulane Lakeside Hospital, Metairie, LA, or Women's Hospital, Baton Rouge, LA, for childbirth between March, 2006, and May, 2007; age >18 years	Intimate partner or ex-partner	123 post-partum women completed the Conflicts Tactics Scales-2
Picardo et al (2010)^32^	Quantitative, cross-sectional study	Not reported	Louisiana, USA	Hurricane Katrina (2005)	Physical (eg, being hit or threatened verbally) and sexual (eg, forced to have sex) abuse	English-speaking women of a reproductive age displaced by Hurricane Katrina residing in Louisiana Federal Emergency Management Agency housing; aged 19–49 years	Spouse, partner, or another person	66 women
Schumacher et al (2010)^33^	Quantitative, cross-sectional study measuring point prevalence twice	Data collected Feb 24–July 31, 2007 (6 months before and after Hurricane Katrina)	USA	Hurricane Katrina (2005)	Interpersonal violence referring broadly to physical aggression and threats of physical aggression as well as a wide range of psychologically abusive or controlling behaviours perpetrated against a current or former intimate partner	Married or cohabitating adults living in 23 southernmost counties of Mississippi at the time of Hurricane Katrina; age >18 years	Intimate partner or ex-partner	251 women, 194 men
Thornton and Voigt (2007)^34^	Qualitative, using content analysis of more than 2500 newspaper articles	2005	USA	Hurricane Katrina (2005)	Sexual assault (not further defined)	Articles were on crime during and following Hurricane Katrina (including mass media reports, daily journals of law enforcement, and victim advocate narratives)*;* age not applicable	Not specified	Not applicable (as content analysis of newspaper articles)
Temple et al (2011)^35^	Quantitative, cross-sectional study	March, 2009	USA	Hurricane Ike (2008)	Teen dating violence, defined as sexual or physical violence towards a dating partner (ie, both perpetration and victimisation)	Primarily low-income high-school students who returned to Galveston island post-storm and attended Galveston's only public high school; age >14 years	Dating partner	584 girls, 464 boys
Westhoff et al (2008)^36^	Quantitative, cross-sectional study using a semi-structured interview	Not reported	Belize	Hurricane Mitch (1998)	Gender-based violence, defined as sexual violence (eg, forced to have sex against their will and trading sex for food, protection, or other survival necessities) and domestic violence (ie, did their partner or husband hit them)	Refugees and internally displaced people from banana farm workstations or health clinics in Southern Belize were included; mean age 28·8 (SD 10·6) years	Husband, friend, family member (eg, father), or other internally displaced person	202 refugees and internally displaced people
Bermudez et al (2019)^37^	Qualitative, using transcripts obtained with a photo-elicitation approach over the course of three sessions per person (in-depth interviews)	October and November, 2017	Southwestern Haiti	Hurricane Matthew (2016)	Violence against women and violence against children (not further defined)	Data from the Transforming Households: Reducing Incidence of Violence in Emergencies project taking place in Côteaux in the Sud department in southwestern Haiti; ages 13–17 years and 25–66 years	Not clearly reported	34 comprising of eight adult women, ten adult men, eight adolescent women, and eight adolescent men
Rezwana et al (2020)^38^	Qualitative case study with grounded theory approach using in-depth interviews, ethnographic observation, and group discussions	3-month study period in 2016 (following cyclone Roanu)	Bangladesh	Cyclone Roanu (2016)	Gender-based violence, defined as any act that results in, or is likely to result in, physical, sexual, or mental harm or suffering to women; including threats of such coercion or arbitrary deprivation of liberty, whether occurring in public or private life	Women survivors of gender-based violence and eight men (including two perpetrators of gender-based violence) in the Barguna region (ie, coastal Bangladesh) hit by the cyclone in 2016; age range of the women 17–50 years	Spouses, family members, strangers, and acquaintances	29 women survivors of gender-based violence and eight men for context
Nguyen (2018)^3^	Qualitative, using in-depth interviews	April, 2014, to May, 2015	Philippines	Typhoon Haiyan (also known as Super Typhoon Yolanda) 2013	Violence against women and girls, including domestic violence, intimate partner violence, sexual violence, and incest	Sexually abused women and girls, representatives of the community-based and non-governmental organisations, members of international non-governmental organisations and government officials—mostly from the provinces most affected by Typhoon Haiyan; age not reported	Family members, acquaintances husbands, partners, strangers, uniformed officials	Total 42 including 21 survivors of sexual assault (9 women and 12 girls)
Nguyen and Rydström (2018)^39^	Qualitative, using in-depth interviews and focus group discussions	2015	Philippines and Vietnam	Typhoon Haiyan (also known as Super Typhoon Yolanda) 2013, and Typhoon Nari (also known as Storm Number 11) 2013	Intimate partner violence (eg, beating women)	From the Philippines, women who had been subjected to intimate partner violence, activists and scholars of the local universities, and representatives of civil society organisations and governmental agencies, age not reported; from Vietnam, long Lanh men and women and local officials, ages 20–65 years	Partner or ex-partner	42 from the Philippines and 147 from Vietnam
Tanyag (2018)^8^	Qualitative, using in-depth interviews	January to April, 2015, and April to May, 2016	Philippines	Typhoon Haiyan (also as known as Super Typhoon Yolanda) 2013	Sexual and gender-based violence (not further defined)	Informants on the effect of internal displacement post-disaster on women and girls, representing five government agencies, a women's political party, national commission on human rights, nine international humanitarian and development non-governmental organisations, three international organisations, and seven local non-governmental organisations; age not reported	Not reported	26 (19 women and 7 men)
Houghton et al (2010)^40^	Mixed methods: qualitative, in-depth interviews and quantitative, cross-sectional	August, 2008	New Zealand	Snowstorm (2006)	Domestic violence, defined as a broad range of controlling behaviours, commonly of a physical, sexual, or psychological nature that typically involves fear, intimidation, and emotional deprivation	Qualitative interviews with agencies and individuals post-event; data including descriptive statistics from the Women's Refuge database and case file summaries; age not reported	Not specifically reported—partners mentioned	Seven qualitative interviews; quantitative not reported
Asadullah et al (2020)^41^	Mixed methods: qualitative, individual open-ended interviews and focus group discussions with women victims; quantitative, cross-sectional survey using structured household interviews	Qualitative, May and June, 2016, and quantitative, the 2014 Women's Life Choices and Attitudes Survey	Bangladesh: qualitative, eight villages in four southern-western coastal districts and quantitative, four south-western coastal districts and 60 districts	Cyclone and storm surge and flood	Child marriage, not explicitly defined but mentioned as an arranged marriage before age 18 years	The qualitative data were obtained from four coastal districts of Bangladesh; these districts are mostly vulnerable to salinity intrusion, cyclone and storm surge and tidal waters; age range 17–45 years; the quantitative data were from the 2014, Women's Life Choices and Attitudes Survey; age not reported	Family members	In-depth interviews with 75 women married before age 18 years and eight focus group discussions with three women and two men respondents; quantitative data from 353 women exposed to extreme weather and 5919 controls
Forthergill (1999)^42^	Qualitative, in-depth interviews	1997–98	USA	North Dakota flood (1997)	Domestic violence; women battering (not further defined)	Women living in Grand Forks (ND, USA) and East Grand Forks (MN, USA) were sampled in the research; the study covered two case studies from the sampled participants; age not reported for the overall sample; the two cases were in their early forties	Intimate partner or ex-partner	40 women (of which 20 were interviewed twice); the study details interviews with two women reporting domestic violence
Azad et al (2013)^43^	Mixed methods: qualitative, interviews, observation (both participatory and non-participatory), and focus group discussions; quantitative, cross-sectional using field survey tools	Done in 2011 assessing the experiences of women affected by floods in the past 5 years	Northern Bangladesh	Floods	Domestic violence and sexual violence, general violence, and harassment against women; harassment was defined as violence of mental, physical, or sexual dimensions (eg, mental torture, verbal abuse, physical abuse, beatings from the husband, and sexual violence); note, the study was not specifically assessing gender-based violence	The study area is Sirajganj District, which is prone to severe floods. On the basis of the severity of floods over the past 5 years, the present study included women from four sub-districts (known as upazilasii); age not reported	Not specifically reported; some women mentioned their husband	Quantitative, 185 semi-structured individual interviews were conducted among women affected by floods; qualitative, five focus group discussions with eight to 12 participants and five key informant interviews (eg, with non-governmental organisations)
Frasier et al (2004)^44^	Quantitative, cross-sectional survey with 83 questions addressing health and experience with the flood	September, 1999	USA	Floods following Hurricane Floyd (1999)	Intimate partner violence, physical intimate partner violence, eg, pushed, slapped, kicked, or otherwise physically hurt; verbal intimate partner violence, yelled at, put down, yelled at in public, or made to feel bad about themselves; threaten intimate partner violence; threaten to physically hurt	Women employed at work sites identified through the North Carolina Manufacturing Directory meeting the following criteria: (1) blue-collar workplaces with >50 permanent employees, (2) at least 50% women, (3) no wellness programme, (4) no participation in a previous Health Works for Women project, and (5) no immediate plans for plant closure; age >18 years	Intimate partner or ex-partner	785 women were included in the baseline study
Madhuri (2016)^45^	Qualitative, using focus group discussions and in-depth interviews	Not reported	India	Floods	Eve-teasing (ie, public sexual harassment) and verbal, physical, and sexual harassment and domestic violence	Women and girl survivors in flood-affected areas of the Purnia and Katihar districts of Bihar of different ages, castes, and income levels; age range not reported, but included young girls (8 years) and older women (64 years)	Not reported	About 150 focus group discussions of eight–ten women; ten discussions for in-depth interviews of women heads of household (widows or those deserted by a husband)
Memon (2020)^46^	Mixed methods: qualitative, in-depth interviews; quantitative, cross-sectional study	Not reported	Pakistan	Floods	Emotional violence (ie, verbal, mental, or emotional abuse due to a stressful scenario or gender-specific task and cultural barriers); physical violence (ie, physical abuse that women experience during and after disaster scenarios when women bear physical abuse because of low social status and stress due to loss of income); sexual violence (ie, sexual nature of harassment in the form of inappropriate touch and an increased risk of assaults and harassment due to no-to-little privacy or, in some extreme cases, human trafficking and rape)	Women who had been living in settlement camps where temporary flood-relief shelters were made near Larkana and Khairpur for at least 2 years; age not reported	Intimate partner or a stranger	20 women (ten from Larkana and ten from Khaipur)
Rashid and Michaud (2000)^47^	Qualitative case study using in-depth interviews and informal discussions	Not reported (post-1998 floods)	Bangladesh	Floods	Sexual and mental harassment (not further defined)	Rural areas of Manikganj, and urban areas of Kamrangichor and Badda in Dhaka with nine girls; aged 15–19 years	Strangers	Nine girls
Singh (2010)^48^	Qualitative, using in-depth interviews	Not reported	India	Floods	Family conflict and violence (not further defined)	15 women from a total of 15 households were selected, ten from Umela Phata and five from Kolivada; age 12–68 years	Not reported	15 women, each representing one household
Allen et al (2021)^49^	Quantitative, ecological study	2008 and 2014	Kenya	Severe weather events defined as flood >10 days (2006–14)	Intimate partner violence, including physical and sexual violence and emotional abuse	Demographic and Health Surveys for the health and wellbeing of women and young children in low-income and middle-income countries; the women were of childbearing age and had been married and lived with a man; aged 15–49 years	Partner, husband, or other family members	2008, 4903 women and 2014, 4512 women
Díaz and Saldarriaga (2020)^50^	Quantitative, ecological study	2005–14	Peru	Rainfall shocks (ie, drought and floods)	Physical intimate partner violence (eg, slapped, had something thrown at her, pushed, shoved, hair pulled, hit with a fist or something else, choked, burned, threatened with a weapon); sexual interpersonal violence or intimate partner violence (eg, forced to have sexual intercourse or sexual act); emotional or psychological interpersonal violence or intimate partner violence (eg, humiliate her, threatened to hurt or harm her or someone she cared about, or threaten to take away the children)	Participants of the Peruvian Demographic and Health Surveys who lived in rural areas of the Peruvian Andes; focused on women who are the female household head, married or cohabit with a partner, and who live in the municipality for at least 1 year; aged 15–49 years	Partner or ex-partner	15 110 women (including 640 exposed to a flood event and 421 exposed to a drought event)
Miguel (2005)^51^	Quantitative, cross-sectional study combined with longitudinal data on rainfall and murder	Surveys from 2001–02; longitudinal rainfall and murder data from 1992–2002; rainfall surveys from 1996–2001	Tanzania	Extreme rainfall (ie, drought or flood; 1996–2001)	Witch killings and murders of older women	Data came from the Village Council Survey and the Household survey; the Village Council Survey was administered in 71 villages and relied both on interviews with village council members and local administrative records; the household survey was administered to households from each village randomly sampled from the Village Tax Register; mean age of murder victims was 57·6 years	Witch killers	67 villages; five–15 village officials interviewed; rainfall data from the station in the district capital; 1293 households (ie, 15–20 per village)
Ahmed et al (2019)^52^	Mixed methods: quantitative, cross-sectional using a questionnaire; qualitative, open-ended in-depth interviews	June to September, 2015	Bangladesh (Sunamganj and Brahmanbaria)	Flash flooding, cyclones, and floods related to cyclones	Early female marriage (ie, child marriage) and sexual violence, defined as marriage of girls <18 years; sexual violence, referring to rape, sexual abuse, unwanted touching, being coerced, threatened, or otherwise forced to watch private sexual acts	Participating households were recruited from two geographic units (ie, villages) that were highly vulnerable to flooding (Alipur) and cyclones (Chandi); aged 15–49 years	Not fully specified, among others spouse	120 household heads across the two villages; 42 households from Alipur and 78 households from Chandi
Cools et al (2020)^53^	Quantitative, cross-sectional study with repeated cross-sectional surveys for nine out of 17 included countries.	2003–13	17 unspecified countries in sub-Saharan Africa	Rainfall shocks, including drought (2003–13)	Intimate partner violence, including pushing, shaking, slapping, throwing something at her, or twisting an arm, striking with a fist or something that could cause injury, kicking or dragging, attempting to strangle or burn, threatening with a knife, gun, or another type of weapon, and attacking with a knife, gun, or another type of weapon, physically forcing intercourse or any other sexual acts, or forcing her to perform sexual acts with threats or in any other way	Women living in 17 unspecified countries across sub-Saharan Africa who answered the Demographic and Health Surveys, aged 15–49 years	Most recent partner or husband	The complete cross-sectional sample used in the first part of the analysis has 149 032 women; in the second part of the analysis, the sample is limited to nine countries with repeated surveys; the third part of the analysis has 50 512 women who experienced violence from their most recent partner in 1060 grid cells
Epstein et al (2020)^54^	Quantitative, cross-sectional study	2011–18	19 sub-Saharan African countries (including Sierra Leone, Togo, Benin, Côte d'Ivoire, Cameroon, Gabon, Chad, Democratic Republic of the Congo, Rwanda, Burundi, Uganda, Kenya, Tanzania, Malawi, Mozambique, Zimbabwe, Zambia, Namibia, and Angola)	Extreme rainfall and drought (2011–18)	Intimate partner violence, including experience of a controlling partner and experiencing emotional violence, physical violence, or sexual violence in the 12 months before the survey	Data on intimate partner violence, household, and community characteristics were ascertained from the Demographic and Health Surveys from partnered women aged 15–49 years	Intimate partner or husband	83 990 partnered women of whom 9019 experienced severe drought and 19 639 experienced mild or moderate drought
Cooper et al (2021)^55^ a re-analysis of Epstein et al (2020)^54^	Quantitative, ecological study	2000–18	Sub-Saharan Africa, Latin America and the Caribbean, and Asia	Extreme rainfall and droughts (2000–18)	Intimate partner violence, including physical violence, sexual violence, emotional violence, and controlling behaviours	Demographic Health Surveys data from sub-Saharan Africa, Latin America and the Caribbean, and Asia; aged 15–49 years	Partner or husband	363 428 women from 40 countries
Rai et al (2020)^56^	Quantitative, cross-sectional study using the National Family Survey-4 combined with data from the Emergency Events Database and the Ministry of Agriculture and Farmer Welfare	January, 2015, to December, 2016 (National Family Survey-4), merged with drought data from January, 2015, to December, 2016, and Emergency Events Database from 2013–14	India	Drought (2015–16) and cyclones (2013–14)	Intimate partner violence, including emotional violence (whether the respondent was insulted, humiliated, or threatened by the husband or partner), physical violence (whether the respondent was pushed, slapped, punched, kicked, strangled, had hair pulled, or was threatened with a knife by the husband or partner), sexual violence (whether the respondent was ever forced into unwanted sex or physically forced to perform sexual acts by the husband or partner)	A nationally representative sample of ever-married women aged 15–49 years living across ten drought-affected states and the four states that were exposed to two cyclones in 2013 and 2014 is part of the National Family Survey-4	Intimate partner or husband	31 045 affected by drought and 8469 affected by cyclones, 39 514 total
Corno et al (2020)^57^	Quantitative, ecological study using a simple equilibrium model of the marriage market	Sub-Saharan Africa 1994 and 2013; India 1998	Sub-Saharan Africa and India	Extreme rainfall and drought (referred to as weather shocks)	Early or child marriage (ie, <18 years of age)	Sub-Saharan Africa, women who were part of the Demographic and Health Surveys in 1994 and 2013, aged 25–49 years; India, ever-married women that were part of the Demographic and Health Surveys in 1998, aged 25–49 years	Family members	400 000 women
Esho et al (2021)^58^	Qualitative, using focus group discussions and key informant interviews	2020	Kenya	Extreme rainfall and drought	Early marriage (ie, <18 years of age) and female genital mutilation (ie, cutting off parts of the female external genitalia for non-medical reasons)	Key informants included government and community representatives such as county ministries for land, education, and health, administrative chiefs, community elders, parents; focus group discussion participants including young women who were beneficiaries of the 2017 Kajiado Technical Vocational and Education Training programme aged 18–25 years	Family members	12 key informants and eight women focus group discussion participants
Sekhri and Storeygard (2011)^59^	Quantitative, ecological study	2002–07	India	Dry shocks (ie, below average rainfall)	Crimes against women including dowry deaths, domestic violence, sexual harassment, murder, and kidnapping	Crime data from the National Crime Records Bureau (Ministry of Home Affairs), district-level demographic data from the 2001 Census of India, dowry data from the Gender, Marriage, and Kindship Survey; age not reported	Partner (or husband) or ex-partner, family members, or a stranger	Not applicable
Hossen et al (2021)^60^	Qualitative, using focus group discussions (ethnographic)	2018–20	Bangladesh	Drought	Early marriage (ie, <18 years of age), physical (eg, kicking, shaking, or pushing), emotional or psychological and sexual violence (eg, rape), and violence in the workplace	Women aged ≥40 years living in Badlagaree village and the Gaibandha district	Family member, husband, factory owner, or fellow worker	47 women
Carrico et al (2020)^61^	Quantitative, cross-sectional study	Retroactive data was collected in 2014 for the time of interest (from 1989 to 2013)	Bangladesh	Heat waves and dry spells (the authors constructed two extreme weather variables: Warm Spell Duration Indicator and Consecutive Dry Days; 1989–2013)	Age at marriage, including early (ie, <18 years of age) and forced marriage among girls and women; conditions of marriage including acceptance of less desirable marriage proposals for daughters and irrespective of age	Administered the Bangladesh Environment and Migration Survey to 1695 randomly selected households in nine mouzas; aged 18–23 years	Family members	First marriages of women aged 11–23 years, 615 women household heads and marriage conditions, 505 women household heads
Sanz-Barbero et al (2018)^62^	Quantitative, ecological longitudinal time-series study	2008–16 summertime (ie, May 1 to September 30)	Madrid, Spain	Heatwave (daily maximum temperature above heatwave threshold of 34°C; 2008–16)	Intimate partner femicides, reports on intimate partner violence and 016 interpersonal violence or intimate partner violence telephone helpline calls (not further defined)	People calling the 016 helpline for interpersonal violence or intimate partner violence from the government delegation for gender violence, police reports on interpersonal violence or intimate partner violence and intimate partner femicides from the integral monitoring system for the case of gender violence of the Ministry of Interior; age not reported	Not reported but partner by gender-based violence definition used	Population of Madrid—study period is 1377 days in the summertime
Molyneaux et al (2020)^63^	Quantitative, cross-sectional study	April, 2012, to January, 2013	Australia	Black Saturday bushfires (2009)	Violence or assault against women with a specific focus on interpersonal violence (ie, violence directed against an intimate partner or ex-partner)	25 communities across ten Victorian rural and regional locations divided across bushfire-affectedness using the Victorian Government Rapid Impact Assessment process; age range 18·3–87·7 years	Intimate partner or ex-partner	967 included in the analysis, 585 (60%) women
Parkinson and Zara (2013)^64^	Qualitative, using in-depth interviews	Not reported	Australia	Black Saturday bushfires (2009)	Domestic violence (not further defined)	Women from Mitchell and Murrindindi were recruited through advertisements in newspapers and flyers placed in key community areas or through word of mouth; age not reported	Intimate partner or ex-partner	30 women and 47 workers
Parkinson (2019)^65^	Qualitative, using in-depth interviews	2009 to 2011	Australia	Black Saturday bushfires (2009)	Domestic or interpersonal violence (not further defined)	Women from the local government areas of Mitchell and Murrindindi in the Victoria region as they were the worst affected areas on Black Saturday with 159 of the total 173 deaths; aged 20s-60s years	Intimate partner or ex-partner	30 women
**Grey literature**								
International Federation of Red Cross and Red Crescent Societies^66^	Mixed methods: quantitative, using cross-sectional household surveys and qualitative, using focus group discussions and key informant interviews	May–November, 2017	Philippines, Indonesia, Laos	Philippines, Typhoon Haiyan (2013); Indonesia, the Western Nusa Tenggara floods (2017) and Aceh Earthquake (2016); Laos, Oudomxay floods (2016) and Typhoon Ketsana (2009)	Early marriage (ie, <18 years of age), domestic violence (not further defined)	Participants of household surveys and focus group discussions initiated by the International Federation of Red Cross and Red Crescent Societies; age not reported	Husbands, strangers, and community members	Total, 1778 survey participants, 358 focus group discussion participants, and 58 key informants; from the Philippines, 805 household surveys and 12 focus group discussions; from Indonesia, 709 household surveys and 16 focus group discussions; from Laos, 265 household surveys and four focus group discussions
Dwyer and Woolf (2018)^67^ Oxfam research report	Qualitative, participatory field research that consisted of individual story-sharing, community mapping, and Talanoa sessions with sexual and gender minorities	May, 2017	Fiji	Tropical cyclone Winston (2016)	Gendered violence against sexual and gender minorities (eg, domestic violence)	Sexual and gender minorities that participated in the *Down By the River Sessions*, which is part of Oxfam's Pacific humanitarian capacity-building project; age not reported	Family, community members, and strangers	30 stories from people of sexual and gender minorities

Studies were done in more than 40 countries, including: the USA (n=9),^29^, ^30^, ^31^, ^32^, ^33^, ^34^, ^35^, ^42^, ^44^ Bangladesh (n=7),^38^, ^41^, ^43^, ^47^, ^52^, ^61^, ^60^ India (n=5),^45^, ^48^, ^56^, ^57^, ^59^ the Philippines (n=4),^3^, ^8^, ^39^, ^66^ Australia (n=3),^63^, ^64^, ^65^ Kenya (n=2),^49^, ^58^ Belize,^36^ Fiji,^67^ Haiti,^37^ Indonesia,^66^ Laos,^66^ New Zealand,^40^ Pakistan,^46^ Peru,^50^ Tanzania,^51^ Vietnam,^39^ and Spain.^62^ Four studies included multiple (unspecified) countries in sub-Saharan Africa, Latin America and the Caribbean, and Asia.^57^, ^53^, ^54^, ^55^ 20 publications were quantitative,^29^, ^30^, ^31^, ^32^, ^33^, ^35^, ^36^, ^44^, ^49^, ^50^, ^51^, ^53^, ^54^, ^55^, ^56^, ^57^, ^59^, ^61^, ^62^, ^63^ 15 were qualitative,^3^, ^8^, ^34^, ^37^, ^38^, ^39^, ^42^, ^45^, ^47^, ^48^, ^58^, ^60^, ^64^, ^65^, ^67^ and six were mixed-methods.^40^, ^41^, ^43^, ^46^, ^52^, ^66^ All quantitative studies were either cross-sectional or ecological, whereas qualitative studies used in-depth interviews (n=15),^3^, ^8^, ^37^, ^38^, ^39^, ^40^, ^41^, ^42^, ^43^, ^44^, ^45^, ^46^, ^47^, ^48^, ^52^ focus groups (n=7),^38^, ^39^, ^41^, ^43^, ^45^, ^58^, ^60^ ethnographic observations (n=3),^38^, ^43^, ^60^ key informant interviews (n=2),^66^, ^58^ informal discussions,^47^ content analysis,^32^ and individual story sharing in Talanoa sessions (ie, a Pacific island form of inclusive, participatory, and transparent dialogue).^67^ All studies were published after 1999 with the majority published since 2015 (n=25).^3^, ^8^, ^37^, ^38^, ^39^, ^41^, ^45^, ^46^, ^48^, ^49^, ^50^, ^52^, ^53^, ^54^, ^55^, ^56^, ^57^, ^58^, ^60^, ^61^, ^62^, ^63^, ^65^, ^66^, ^67^ Studies were done from 1989^61^ to 2020,^58^ although six studies did not state the study period.^36^, ^45^, ^46^, ^47^, ^48^, ^64^ Participant populations ranged from nine girls aged 15–18 years^47^ to 363 428 women aged 15–49 years.^55^

Although most qualitative studies were of reasonable methodological quality, quantitative studies were of poor quality (appendix pp 33–37). The majority of quantitative studies relied on self-reporting for outcome ascertainment, making them prone to recall bias,^29^, ^30^, ^31^, ^32^, ^33^, ^35^, ^36^, ^41^, ^42^, ^43^, ^44^, ^46^, ^50^, ^51^, ^52^, ^53^, ^54^, ^55^, ^56^, ^57^, ^59^, ^61^, ^62^, ^63^ non-response bias,^29^, ^30^, ^32^, ^35^, ^36^, ^40^, ^41^, ^42^, ^43^, ^46^, ^51^, ^53^, ^54^, ^59^, ^61^, ^62^, ^63^ and selection bias.^30^, ^31^, ^40^, ^41^, ^43^ Interpreting the results of ecological studies is potentially susceptible to ecological fallacy. Of the quantitative studies, ten were considered of good,^31^, ^33^, ^49^, ^51^, ^53^, ^54^, ^55^, ^57^, ^61^ six of fair,^29^, ^32^, ^56^, ^59^, ^62^, ^63^ and nine of poor^30^, ^35^, ^36^, ^40^, ^41^, ^43^, ^44^, ^46^, ^52^ quality as assessed by Healthcare Research and Quality scores. Few studies justified sample size^49^, ^50^, ^51^, ^53^, ^54^, ^55^, ^56^, ^57^, ^63^ or established comparability between respondents and non-respondents.^31^, ^33^, ^44^, ^49^, ^50^, ^52^, ^55^, ^56^, ^57^ Almost all qualitative studies had clear research aim statements.^3^, ^8^, ^34^, ^37^, ^38^, ^39^, ^40^, ^41^, ^42^, ^43^, ^45^, ^46^, ^47^, ^48^, ^52^, ^58^, ^60^, ^64^, ^65^ Only five studies adequately considered researcher–participant relationships^38^, ^41^, ^46^, ^47^, ^48^ and six used appropriate recruitment strategies.^8^, ^38^, ^45^, ^46^, ^48^, ^65^ Considering the limited and heterogenous evidence on this topic, no articles were excluded on the basis of quality.

The extreme events studied include: floods (n=13),^41^, ^42^, ^43^, ^44^, ^45^, ^46^, ^47^, ^48^, ^49^, ^50^, ^51^, ^52^, ^57^ droughts (n=10),^50^, ^51^, ^53^, ^54^, ^55^, ^56^, ^57^, ^58^, ^59^, ^60^ hurricanes (n=9),^29^, ^30^, ^31^, ^32^, ^33^, ^34^, ^35^, ^36^, ^37^ cyclones (n=6),^38^, ^39^, ^41^, ^52^, ^66^, ^67^ extreme rainfall and rainfall shocks (n=6),^50^, ^51^, ^53^, ^55^, ^57^, ^58^ typhoons (n=4),^3^, ^8^, ^39^, ^66^ wildfires (n=3),^63^, ^64^, ^65^ heatwaves (n=2),^61^, ^62^ and a snowstorm.^40^ Six studies were on Hurricane Katrina.^29^, ^30^, ^31^, ^32^, ^33^, ^34^ Wildfires were only researched in Australia,^63^, ^64^, ^65^ whereas droughts were largely studied in sub-Saharan Africa.^51^, ^53^, ^54^, ^55^, ^56^ No study directly attributed the extreme event to anthropogenic climate change. Instead, studies referred to a high likelihood that the studied event would increase in frequency, intensity, or both with climate change. This lack of direct attribution is likely due to the difficulty in identifying the causes of extreme weather events and whether they are linked to climate change. Attribution studies can be complex and rely on a wide range of data to simulate the Earth's climate.^94^, ^95^, ^96^

Different GBV forms and definitions were included: intimate partner, domestic, or family violence (ie, violence inflicted by partners, ex-partners, or family members) (n=26);^3^, ^29^, ^31^, ^33^, ^36^, ^37^, ^38^, ^39^, ^40^, ^42^, ^43^, ^44^, ^45^, ^46^, ^49^, ^50^, ^53^, ^54^, ^55^, ^56^, ^62^, ^63^, ^64^, ^65^, ^66^, ^67^ sexual violence, harassment (eg, unwanted intercourse or rape, eve-teasing, and inappropriate touching), and transactional sex for survival (n=25);^3^, ^8^, ^29^, ^30^, ^31^, ^32^, ^33^, ^34^, ^36^, ^38^, ^40^, ^43^, ^45^, ^46^, ^47^, ^49^, ^50^, ^52^, ^53^, ^54^, ^55^, ^56^, ^59^, ^60^, ^63^ physical violence (eg, punching and kicking) (n=21);^29^, ^31^, ^32^, ^33^, ^35^, ^36^, ^37^, ^38^, ^39^, ^40^, ^42^, ^43^, ^44^, ^45^, ^49^, ^50^, ^53^, ^54^, ^55^, ^56^, ^60^ verbal, emotional, or psychological violence (n=16);^31^, ^33^, ^38^, ^40^, ^43^, ^44^, ^45^, ^46^, ^47^, ^48^, ^49^, ^50^, ^54^, ^55^, ^56^, ^60^ forced or early marriage (ie, for girls aged <18 years; n=9);^8^, ^38^, ^41^, ^52^, ^57^, ^58^, ^60^, ^61^, ^66^ femicides (n=2);^59^, ^62^ female genital mutilation (cutting parts of female genitalia for non-medical reasons);^58^ dowry deaths;^59^ witch killings;^51^ incest;^3^ and teen-dating violence.^35^ All peer-reviewed studies focused on (cisgender) women and girls. Only one grey literature record focusing on sexual and gender minorities was found.^67^ This publication showed that sexual and gender minorities were blamed for the cyclone the community experienced, described as “God's punishment for their sins”.^67^ Most studies assessed multiple GBV forms or applied a broad GBV definition. Perpetrators of violence included partners, ex-partners, or husbands (n=30),^3^, ^29^, ^31^, ^32^, ^33^, ^35^, ^36^, ^37^, ^38^, ^39^, ^40^, ^42^, ^43^, ^44^, ^45^, ^46^, ^48^, ^49^, ^50^, ^53^, ^54^, ^55^, ^56^, ^59^, ^60^, ^62^, ^63^, ^64^, ^65^, ^66^ family members (eg, fathers, uncles, or brother-in-laws; n=13),^3^, ^36^, ^37^, ^38^, ^41^, ^43^, ^49^, ^52^, ^57^, ^58^, ^59^, ^60^, ^61^ strangers (n=10),^36^, ^38^, ^43^, ^45^, ^46^, ^47^, ^51^, ^52^, ^59^, ^66^ community members (eg, neighbours or religious leaders; n=3),^43^, ^51^, ^57^ relief workers (eg, non-governmental organisation officers or government officials; n=2),^38^, ^43^ and fellow workers.^60^

Studies investigating hurricanes, cyclones, and typhoons present mixed evidence. Of the quantitative studies, seven showed an increase in^29^, ^31^, ^33^, ^52^, ^66^ or frequent reporting of^32^, ^36^different forms of GBV. Four of these were of fair or good quality.^29^, ^31^, ^32^, ^33^ By contrast, two poor-quality studies showed no significant difference in the prevalence of sexual violence^30^ or teen-dating violence.^35^ Although more speculative, the qualitative studies seemed to suggest that drivers of violence as well as violence itself increased during and after storms.^3^, ^8^, ^34^, ^37^, ^38^, ^39^, ^66^, ^67^

Although one study showed no significant differences in any sexual violence measures towards women before and after Hurricane Katrina (2005),^30^ five studies suggested that GBV, particularly interpersonal violence or intimate partner violence (IPV), increased following the hurricane.^29^, ^31^, ^32^, ^33^, ^34^ Schumacher and colleagues^33^ reported that physical victimisation increased from 4·2% to 8·3% for women (p=0·01). Likewise, a study on internally displaced people in Mississippi, USA found that sexual violence and IPV rates increased in the year following Hurricane Katrina, and did not return to baseline in the displacement phase—with crude rates of GBV increasing from 4·6 per 100 000 population per day to 16·3 per 100 000 population per day in 2006, and remaining elevated at 10·1 per 100 000 population per day in 2007.^29^ This observed increase was similar to that in a 2011 study indicating that adjusted relative risk with 95% CI of hurricane-caused damage and post-partum women being insulted, sworn at, shouted at, or yelled at was 1·23 (1·02–1·48); being slapped, pushed, or shoved was 5·28 (1·93–14·45); and being beaten up, punched, or kicked was 8·25 (1·68–40·47).^31^ Three other studies on Hurricane Ike,^35^ Hurricane Mitch,^36^ and Hurricane Matthew^37^ showed; (1) no evidence that teen-dating violence among adolescent girls differed between those who were and were not evacuated (adjusted odds ratio teen-dating violence victimisation 0·62 [95% CI 0·22–1·77]),^35^ (2) that sexual and domestic violence in internally displaced people was frequently reported following Hurricane Mitch,^36^ and (3) that multiple IPV drivers resulted from the hurricane-induced humanitarian context (eg, loss of power or control, inequitable gender norms, and daily stressors).^37^

Four studies explored cyclone impacts on GBV in Bangladesh and India.^38^, ^41^, ^52^, ^56^ Survey-adjusted models indicated that cyclone exposure was positively associated with emotional IPV (adjusted odds ratio 1·59 [95% CI 1·20–2·10]). Although not statistically significant, the cyclone was suggested to have had a positive association with physical and sexual IPV in India.^56^ Qualitative data from a case study on Cyclone Roanu in Bangladesh indicated direct and indirect associations to forced marriage and trafficking immediately before, during, and after the cyclones.^38^ Much of the reported GBV showed public dimensions such as communities turning a blind eye to harassment in shelters as reported by one survivor: *“*The shelter is not safe for us. Young men come from seven or eight villages. They eve tease [verbally harass] girls and young women. They try to touch or molest them. I feel frightened to stay in the shelters. I stay at my house rather than taking my teenage daughter to the shelters”.^38^ Two mixed-methods studies exploring areas prone to cyclones and flooding suggest a positive association between climate shocks and the early marriage of daughters.^41^, ^52^ This increase in early marriages was mentioned as a coping strategy to minimise damage-related expenses and prevent harm to the family's or daughter's reputation due to the unmarried daughter being subjected to sexual violence.^52^ One household head described: “The 2013 cyclone destroyed most of my belongings (house, cattle, furniture, and money) and I have no hope and don't know what to do … I am scared about what to do with my youngest unmarried daughter [under 18] since I cannot provide for her basic needs. If I can marry off all my daughters, then I can reduce my financial burden, and it will reduce my family size as well”.^52^ The only report on sexual and gender minorities found that this group were blamed for cyclone Winston in Fiji and experienced violence, harassment, isolation, stigma, and poor access to relief resources.^67^ Notably, although the cyclones seemed to increase GBV, GBV also augments gender inequity and poverty, further increasing the vulnerability of targeted women, girls, and sexual and gender minorities to extreme events.^38^

Lastly, three qualitative studies and a grey literature report explored the effect of the 2013 Super Typhoon Haiyan on GBV in the Philippines and Vietnam.^3^, ^8^, ^39^, ^66^ Nguyen^3^ argues that although Haiyan exacerbated women's and girls’ vulnerability, GBV is rooted in gender inequalities embedded in social structures, which become intensified as survival becomes the priority. These results were emphasised further in findings comparing data from the Philippines and Vietnam suggesting that increased governmental efforts to combat violence against women in Vietnam pre-typhoon might have had a pre-emptive effect in reducing post-typhoon violence.^39^ Tanyag^8^ further elaborates that violence is often caused by economic strains and that the prevalence of adolescent pregnancies functions as an indicator of routine GBV, such as rape, in camps for internally displaced people. The International Federation of Red Cross and Red Crescent Societies found an increase in trafficking for sexual exploitation and abuse in the Philippines and an increased risk of GBV during the first week to one month in Laos post-typhoon.^66^

Floods were predominantly examined in qualitative studies exploring women's vulnerability in their aftermath.^41^, ^42^, ^43^, ^44^, ^45^, ^46^, ^47^, ^48^, ^49^, ^50^, ^51^, ^52^, ^66^ Of the quantitative studies, three good-quality studies showed an increase in reported physical and sexual interpersonal or intimate partner violence^45^, ^49^ and in witch killings.^51^ Similarly, four poor-quality studies showed increases in early marriage^41^, ^52^ and the experience of harassment during and after the floods.^43^, ^46^ Two studies (of good and poor quality) reported no evidence suggesting that the prevalence of IPV was significantly different between women affected by floods and those not affected.^44^, ^50^ One grey literature record showed increased sexual harassment in shelters.^66^ The qualitative records suggested that floods might be associated with increased patterns of harassment and violence towards women and girls,^42^, ^43^, ^45^, ^47^, ^48^, ^52^, ^66^ whereas a link to early marriage was not directly connected.^41^

Similar to storms, a correlation is suggested to exist between flooding incidence and early marriage.^41^, ^52^ Spikes in early marriages were observed in Bangladesh coinciding with the 1998 and 2004 floods.^41^ Next to being viewed as a way to reduce family costs and safeguard marriageability and dignity, these marriages are often less expensive due to flood-induced impoverishment lowering expectations.^41^, ^52^ One girl (aged 13 years) elaborated: “My parents were in abject poverty and our house was frequently flooded. After my father's death, I went to Dhaka for work. One day, my maternal uncle called and lied that my mother was ill. He then insisted that I return to my home village. When I returned home, my mother forced me to marry for the sake of family honour and dignity”.^41^ In contrast, Corno and colleagues^57^ found that floods have no effect on child marriage in sub-Saharan Africa, and might even reduce child marriage hazard in India.

Further studies reported observed increases of other GBV forms (ie, emotional, physical, and sexual) during and after extreme events suggested to be related to a range of factors including patriarchal attitudes, societal norms, social dislocation, economic difficulties, and disaster reduction efforts.^42^, ^45^, ^48^, ^49^, ^66^ One study highlighted the emotional violence women experience following floods. Women are often considered to be primarily responsible for fulfilling family needs and carrying emotional burdens leading to intense psychological pressures. Notably, violence rates seem to increase when women are displaced post-floods.^46^ When taking refuge in shelters, camps, or community centres after evacuation, women in Bangladesh, Indonesia, India, and Pakistan were reportedly exposed to verbal, sexual harassment, and sexual, physical, or emotional violence committed by partners, ex-partners, and strangers.^43^, ^45^, ^47^, ^66^ Young girls left alone by their parents faced anxiety and shame as well as fears of harassment.^45^ Described by a 16 year old girl: “It was more scary during the floods because there were more mastaans [hoodlums] and goondahs [thugs] hanging around. Some unknown boys were roaming around in their noukas [boats] and harassed the girls around here”.^47^

In contrast to the majority of evidence, Frasier and colleagues^44^ indicate no significant increase in IPV incidence post-flood resulting from Hurricane Floyd among US blue-collar women living in southern rural communities. However, 32·6% of women did not respond to the IPV question.^44^ Díaz and Saldarriaga^50^ did not find increases in physical IPV following flood event exposure in Peru, although they did find increases following drought.

Ten studies explored the effect of drought on interpersonal or intimate partner violence, early marriage, female genital mutilation, dowry deaths, and witch killings.^50^, ^51^, ^53^, ^54^, ^55^, ^56^, ^57^, ^58^, ^59^, ^60^ Of the good and fair quality quantitative studies, five showed an increase in IPV (ie, sexual and physical),^50^, ^54^, ^59^ dowry violence and death,^59^ witch killings,^51^ and child marriage,^57^ as well as some evidence for increases in controlling behaviour.^55^ Three studies showed no evidence of increased violence.^53^, ^55^, ^56^ One study showed a decrease in early marriage in India.^57^ The two qualitative studies showed an increase in women's and girls’ vulnerabilities during drought including for early marriage and female genital mutilation.^60^, ^58^

The four studies located in sub-Saharan Africa found conflicting results.^53^, ^54^, ^55^, ^57^ On the basis of Demogrpahic and Health Surveys data from 83 990 women across 19 sub-Saharan African countries, Epstein and colleagues^54^ showed that women living in severe drought had higher risks of sexual violence (marginal risk difference 1·2 [95% CI 0·4–2·0], p=0·001), physical violence (0·8 [0·1–1·4], p=0·019), and a controlling partner (3·0 [1·3–4·6], p<0·001) than women who had no experience of drought. Yet, Cools and colleagues^53^ found no robust evidence that droughts increase IPV using the Demogrpahic and Health Surveys data of 149 032 women from 17 sub-Saharan African countries. To resolve this apparent conflict, Cooper and colleagues^55^ integrated the methods of these previous studies,^53^, ^54^ drew on a bigger sub-Saharan African dataset, and included data on Latin America and the Caribbean and Asia.^55^ They found little association between drought and most IPV forms—although there was some evidence that an increase in controlling behaviour could be observed across the continents.^55^ Lastly, short-term changes in economic conditions related to drought were shown to correspond to an increase (2·3–3·0%) in child marriage of girls (aged 12–17 years) in sub-Saharan Africa, but to a decline (4·2–4·3%) in child marriage in India.^57^ These differences were explained by differences in the direction of marriage payments (in sub-Saharan Africa, traditionally the groom's family pays the bride's family, *vs* in India the bride's family pays the groom's family).^57^ However, in Bangladesh where the bride's family pays a dowry, two studies showed increased incentives to marry off daughters in periods of drought.^41^, ^52^ These increased incentives are argued to be a result of the requirement of a low dowry payment when daughters marry young.^41^, ^52^, ^60^ Among the Masai community in Kenya, poverty and loss of agricultural production seemed to be associated with an increase in early marriage and female genital mutilation, as explained by a mother-in-law to a new bride, “in this community, uncut girls cannot be accepted”.^58^

Similarly, the two other studies in India showed varying results.^56^, ^59^ They did not find an increase of emotional^56^ or sexual violence,^56^, ^59^ but did find suggestive evidence for a positive association with physical violence,^56^, ^59^ domestic violence, dowry violence, and dowry deaths.^59^ Dowry violence and deaths (ie, harm inflicted on women before or after a marriage coinciding with dowry demands) could give households access to a large dowry payment, which can increase income during economic distress.^59^ Women with higher education, from wealthier households, and with husbands that had no history of alcohol consumption were less likely to experience any form of IPV than women who had lower education, were from poorer households, and had husbands with a history of alcohol consumption.^56^

Lastly, extreme rainfall in Tanzania, resulting in either floods or drought, seemed to be associated with an increase in the murder of so-called witches, but not in other murders, compared with years of average rainfall. The study indicated these witch killings are likely driven by economic conditions (ie, income shocks) because they largely occurred in poor, rural areas of Tanzania dependent on agriculture. Alternative theories such as the scapegoat theory suggest households can eliminate the perceived cause of their suffering by murdering a witch able to control disasters.^51^

The two studies (of good and fair quality) on heatwaves suggest an increase in GBV.^61^, ^62^ During the year of and following the heatwaves, there was a documented increased risk of women and girls marrying (ie, early or forced; odds ratio 1·167 [standard error 0·077], p=0·020), which was strongest among those aged 18–23 years. Families also seemed to accept less desirable marriage proposals, with women marrying into poorer households and to less educated husbands than usually accepted.^61^ Furthermore, the risk of intimate partner femicides (relative risk 1·40 [95% CI 1·00–1·97]) increased three days after heatwaves, IPV reporting to police (1·02 [1·00–1·03]) increased one day after, and helpline calls (1·01 [1·00–1·03]) increased five days after heatwaves.^62^ Data on the 2009 Black Saturday bushfires in Australia showed a similar trend indicating that women in high-bushfire-affected communities experienced higher levels of violence than communities that were not affected by bushfires.^63^, ^64^ Yet, although there might be increased or new violence, formal reporting might not always occur.^64^, ^65^

Several mechanisms could explain a potential connection between extreme events and GBV. Evidently, extreme events disrupt everyday life and often result in economic and food insecurity.^39^, ^48^, ^58^, ^60^ In many patriarchal societies, men provide financial sustenance whereas women are considered dependents.^46^, ^48^, ^58^, ^60^ Consequently, during economic hardship, households might arrange early marriages as a method of financially coping.^41^, ^52^ Another commonly documented driving factor was an increase in daily (ie, mental) stressors and a loss of agency, control, and protective structures leading to people lashing out in new or escalated patterns of violence.^3^, ^34^, ^37^, ^42^, ^48^, ^56^, ^64^, ^65^ These mental stressors were often due to a general inability to cope emotionally or was spurred by specific hardships, such as men reacting violently after coming home to no food.^46^, ^48^, ^65^

Extreme events also appeared to enable environments and increase opportunities for perpetrators to commit violence, such as through perpetrators’ increased access to women in emergency shelters or relief workers requesting sexual favours in exchange for aid. In another context, the cover of helping out after an extreme event enabled a previous abuser to re-enter a woman's life.^65^ Similarly, the need to prove one's masculinity after having been unable to protect their community and family from harm could lead to an increase in GBV.^46^, ^48^, ^64^, ^65^ Damage to both physical and social infrastructures also created increased opportunities for violence.^3^, ^34^, ^38^, ^40^, ^42^, ^47^, ^48^, ^52^ For example, flooded streets and electricity outages exposed women's bodies by soaking their clothing, made running away more difficult, and removed possible protection from bystanders.^3^, ^47^, ^48^

## Discussion

This systematic review analysed the peer-reviewed and grey literature evidence on extreme events and GBV experienced by women, girls, and sexual and gender minorities. Across the 41 included publications, GBV during and post-extreme event appears to be a recurring theme. Several forms and drivers of GBV, perpetrated by both close relationships and strangers, were reported in the majority of included quantitative publications.^29^, ^31^, ^32^, ^33^, ^36^, ^41^, ^43^, ^45^, ^46^, ^49^, ^50^, ^51^, ^52^, ^54^, ^55^, ^57^, ^59^, ^61^, ^62^, ^63^, ^66^ Despite this evidence, some studies reported no significant difference or no increase in GBV,^30^, ^35^, ^44^, ^50^, ^53^, ^55^, ^56^, ^61^ and some studies showed a decrease in sexual assault^59^ and child marriage.^57^ Qualitative studies similarly showed increases in violence and its drivers during and after extreme events.^3^, ^8^, ^34^, ^37^, ^38^, ^42^, ^43^, ^45^, ^47^, ^48^, ^52^, ^56^, ^58^, ^60^, ^64^, ^65^, ^66^, ^67^ While considering the limitations of available data, the results suggest that extreme events can be associated with GBV. High-quality evidence from large, ethnographically diverse cohorts is essential to further explore the mechanisms and underlying driving factors of GBV during and after extreme events.

The findings of this Review align with existing reviews on violence following disasters, which have identified general violence trends to be exacerbated as a consequence of natural disasters.^11^ Comparing GBV and general violence following disasters, the drivers of violence overlap (ie, economic shock, social instability, enabling environments, and stress). Further evidence from a recent study exploring the literature on natural hazards and climate disasters and violence against women and girls (VAWG) suggested that risk factors for post-disaster VAWG included unequal social norms, gender inequalities, increased life stressors, law enforcement failures, and exposure to high-risk environments.^12^ Likewise, a recent scoping review on climate migration indicated that migrating women were more vulnerable to several GBV forms including forced marriage and sexual violence.^97^ Our study contributes to the evidence base by using a broad definition of GBV, including sexual and gender minorities, searching a wide set of databases as recently as February, 2022, and focusing on extreme events that are expected to increase with climate change, providing a comprehensive and timely analysis.

The relationship between extreme events and GBV can be expected to vary across settings due to differences in social gender norms, tradition, vulnerability, exposure, adaptive capacity, available reporting mechanisms, and legal responses. However, the experience of GBV during and after extreme events seems to be a shared experience in most contexts studied, suggesting that amplification of GBV is not constrained geographically. Yet, notably extreme events do not cause GBV; rather, extreme events exacerbate drivers of violence or create enabling environments for this behaviour. The primary causes are systematic social and patriarchal structures enabling and normalising GBV.^65^

### Unmasking of existing violence

An important consideration is whether extreme events affect the occurrence or the reporting of GBV. Some studies suggest that extreme events could increase reporting, unmasking existing violence. Living through extreme events led some victims to feel they could no longer endure abuse or feel less inhibited to report the abuse than before the event. Additionally, coming together as a community in the aftermath of the Black Friday fires increased the visibility of violence.^65^ Simultaneously, GBV reporting is plagued by the silencing of victims (especially in countries where safeguarding a daughter's and family's honour, and the daughter's dignity and marriageability is important), fears of coming forward, failures of law enforcement, unwillingness to believe victims, and the normalisation of violence. These factors could lead to under-reporting and an underestimation of the true effect of extreme events on GBV.^54^ These specificities complicate the elucidation of the relationship between extreme events and GBV, especially when research is dependent on official reporting.

### The double burden of vulnerability

Existing social roles and norms combined with other forms of inequity leading to marginalisation, discrimination, and dispossession make women and sexual and gender minorities disproportionately vulnerable to extreme events.^98^ Importantly, the experience of GBV might further increase vulnerability, resulting in a so-called double burden of vulnerability.^99^ When faced with the likelihood of experiencing harassment or sexual violence in relief camps, some women or sexual and gender minorities choose to stay home or return to their homes even before doing so is safe.^38^, ^46^, ^48^ This method of protecting themselves from violence can place people in additional danger from extreme events and further restrict their already limited access to relief resources.^38^, ^46^, ^48^ Sometimes, promises of needed recovery aid from a donor agency disappeared after being sexually threatened by a relief employee, causing women to fear and avoid the agency, thus losing the assistance provided to other cyclone victims.^38^ Literature on participatory power indicates that women play important roles as users of energy, climate activists, and in the implementation of climate adaptation and mitigation strategies.^9^, ^10^ Yet their increased vulnerability interrupts their adaptation and mitigation capacities in extreme event management and risk reduction.^41^

### Sexual and gender minorities

Reflecting the majority of global health literature, our review is predominantly focused on cisgender women and girls, missing sexual and gender minorities perspectives and effects.^100^ Yet, although only one of the included studies explicitly addressed the needs of sexual and gender minorities, extreme events might influence particular GBV risks for individuals with diverse sexual and gender identities (eg, transgender, gender non-conforming, lesbian, and gay people). Due to their frequent marginalisation, sexual and gender minorities are often severely affected by disasters.^98^, ^101^ Likewise, due to the consideration of binary sex and gender systems and hetero-cis-normative societies, these groups tend to be at high risk of GBV. This GBV risk is emphasised by the numerous news and case reports covering how social inequalities, discrimination, and violence have worsened during natural disasters for sexual and gender minorities. To illustrate, the New Orleans gay community was blamed for Hurricane Katrina as it being God's punishment,^102^, ^103^ same-sex couples were prevented from receiving relief from the Federal Emergency Management Agency,^104^ transgender people were threatened in shelters or prohibited access after a natural disaster,^93^ and LGBTQI people experienced physical harm and violence in postdisaster shelters.^105^ Further research on extreme events and equitable sexual-transformative and gender-transformative interventions should therefore focus on engaging all genders and addressing inequities within sociopolitical systems.^106^

### Extreme event interventions

Several detection and attribution studies have linked the variability and intensity of extreme events with climate change, as shown in the 2020 report of *The Lancet* Countdown.^107^ Notably, the effect of GBV in these settings extends beyond violence and therefore contributes to downstream health-related consequences.^8^ However, only few intervention strategies recognise these long-term effects and the importance of gender-equitable norms.^8^, ^45^, ^47^ Disaster management interventions before, during, and following extreme events ought to focus on preventing, mitigating, and adapting to drivers of GBV using a sexual-transformative and gender-transformative approach that recognises the slow onset of GBV and its effects. GBV guidelines for conflict-inflicted settings, such as the Inter-Agency Standing Committee guidelines,^108^ could be applied in these settings as well.

The implementation and effect of disaster-related interventions are influenced by local sexual and gender cultures, emphasising the need to account for local norms, traditions, and social attitudes in the design and operation of programmes.^38^ To design socially inclusive interventions they need to be informed by the communities and, more specifically, the women, girls, and sexual and gender minority populations affected. Examples of such interventions could be providing post-disaster shelters and relief services (including toilets and bath areas) designed to be exclusively accessed by women, girls, and sexual and gender minorities^40^ or providing emergency response teams with sexual-transformative and gender-transformative training to promote GBV prevention.^56^ Incorporating community engagement in both design and implementation of these interventions enables the ability to account for context-specific nuances and understanding and appropriately responding to the needs of affected populations.^97^, ^109^ Likewise, empowerment initiatives for women and sexual and gender minorities that challenge regressive gender norms to reduce vulnerability could bring opportunities to negotiate their circumstances and bring positive change.^48^, ^110^ For example, women's groups using participatory-learning-action cycles facilitated by local peers have been used to sustainably improve reproductive and maternal health by enabling women to identify and prioritise local challenges and solutions.^111^ Similar programmes could be adapted and applied in extreme event management to empower women as decision makers in local communities.^110^, ^112^

### Quality of the literature

The quality and rigour across studies were not consistent, including a number of studies being assessed as poor quality. Only a few studies provided an analysis of pre-extreme-event GBV, and the included study designs were inherently plagued by various types of bias. Few studies used validated scales of measuring GBV, and the experience of the disaster was often assessed by proxy (eg, living in a flood-prone area). Furthermore, the quantitative studies almost solely relied on self-reporting—the resulting recall bias and participants’ ability to respond to questions would affect reported outcomes. Notably, the imbalanced power dynamics involved with GBV can result in under-reporting and respondent bias; as such, there could be an incomplete assessment of GBV that occurred before, during, or after extreme events. In qualitative research, potential biases from the researcher, such as confirmation bias, influence how the researcher interprets and presents data. Finally, we cannot rule out that publication bias could have affected the studies retrieved in this Review, as we were unable to assess publication bias using a funnel plot, which would require at least ten unique quantitative studies with similar outcome and exposure.^113^ These factors complicated robust information synthesis and point to a need for high quality, longitudinal studies to appropriately evaluate this intersection of GBV and extreme events.

### Strengths and limitations

The primary strengths of this study are the synthesis of both peer-reviewed and grey literature, the detailed comprehensive search strategy to gather available evidence, and the inclusion of various extreme events and GBV outcomes. As a result, this systematic review provides a holistic perspective of the potential relationship between extreme events and GBV globally. However, in addition to the quality of the included studies, there are also a few limitations. Due to their diverse nature, comparing experiences of GBV and extreme events in different socio-cultural and political settings between studies, and even between participants within the studies, is difficult to do without risking oversimplification that could overlook some nuances (for example an increase or decrease in child marriage could be dependent on the direction of payment involved). Further complicating this synthesis are the differences in the way GBV is defined and measured cross-nationally. We also recognise that the databases used might be bias towards English-language publications and publications from high-income countries.

## Conclusion

In conclusion, this Review offers a glimpse into the connection between extreme events and GBV, highlighting an often overlooked public health impact of climate change. Given the anticipated acceleration of weather and climate shocks, further high-quality quantitative and qualitative research with ethnographically diverse, longitudinal cohorts comparing changes in GBV levels before, during, and after an event is imperative. Further research can include the use of more advanced statistical techniques that allow for the estimation of causal effects. Further studies could elucidate mechanisms through which extreme events link to GBV and inform the design and implementation of climate-resilient, context-specific, and sexual-responsive and gender-responsive interventions that serve the needs of women, girls, and sexual and gender minorities globally.

## Declaration of interests

The authors declare that the research was done in the absence of any commercial or financial relationship that could be construed as a potential conflict of interest. KRvD received funding from the Gates Cambridge Trust (OP114) for her PhD studies. All other authors declare no competing interests.

## References

[1] Field CB, Barros V, International Panel on Climate Change (2012).

[2] UN Office for Disaster Risk Reduction (2019). The human costs of disasters: an overview of the last 20 years 2000–2019. https://www.undrr.org/media/48008/download.

[3] Nguyen HT (2018). Gendered vulnerabilities in times of natural disasters: male-to-female violence in the Philippines in the aftermath of super typhoon Haiyan. Violence Against Women.

[4] Bell JE, Brown CL, Conlon K (2018). Changes in extreme events and the potential impacts on human health. J Air Waste Manag Assoc.

[5] The UN Refugee Agency Gender-based violence. https://www.unhcr.org/en-us/gender-based-violence.html.

[6] Stark L, Ager A (2011). A systematic review of prevalence studies of gender-based violence in complex emergencies. Trauma Violence Abuse.

[7] Heise L, Ellsberg M, Gottmoeller M (2002). A global overview of gender-based violence. Int J Gynaecol Obstet.

[8] Tanyag M (2018). Resilience, female altruism, and bodily autonomy: disaster-induced displacement in post-Haiyan Philippines. Signs (Chic).

[9] Dankelman I (2010). Climate change: learning from gender analysis and women's experiences of organising for sustainable development. Gend Dev.

[10] Boyd E (2010). The Noel Kempff project in Bolivia: gender, power, and decision-making in climate mitigation. Gend Dev.

[11] Rezaeian M (2013). The association between natural disasters and violence: a systematic review of the literature and a call for more epidemiological studies. J Res Med Sci.

[12] Thurston AM, Stöckl H, Ranganathan M (2021). Natural hazards, disasters, and violence against women and girls: a global mixed-methods systematic review. BMJ Glob Health.

[13] Rubenstein BL, Lu LZN, MacFarlane M, Stark L (2020). Predictors of interpersonal violence in the household in humanitarian settings: a systematic review. Trauma Violence Abuse.

[14] Vu A, Adam A, Wirtz A (2014). The prevalence of sexual violence among female refugees in complex humanitarian emergencies: a systematic review and meta-analysis. PLoS Curr.

[15] Sánchez OR, Vale DB, Rodrigues L, Surita FG (2020). Violence against women during the COVID-19 pandemic: an integrative review. Int J Gynaecol Obstet.

[16] Peterman A, O'Donnell M (2020). COVID-19 and violence against women and children a third research round up for the 16 days of activism. https://www.cgdev.org/publication/covid-19-and-violence-against-women-and-children-third-research-round-16-days-activism.

[17] Moher D, Liberati A, Tetzlaff J, Altman DG (2009). Preferred reporting items for systematic reviews and meta-analyses: the PRISMA statement. BMJ.

[18] Page MJ, McKenzie JE, Bossuyt PM (2021). The PRISMA 2020 statement: an updated guideline for reporting systematic reviews. BMJ.

[19] Keygnaert I, Dias SF, Degomme O (2015). Sexual and gender-based violence in the European asylum and reception sector: a perpetuum mobile?. Eur J Public Health.

[20] O'Malley J, Holzinger A (2018). The Sustainable Development Goals: sexual and gender minorities. https://www.undp.org/sites/g/files/zskgke326/files/publications/SDGs_SexualAndGenderMinorities.pdf.

[21] Easterling D, Rusticucci M, Semenov V, Field CB, Barros V, Stocker TF, Dahe Q (2012). Managing the risks of extreme events and disasters to advance climate change adaptation.

[22] Seneviratne SI, Zhang X, Adnan M, Masson-Delmotte V, Zhai P, Pirani A (2021). Climate change 2021: the physical science basis contribution of Working Group I to the sixth assessment report of the Intergovernmental Panel on Climate Change.

[23] Adams J, Hillier-Brown FC, Moore HJ (2016). Searching and synthesising ‘grey literature’ and ‘grey information’ in public health: critical reflections on three case studies. Syst Rev.

[24] Critical Appraisal Skills Programme CASP checklists. https://casp-uk.net/casp-tools-checklists/.

[25] Wells GA, Shea B, O'Connell D The Newcastle-Ottawa Scale (NOS) for assessing the quality of nonrandomised studies in meta-analyses. http://www.ohri.ca/programs/clinical_epidemiology/oxford.asp.

[26] Long HA, French DP, Brooks JM (2020). Optimising the value of the critical appraisal skills programme (CASP) tool for quality appraisal in qualitative evidence synthesis. Research Methods in Medicine & Health Sciences.

[27] Noyes J, Booth A, Flemming K (2018). Cochrane qualitative and implementation methods group guidance series-paper 3: methods for assessing methodological limitations, data extraction and synthesis, and confidence in synthesized qualitative findings. J Clin Epidemiol.

[28] Tyndall J (2013). Grey literature for health research: a vital resource. https://canberra.libguides.com/c.php?g=599348&p=4148869.

[29] Anastario M, Shehab N, Lawry L (2009). Increased gender-based violence among women internally displaced in Mississippi 2 years post-Hurricane Katrina. Disaster Med Public Health Prep.

[30] Fagen JL, Sorensen W, Anderson PB (2011). Why not the University of New Orleans? Social disorganization and sexual violence among internally displaced women of Hurricane Katrina. J Community Health.

[31] Harville EW, Taylor CA, Tesfai H, Xu Xiong, Buekens P (2011). Experience of Hurricane Katrina and reported intimate partner violence. J Interpers Violence.

[32] Picardo CW, Burton S, Naponick J (2010). Physically and sexually violent experiences of reproductive-aged women displaced by Hurricane Katrina. J La State Med Soc.

[33] Schumacher JA, Coffey SF, Norris FH, Tracy M, Clements K, Galea S (2010). Intimate partner violence and Hurricane Katrina: predictors and associated mental health outcomes. Violence Vict.

[34] Thornton WE, Voigt L (2007). Disaster rape: vulnerability of women to sexual assaults during Hurricane Katrina. J Public Manag Soc Policy.

[35] Temple JR, van den Berg P, Thomas JF, Northcutt J, Thomas C, Freeman DH (2011). Teen dating violence and substance use following a natural disaster: does evacuation status matter?. Am J Disaster Med.

[36] Westhoff WW, Lopez GE, Zapata LB, Corvin JAW, Allen P, Mcdermott RJ (2008). Reproductive health education and services needs of internally displaced persons and refugees following disaster. Am J Health Educ.

[37] Bermudez LG, Stark L, Bennouna C (2019). Converging drivers of interpersonal violence: findings from a qualitative study in post-hurricane Haiti. Child Abuse Negl.

[38] Rezwana N, Pain R (2020). Gender-based violence before, during and after cyclones: slow violence and layered disasters. Disasters.

[39] Nguyen H, Rydström H (2018). Climate disaster, gender, and violence: men's infliction of harm upon women in the Philippines and Vietnam. Womens Stud Int Forum.

[40] Houghton R, Wilson T, Smith W, Johnston D (2010). Domestic violence reporting ‘if there was a dire emergency, we never would have been able to get in there’: domestic violence reporting and disasters. Int J Mass Emerg Disasters.

[41] Asadullah MN, Islam KMM, Wahhaj Z (2020). Child marriage, climate vulnerability and natural disasters in coastal Bangladesh. J Biosoc Sci.

[42] Fothergill A (1999). An exploratory study of woman battering in the Grand Forks flood disaster: implications for community responses and policies. Int J Mass Emerg Disasters.

[43] Azad AK, Hossain KM, Nasreen M (2014). Flood-induced vulnerabilities and problems encountered by women in northern Bangladesh. Int J Disaster Risk Sci.

[44] Frasier PY, Belton L, Hooten E (2004). Disaster down East: using participatory action research to explore intimate partner violence in eastern North Carolina. Health Educ Behav.

[45] Madhuri (2016). The impact of flooding in Bihar, India on women: a qualitative study. Asian Women.

[46] Memon FS (2020). Climate change and violence against women: study of a flood-affected population in the rural area of Sindh, Pakistan. Pakistan J Women's Stud Alam-e-Niswan.

[47] Rashid SF, Michaud S (2000). Female adolescents and their sexuality: notions of honour, shame, purity and pollution during the floods. Disasters.

[48] Singh D (2010). Gender relations, urban flooding, and the lived experiences of women in informal urban spaces. Asian J Women's Stud.

[49] Allen EM, Munala L, Henderson JR (2021). Kenyan women bearing the cost of climate change. Int J Environ Res Public Heal.

[50] Díaz JJ, Saldarriaga V (2020). A drop of love? Rainfall shocks and spousal abuse: evidence from rural Peru. SSRN.

[51] Miguel E (2005). Poverty and witch killing. Rev Econ Stud.

[52] Ahmed KJ, Haq SMA, Bartiaux F (2019). The nexus between extreme weather events, sexual violence, and early marriage: a study of vulnerable populations in Bangladesh. Popul Environ.

[53] Cools S, Flatø M, Kotsadam A (2020). Rainfall shocks and intimate partner violence in sub-Saharan Africa. J Peace Res.

[54] Epstein A, Bendavid E, Nash D, Charlebois ED, Weiser SD (2020). Drought and intimate partner violence towards women in 19 countries in sub-Saharan Africa during 2011–2018: a population-based study. PLoS Med.

[55] Cooper M, Sandler A, Vitellozzi S (2021). Re-examining the effects of drought on intimate-partner violence. PLoS One.

[56] Rai A, Sharma AJ, Subramanyam MA (2021). Droughts, cyclones, and intimate partner violence: a disastrous mix for Indian women. Int J Disaster Risk Reduct.

[57] Corno L, Hildebrandt N, Voena A (2020). Age of marriage, weather shocks, and the direction of marriage payments. Econometrica.

[58] Esho T, Komba E, Richard F, Shell-Duncan B (2021). Intersections between climate change and female genital mutilation among the Maasai of Kajiado County, Kenya. J Glob Health.

[59] Sekhri S, Storeygard A (2011). The impact of climate variability on crimes against women: dowry deaths. Economics.

[60] Hossen MA, Benson D, Hossain SZ, Sultana Z, Rahman MM (2021). Gendered perspectives on climate change adaptation: a quest for social sustainability in Badlagaree village, Bangladesh. Water.

[61] Carrico AR, Donato KM, Best KB, Gilligan J (2020). Extreme weather and marriage among girls and women in Bangladesh. Glob Environ Change.

[62] Sanz-Barbero B, Linares C, Vives-Cases C, González JL, López-Ossorio JJ, Díaz J (2018). Heat wave and the risk of intimate partner violence. Sci Total Environ.

[63] Molyneaux R, Gibbs L, Bryant RA (2019). Interpersonal violence and mental health outcomes following disaster. BJPsych Open.

[64] Parkinson D, Zara C (2013). The hidden disaster: domestic violence in the aftermath of natural disaster. Aust J Emerg Manag.

[65] Parkinson D (2019). Investigating the increase in domestic violence post disaster: an Australian case study. J Interpers Violence.

[66] International Federation of Red Cross and Red Crescent Societies Gender-based violence prevention and response during natural disasters. https://www.globalprotectioncluster.org/_assets/files/gbv_prevention_and_response_during_disasters1.pdf.

[67] Dwyer E, Woolf L (2018). Down by the river; addressing the rights, needs and strengths of Fijian sexual and gender minorities in disaster risk reduction and humanitarian response. https://www.edgeeffect.org/wp-content/uploads/2018/02/Down-By-The-River_Web.pdf.

[68] Bradley T, Martin Z, Upreti BR, Subedu B, Shrestha S (2021). Gender and disaster: the impact of natural disasters on violence against women in Nepal. J Asian Afr Stud.

[69] Campbell DW, Campbell JC, Yarandi HN (2016). Violence and abuse of internally displaced women survivors of the 2010 Haiti earthquake. Int J Public Health.

[70] Cerna-Turoff I, Kane JC, Devries K, Mercy J, Massetti G, Baiocchi M (2020). Did internal displacement from the 2010 earthquake in Haiti lead to long-term violence against children? A matched pairs study design. Child Abuse Negl.

[71] Chan KL, Zhang Y (2011). Female victimization and intimate partner violence after the May 12, 2008, Sichuan earthquake. Violence Vict.

[72] Irshad H, Mumtaz Z, Levay A (2012). Long-term gendered consequences of permanent disabilities caused by the 2005 Pakistan earthquake. Disasters.

[73] Fisher S (2010). Violence against women and natural disasters: findings from post-tsunami Sri Lanka. Violence Against Women.

[74] Kolbe AR, Hutson RA, Shannon H (2010). Mortality, crime and access to basic needs before and after the Haiti earthquake: a random survey of Port-au-Prince households. Med Confl Surviv.

[75] Lai BS, Osborne MC, De Veauuse-Brown N, Swedo EA, Self-Brown S, Massetti GM (2020). Violence victimization and negative health correlates of youth in post-earthquake Haiti: findings from the cross-sectional violence against children survey. J Affect Disord.

[76] Logie CH, Daniel C, Ahmed U, Lash R (2016). ‘Life under the tent is not safe, especially for young women’: understanding intersectional violence among internally displaced youth in Leogane, Haiti. Glob Health Action.

[77] Rao S (2020). A natural disaster and intimate partner violence: evidence over time. Soc Sci Med.

[78] Rahill GJ, Joshi M, Lescano C, Holbert D (2015). Symptoms of PTSD in a sample of female victims of sexual violence in post-earthquake Haiti. J Affect Disord.

[79] Rees S, Pittaway E, Bartolomei L (2005). Waves of violence: women in post-tsunami Sri Lanka. Australas J Disaster Trauma Stud.

[80] Sakurai K, Nishigori H, Nishigori T (2017). Incidence of domestic violence against pregnant females after the great east Japan earthquake in Miyagi prefecture: the Japan environment and children's study. Disaster Med Public Health Prep.

[81] Sloand E, Killion C, Yarandi H (2017). Experiences of violence and abuse among internally displaced adolescent girls following a natural disaster. J Adv Nurs.

[82] Sohrabizadeh S (2016). A qualitative study of violence against women after the recent disasters of Iran. Prehosp Disaster Med.

[83] Sohrabizadeh S, Jahangiri K, Jazani RK, Babaie J, Moradian MJ, Rastegarfar B (2017). Women's challenges and capabilities in disasters: a case report of the twin earthquakes of eastern Azerbaijan, Iran. PLoS Curr.

[84] Standing K, Parker S, Bista S (2016). Grassroots responses to violence against women and girls in post-earthquake Nepal: lessons from the field. Gend Dev.

[85] Subedi S, Davison C, Bartels S (2020). Analysis of the relationship between earthquake-related losses and the frequency of child-directed emotional, physical, and severe physical abuse in Haiti. Child Abuse Negl.

[86] Tanoue K, Nishigori H, Watanabe Z (2021). Interannual changes in the prevalence of intimate partner violence against pregnant women in Miyagi prefecture after the great east Japan earthquake: the Japan Environment and Children's Study. J Interpers Violence.

[87] Tearne JE, Guragain B, Ghimire L, Leaning J, Newnham EA (2021). The health and security of women and girls following disaster: a qualitative investigation in post-earthquake Nepal. Int J Disaster Risk Reduct.

[88] Yoshihama M, Yunomae T, Tsuge A, Ikeda K, Masai R (2019). Violence against women and children following the 2011 great east Japan disaster: making the invisible visible through research. Violence Against Women.

[89] Weitzman A, Behrman JA (2016). Disaster, disruption to family life, and intimate partner violence: the case of the 2010 earthquake in Haiti. Sociol Sci.

[90] International Gay and Lesbian Human Rights Commission/SEROVie (2011). The impact of the earthquake, and relief and recovery programs on Haitian LGBT people. https://outrightinternational.org/sites/default/files/504-1.pdf.

[91] Human Rights Watch (2011). “Nobody remembers us”. Failure to protect women's and girls' right to health and security in post-earthquake Haiti. https://www.hrw.org/report/2011/08/19/nobody-remembers-us/failure-protect-womens-and-girls-right-health-and-security.

[92] Margi N (2010). In post-earthquake Haiti, activists fight violence based on gender and sexuality. https://observers.france24.com/en/20101114-post-earthquake-haiti-activists-fight-violence-based-gender-sexuality.

[93] Fontanez JA (2019).

[94] Met office Attributing extreme weather to climate change. https://www.metoffice.gov.uk/research/climate/understanding-climate/attributing-extreme-weather-to-climate-change.

[95] van Oldenborgh GJ, van der Wiel K, Kew S (2021). Pathways and pitfalls in extreme event attribution. https://www.worldweatherattribution.org/pathways-and-pitfalls-in-extreme-event-attribution/.

[96] Swain DL, Singh D, Touma D, Diffenbaugh NS (2020). Attributing extreme events to climate change: a new frontier in a warming world. One Earth.

[97] van Daalen KR, Dada S, Issa R (2021). A scoping review to assess sexual and reproductive health outcomes, challenges and recommendations in the context of climate migration. Front Glob Womens Health.

[98] van Daalen K, Jung L, Dhatt R, Phelan AL (2020). Climate change and gender-based health disparities. Lancet Planet Health.

[99] Gender-Based Violence Area of Responsibility Helpdesk Climate change and gender based violence: what are the links?. https://gbvaor.net/sites/default/files/2021-03/gbv-aor-helpdesk-climate-change-gbv-19032021.pdf.

[100] Pillay SR, Ntetmen JM, Nel JA (2022). Queering global health: an urgent call for LGBT+ affirmative practices. Lancet Glob Health.

[101] Gaillard JC, Gorman-Murray A, Fordham M (2017). Sexual and gender minorities in disaster. Gend Place Cult.

[102] Chrisafis A (2005). Katrina ‘sent by God to punish New Orleans gays’. https://www.theguardian.com/uk/2005/nov/19/northernireland.hurricanes2005.

[103] Pittman A (2011). Televangelist Rick Joyner: God sent Katrina because of the gays. https://www.lgbtqnation.com/2011/06/televangelist-rick-joyner-god-sent-katrina-because-of-the-gays/.

[104] pamindurham (2005). Katrina exposes gay couples' second-class status. https://www.dailykos.com/stories/2005/9/5/145686/-.

[105] Thuringer C (2016). Left out and behind: fully incorporating gender into the climate discourse. https://www.newsecuritybeat.org/2016/08/left-behind-fully-incorporating-gender-climate-discourse/.

[106] Smyth M, Jenness V, Gartner R, McCarthy B (2014). The Oxford Handbook of Gender, Sex, and Crime.

[107] Watts N, Amann M, Arnell N (2020). The 2020 report of the *Lancet* Countdown on health and climate change: responding to converging crises. Lancet.

[108] Inter-Agency Standing Committee (2015). Guidelines for integrating gender-based violence interventions in humanitarian action reducing risk. https://gbvguidelines.org/wp/wp-content/uploads/2015/09/2015-IASC-Gender-based-Violence-Guidelines_lo-res.pdf.

[109] Ashworth HC, Dada S, Buggy C, Lees S (2021). The importance of developing rigorous social science methods for community engagement and behavior change during outbreak response. Disaster Med Public Health Prep.

[110] Prost A, Colbourn T, Seward N (2013). Women's groups practising participatory learning and action to improve maternal and newborn health in low-resource settings: a systematic review and meta-analysis. Lancet.

[111] Dada S, Tunçalp Ö, Portela A, Barreix M, Gilmore B (2021). Community mobilization to strengthen support for appropriate and timely use of antenatal and postnatal care: a review of reviews. J Glob Health.

[112] Aziz A, Shams M, Khan KS (2011). Participatory action research as the approach for women's empowerment. Action Res (Lond).

[113] Macaskill P, Walter SD, Irwig L (2001). A comparison of methods to detect publication bias in meta-analysis. Stat Med.

