# Supplementary material for: Extreme events and gender-based violence: a mixed-methods systematic review
Source: Lancet Planet Health. 2022 Jun 13;6(6):e504–23. doi: 10.1016/S2542-5196(22)00088-2 (PMC10073035; doi:10.1016/S2542-5196(22)00088-2)
Supplement: Supplementary appendix [file mmc1.pdf]

THE LANCET
Planetary Health

Supplementary appendix

This appendix formed part of the original submission and has been peer reviewed. We post it as supplied by the authors.

Supplement to: van Daalen KR, Kallesøe SS, Davey F, et al. Extreme events and gender-based violence: a mixed-methods systematic review. *Lancet Planet Health* 2022; **6:** e504–23

Appendix

to van Daalen, K.R., Kallesøe, S. S., Davey, F., Jung, L., Singh, L., Issa, R., Emilian, C. A., Kuhn, I., Keygnaert, I., Nilsson, M. (2022). Extreme events and gender-based violence: a mixed-methods systematic review. *The Lancet* Planetary Health

Supplementary Table 1. Preferred Items for Systematic Reviews and Met-analyses (PRISMA) Checklist.[1,2]

Section and Topic	Item #	Checklist item	Location where item is reported
**TITLE**			
Title	1	Identify the report as a systematic review.	Page 1
**ABSTRACT**			
Abstract	2	See the PRISMA 2020 for Abstracts checklist.	Page 3
**INTRODUCTION**			
Rationale	3	Describe the rationale for the review in the context of existing knowledge.	Page 4
Objectives	4	Provide an explicit statement of the objective(s) or question(s) the review addresses.	Page 4
**METHODS**			
Eligibility criteria	5	Specify the inclusion and exclusion criteria for the review and how studies were grouped for the syntheses.	Page 5-6
Information sources	6	Specify all databases, registers, websites, organisations, reference lists and other sources searched or consulted to identify studies. Specify the date when each source was last searched or consulted.	Page 5
Search strategy	7	Present the full search strategies for all databases, registers and websites, including any filters and limits used.	Page 5-6<br>Supplement table 1
Selection process	8	Specify the methods used to decide whether a study met the inclusion criteria of the review, including how many reviewers screened each record and each report retrieved, whether they worked independently, and if applicable, details of automation tools used in the process.	Page 5-6
Data collection process	9	Specify the methods used to collect data from reports, including how many reviewers collected data from each report, whether they worked independently, any processes for obtaining or confirming data from study investigators, and if applicable, details of automation tools used in the process.	Page 6
Data items	10a	List and define all outcomes for which data were sought. Specify whether all results that were compatible with each outcome domain in each study were sought (e.g., for all measures, time points, analyses), and if not, the methods used to decide which results to collect.	Page 6
	10b	List and define all other variables for which data were sought (e.g., participant and intervention characteristics, funding sources). Describe any assumptions made about any missing or unclear information.	Page 6
Study risk of bias	11	Specify the methods used to assess the risk of bias in the included studies, including details of the tool(s) used, how many reviewers assessed each study and	Page 6

assessment		whether they worked independently, and if applicable, details of automation tools used in the process.	
Effect measures	12	Specify for each outcome the effect measure(s) (e.g., risk ratio, mean difference) used in the synthesis or presentation of results.	Page 6
Synthesis methods	13a	Describe the processes used to decide which studies were eligible for each synthesis (e.g., tabulating the study intervention characteristics and comparing against the planned groups for each synthesis (item #5)).	Page 6
	13b	Describe any methods required to prepare the data for presentation or synthesis, such as handling of missing summary statistics, or data conversions.	Page 6
	13c	Describe any methods used to tabulate or visually display the results of individual studies and syntheses.	Page 6
	13d	Describe any methods used to synthesize results and provide a rationale for the choice(s). If meta-analysis was performed, describe the model(s), method(s) to identify the presence and extent of statistical heterogeneity, and software package(s) used.	Page 6
	13e	Describe any methods used to explore possible causes of heterogeneity among study results (e.g., subgroup analysis, meta-regression).	Page 6
	13f	Describe any sensitivity analyses conducted to assess the robustness of the synthesized results.	Page 6
Reporting bias assessment	14	Describe any methods used to assess the risk of bias due to missing results in a synthesis (arising from reporting biases).	Page 6
Certainty assessment	15	Describe any methods used to assess certainty (or confidence) in the body of evidence for an outcome.	Page 6
**RESULTS**			
Study selection	16a	Describe the results of the search and selection process, from the number of records identified in the search to the number of studies included in the review, ideally using a flow diagram.	Page 7, Figure 1
	16b	Cite studies that might appear to meet the inclusion criteria, but which were excluded, and explain why they were excluded.	NA
Study characteristics	17	Cite each included study and present its characteristics.	Page 7-8
Risk of bias in studies	18	Present assessments of risk of bias for each included study.	Page 7
Results of individual studies	19	For all outcomes, present, for each study: (a) summary statistics for each group (where appropriate) and (b) an effect estimate and it's precision (e.g., confidence/credible interval), ideally using structured tables or plots.	Table 1-5
Results of syntheses	20a	For each synthesis, briefly summarise the characteristics and risk of bias among contributing studies.	Supplement tables 2-3
	20b	Present results of all statistical syntheses conducted. If meta-analysis was done, present for each the summary estimate and its precision (e.g., confidence/credible interval) and measures of statistical heterogeneity. If comparing groups, describe the direction of the effect.	NA
	20c	Present results of all investigations of possible causes of heterogeneity among study results.	Page 7-12
	20d	Present results of all sensitivity analyses conducted to assess the robustness of the synthesized results.	NA
Reporting biases	21	Present assessments of risk of bias due to missing results (arising from reporting biases) for each synthesis assessed.	NA
Certainty of evidence	22	Present assessments of certainty (or confidence) in the body of evidence for each outcome assessed.	NA
**DISCUSSION**			

Discussion	23a	Provide a general interpretation of the results in the context of other evidence.	Page 14
	23b	Discuss any limitations of the evidence included in the review.	Page 12-17
	23c	Discuss any limitations of the review processes used.	Page 16
	23d	Discuss implications of the results for practice, policy, and future research.	Page 17
**OTHER INFORMATION**			
Registration and protocol	24a	Provide registration information for the review, including register name and registration number, or state that the review was not registered.	Page 5
	24b	Indicate where the review protocol can be accessed, or state that a protocol was not prepared.	Page 5
	24c	Describe and explain any amendments to the information provided at registration or in the protocol.	NA
Support	25	Describe sources of financial or non-financial support for the review, and the role of the funders or sponsors in the review.	Page 21
Competing interests	26	Declare any competing interests of review authors.	Page 21
Availability of data, code and other materials	27	Report which of the following are publicly available and where they can be found: template data collection forms; data extracted from included studies; data used for all analyses; analytic code; any other materials used in the review.	Page 7

Supplementary Table 2. Search Strategy used on 2nd February 2022

**Database**	
**PubMed**<br><br>N= 6,828	((women OR "Sexual and Gender Minorities"[Mesh] OR woman OR girl OR non binary OR genderfluid OR female* OR transsexual or trans or transgender OR gender[MeSH Terms] OR homosexual OR bisexual OR asexual OR gay OR lesbian OR intersex OR Two-Spirit OR same-sex or "Sexual Behavior"[Mesh] OR "Transgender Persons"[Mesh] OR "Sexuality"[Mesh] OR "Transsexualism"[Mesh] OR "Gender Role"[Mesh] OR "Gender Identity"[Mesh]) AND ("extreme weather"[Mesh] OR "natural disasters"[Mesh] OR disaster* OR hurricane* OR typhoon* OR storm* OR tornado* OR blizzard* OR flood* OR drought* OR cyclone* OR wind* OR thunder OR waterspout OR fog OR supercell or supercells OR rain* OR wind* OR heatwave* OR coldwave* OR wildfire* OR weather* OR "climate change" OR "cold wave" OR "heat wave" OR "extreme cold" OR "extreme heat" OR "extreme temperature" OR "wild fire" OR "heavy precipitation") or "global warming"[mesh]) AND (violence OR "GBV" OR "IPV" OR "FGM" OR abuse OR intimidate or intimidation OR bully or bullying OR rape* OR battering OR stalk or stalking OR hack or hacking or hacked or cyberstalk or cyberstalking or (surveillance not public health) OR (surveilling NOT public health)OR beat OR harass OR "female genital mutilation" OR "genital cutting" OR "child marriage" OR femicide OR filicide OR "Sex Offenses"[Mesh] or "Gender-Based Violence"[Mesh] or "battered women"[Mesh] or "Stalking"[Mesh])
**Ovid MEDLINE**<br><br>N= 3,775	1. (wom?n* or girl* or female* or transgender* or trans or homosexual* or bisexual* or asexual* or gay or lesbian or intersex or two-spirit or same-sex or transsexual* or non binary or nonbinary or genderfluid*).mp. or exp women/ or exp "sexual and gender minorities"/ or exp transgender persons/ or exp transsexualism/ or exp gender role/ or exp gender identity/<br><br>2. (disaster* or hurricane* or typhoon* or storm* or tornado* or blizzard* or flood* or drought* or cyclone* or wind* or thunder* or waterspout* or fog* or supercell* or rain* or wind* or heatwave* or coldwave* or wildfire* or weather* or climate change or cold wave or heat wave or extreme cold or extreme heat or extreme temperature or wild fire or heavy precipitation) .mp. or exp extreme weather/ or exp natural disasters/ or exp climate change/ or exp global warming/<br><br>3. (violen* or GBV or IPV or FGM or abus* or intimidat* or bully* or rape* or batter* or stalk* or beat* or harass* or "female genital mutilation" or (female adj2 circumcis*) or "genital cutting" or "child marriage" or femicide or filicide or hack* or cyberstalk* or surveillance or surveilling).mp. or exp sex offenses/ or exp Gender-Based Violence/ or exp domestic violence/ or exp circumcision, female/ or exp bullying/ or exp intimate partner violence/ or exp spouse abuse/ or exp battered women/ or exp stalking/<br><br>4. 1 and 2 and 3
**Ovid Embase**<br><br>N=1,666	1. (wom?n* or girl* or female* or transgender* or transsexual* or non binary or nonbinary or genderfluid* or trans or homosexual* or bisexual* or asexual* or gay or lesbian or intersex or two-spirit or same-sex).ti,ab. or exp *female/ or exp *"sexual and gender minority"/ or exp *transgender/ or exp *transsexualism/ or exp *sex role/ or exp *gender identity/<br><br>2. (disaster* or hurricane* or typhoon* or storm* or tornado* or blizzard* or flood* or drought* or cyclone* or wind* or thunder* or waterspout* or fog* or supercell* or rain* or wind* or heatwave* or coldwave* or wildfire* or weather* or "climate change" or "cold wave" or "heat wave" or "extreme cold" or "extreme heat" or "extreme temperature" or "wild fire" or "heavy precipitation").ti,ab. or exp *extreme weather/ or exp *natural disasters/ or exp *climate change/ or exp *severe weather/ or exp *wildfire/<br><br>3. (violen* or GBV or IPV or FGM or abus* or intimidat* or bully* or rape* or batter* or stalk* or beat* or harass* or "female genital mutilation" or (female adj2 circumcis*) or "genital cutting" or "child marriage" or femicide or filicide OR hack* or cyberstalk* or surveillance OR surveilling).ti,ab. or exp *sexual crime/ or exp *sexual violence/ or exp *rape/ or exp *sexual abuse/ or exp *Gender Based Violence/ or exp *domestic violence/ or exp *female genital mutilation/ or exp *bullying/ or exp *partner violence/ or exp *partner violence/ or exp *battered women/ or exp *stalking/<br><br>4. 1 and 2 and 3
**CINAHL via EbscoHost**<br><br>N= 2,281	S1 ((wom?n* or girl* or female* or transgender* or transsexual* or non binary or nonbinary or genderfluid* or trans or homosexual* or bisexual* or asexual* or gay or lesbian or intersex or two-spirit or same-sex) or (MH "Women+") OR (MH "LGBTQ Persons+") OR (MH "Transgender Persons+") OR (MH "Transsexuals") OR (MH "Sexual and Gender Minorities+") or (MH "Gender Role+") OR (MH "Gender Identity+"))<br><br>S2 ((disaster* or hurricane* or typhoon* or storm* or tornado* or blizzard* or flood* or drought* or cyclone* or wind* or thunder* or waterspout* or fog* or supercell* or rain* or wind* or heatwave* or coldwave* or wildfire* or weather* or climate change or cold wave or heat wave or extreme cold or extreme heat or extreme temperature or wild fire or heavy precipitation) or (MH "Disasters+") OR (MH "Natural Disasters+") OR (MH "Weather+") OR (MH "Climate Change+"))<br><br>S3 (violen* or GBV or IPV or FGM or abus* or intimidat* or bully* or rape* or batter* or stalk* or beat* or harass* or "female genital mutilation" or (female adj2 circumcis*) or "genital cutting" or "child marriage" or femicide or filicide OR hack* or cyberstalk* or surveillance OR surveilling) or (MH "Violence+") OR (MH "Domestic Violence+") OR (MH "Gender-Based Violence") OR (MH "Dating Violence") OR (MH "Intimate Partner Violence") OR (MH "Circumcision, Female") OR (MH "Bullying+") OR (MH "Battered Women") OR (MH "Stalking") OR (MH "Rape") **or** (MH "Stalking"))

	S4 s1 and s2 and s3
**Global Health via EbscoHost**<br><br>N= 984	S1 (wom?n* or girl* or female* or transgender* or transsexual* or non binary or nonbinary or genderfluid* or trans or homosexual* or bisexual* or asexual* or gay or lesbian or intersex or two-spirit or same-sex) or (DE "women" OR DE "employed women" OR DE "housewives" OR DE "lactating women" OR DE "rural women") OR (DE "girls")<br><br>S2 (disaster* or hurricane* or typhoon* or storm* or tornado* or blizzard* or flood* or drought* or cyclone* or wind* or thunder* or waterspout* or fog* or supercell* or rain* or wind* or heatwave* or coldwave* or wildfire* or weather* or climate change or cold wave or heat wave or extreme cold or extreme heat or extreme temperature or wild fire or heavy precipitation) or (DE "disasters" OR DE "natural disasters" OR DE "agricultural disasters")) OR (DE "natural disasters")) or (((DE "global warming") OR (DE "climatic change" OR DE "global warming")) OR (DE "storms" OR DE "dust storms" OR DE "hurricanes" OR DE "whirlwinds")) OR (DE "weather" OR DE "fire weather")<br><br>S3 (violen* or GBV or IPV or FGM or abus* or intimidat* or bully* or rape* or batter* or stalk* or beat* or harass* or "female genital mutilation" or (female n2 circumcis*) or "genital cutting" or "child marriage" or femicide or filicide OR hack* or cyberstalk* or surveillance OR surveilling) or (((DE "abuse" OR DE "child abuse" OR DE "sexual abuse" OR DE "spouse abuse" OR DE "substance abuse") OR (DE "spouse abuse")) OR (DE "rape")) OR (DE "circumcision")<br><br>S4 S1 AND S2 AND S3
**Scopus**<br><br>N= 5,549	TITLE-ABS-KEY (wom?n* OR girl* OR female* OR transgender* OR transsexual* OR "non binary" OR nonbinary OR genderfluid* or trans or homosexual* or bisexual* or asexual* or gay or lesbian or intersex or two-spirit or same-sex) AND TITLE-ABS-KEY (disaster* OR hurricane* OR typhoon* OR storm* OR tornado* OR blizzard* OR flood* OR drought* OR cyclone* OR wind* OR thunder* OR waterspout* OR fog* OR supercell* OR rain* OR wind* OR heatwave* OR coldwave* OR wildfire* OR weather* OR "climate change" OR "cold wave" OR "heat wave" OR "extreme cold" OR "extreme heat" OR "extreme temperature" OR "wild fire" OR "heavy precipitation") AND TITLE-ABS-KEY (violen* OR gbv OR ipv OR fgm OR abus* OR intimidat* OR bully* OR rape* OR batter* OR stalk* OR beat* OR harass* OR "female genital mutilation" OR (female W/2 circumcis*) OR "genital cutting" OR "child marriage" OR femicide OR filicide OR hack* or cyberstalk* or ((surveillance OR surveilling) and not "public health"))
**Web of Science Core Collection**<br><br>N= 2,097	#1<br>TS=(wom?n* or girl* or female* or transgender* or transsexual* or non binary or nonbinary or genderfluid* or trans or homosexual* or bisexual* or asexual* or gay or lesbian or intersex or two-spirit or same-sex)<br><br>**#2** TS=(disaster* or hurricane* or typhoon* or storm* or tornado* or blizzard or flood* or drought or cyclone* or wind* or thunder* or waterspout* or fog or supercell* or rain* or wind* or heatwave* or coldwave* or wildfire* or weather* or climate change or cold wave or heat wave or extreme cold or extreme heat or extreme temperature or wild fire or heavy precipitation)<br><br>**#3** TS=(violen* or GBV or IPV or FGM or abus* or intimidat* or bully* or rape* or batter* or stalk* or beat* or harass* or "female genital mutilation" or (female near2 circumcis*) or "genital cutting" or "child marriage" or femicide or filicide OR hack* or cyberstalk* or ((surveillance OR surveilling) not "public health"))<br><br>#4 #3 AND #2 AND #1
**SciELO via Web of Science**<br><br>N= 115	#1<br>TS=(wom?n* or girl* or female* or transgender* or transsexual* or non binary or nonbinary or genderfluid* or trans or homosexual* or bisexual* or asexual* or gay or lesbian or intersex or two-spirit or same-sex)<br><br>#2<br>TS=(disaster* or hurricane* or typhoon* or storm* or tornado* or blizzard* or flood* or drought* or cyclone* or wind* or thunder* or waterspout* or fog* or supercell* or rain* or wind* or heatwave* or coldwave* or wildfire* or weather* or c limate change or cold wave or heat wave or extreme cold or extreme heat or extreme temperature or wild fire or heavy pre cipitation)<br><br>#3<br>TS=(violen* or GBV or IPV or FGM or abus* or intimidat* or bully* or rape* or batter* or stalk* or beat* or harass* or " female genital mutilation" or (female near2 circumcis*) or "genital cutting" or "child marriage" or femicide or filicide OR hack* or cyberstalk* or surveillance OR surveilling)<br><br>#4<br>#3 AND #2 AND #1
**PsycINFO**<br><br>N= 3,041	S1 (wom?n* or girl* or female* or transgender* or transsexual* or non binary or nonbinary or genderfluid* or trans or homosexual* or bisexual* or asexual* or gay or lesbian or intersex or two-spirit or same-sex) or (DE "Human Females" OR DE "Battered Females" OR DE "Daughters" OR DE "Female Criminal Offenders" OR DE "Mothers" OR DE "Sisters" OR DE "Widows" OR DE "Wives" OR DE "Working Women") OR (DE "Transgender") or (DE "Sex Roles") OR (DE "Gender Identity" OR DE "Cisgender" OR DE "Gender Nonbinary" OR DE "Gender Nonconforming" OR DE "LGBTQ" OR DE "Transsexualism")

	S2 (disaster* or hurricane* or typhoon* or storm* or tornado* or blizzard* or flood* or drought* or cyclone* or wind* or thunder* or waterspout* or fog* or supercell* or rain* or wind* or heatwave* or coldwave* or wildfire* or weather* or climate change or cold wave or heat wave or extreme cold or extreme heat or extreme temperature or wild fire or heavy precipitation) or (((DE "Disasters" OR DE "Natural Disasters") OR (DE "Atmospheric Conditions" OR DE "Climate Change")) OR (DE "Global Warming")) OR (DE "Climate Change" OR DE "Global Warming")<br><br>S3 (violen* or GBV or IPV or FGM or abus* or intimidat* or bully* or rape* or batter* or stalk* or beat* or harass* or "female genital mutilation" or (female n2 circumcis*) or "genital cutting" or "child marriage" or femicide or filicide OR hack* or cyberstalk* or surveillance OR surveilling) or (DE "Violence" OR DE "Dating Violence" OR DE "Domestic Violence" OR DE "Gun Violence" OR DE "Intimate Partner Violence" OR DE "Patient Violence" OR DE "Police Violence" OR DE "Political Violence" OR DE "School Violence" OR DE "Violent Crime" OR DE "Virtual Violence" OR DE "Workplace Violence") OR (DE "Rape" OR DE "Acquaintance Rape") OR (DE "Battered Females") OR (DE "Domestic Violence") OR (DE "Circumcision") or DE "Stalking"<br><br>S4 S1 AND S2 AND S3
**LILACS**<br><br>N= 45	women or girls or transgender or transsexual or lesbian or gay [Palavras] and violence or rape or assault or batter or abuse OR hack or cyberstalk or surveillance OR surveilling [Palavras] and weather or storm or drought or fire or flood or disaster [Palavras]

Supplementary Table 3. Grey Literature Search Strategy used on 10 February 2022

**Organisations**	**Website**
CARE International	https://www.careinternational.org.uk/
Gender-Based Violence AoR	https://gbvaor.net/
Gender, Diversity and Inclusion	https://www.cgiar.org/how-we-work/accountability/gender-diversity-and-inclusion/
Human Rights Watch	https://www.hrw.org/
Human Rights Campaign	https://www.hrc.org/
Intergovernmental Panel on Climate Change	https://www.ipcc.ch/
International Planned Parenthood Federation	https://www.ippf.org/
International Organisation for Migration	https://www.iom.int/
International Rescue Committee	https://www.rescue-uk.org/
LGBTQ Health Education Center	https://www.lgbtqiahealtheducation.org/
National Sexual Violence Resource Center	https://www.nsvrc.org/
Reliefweb	https://reliefweb.int/
United Nations Development Program	https://www.undp.org/
United Nations Office for Disaster Risk Reduction	https://www.undrr.org/
United Nations Women	https://www.unwomen.org/en
World Health Organisation	https://www.who.int/
World Meteorological Organisation	https://public.wmo.int/en
**Google search**	
Search terms used	
extreme weather violence filetype:pdf	Searched through pages 1-5 of results
extreme weather rape filetype:pdf	
extreme weather sexual assault filetype:pdf	
extreme weather domestic violence filetype:pdf	
extreme climate violence filetype:pdf	
extreme climate rape filetype:pdf	
extreme climate sexual assault filetype:pdf	
extreme climate domestic violence filetype:pdf	
natural disasters violence filetype:pdf	
natural disasters domestic violence filetype:pdf	
natural disasters sexual assault filetype:pdf	
natural disasters rape filetype:pdf	

Supplementary Table 4. Results and main conclusions of the included quantitative records (n=21)

Study	Extreme event	Violence	N participants	Results	Main conclusion
**Peer-reviewed**					
Anastario *et al.* 2009[3]	Hurricane Katrina (2005)	Physical intimate partner violence (IPV) and sexual GBV	2006: 106 women<br>2007: 314 women	*Crude rates of overall GBV*<br>-Adult lifetime: 4.6/100,000 per day (95% CI: 4.1/100,000 - 5.0/100,000)<br>-Post-disaster (2006): 16.3/100,000 per day (95% CI: 4.8/100,000 - 27.8/100,000)<br>-In 2007: 10.1/100,000 per day in 2007 (95% CI: 6.6/100,000 - 13.7/100,000)<br><br>*Crude rates of sexual violence*<br>-Adult lifetime: 3.05/100,000 per day (95% CI: 2.6/100,000 - 3.5/100,000)<br>-Post-disaster (2006): 14.4/100,000 per day (95% CI: 3.2/100,000 - 25.7/100,000)<br>-In 2007: 1.3/100,000 per day (95% CI: 0.0/100,000 - 2.6/100,000)<br><br>*Crude rates of intimate partner violence*<br>-Adult lifetime: 3.06/100,000 per day (95% CI: 2.7/100,000 - 3.5/100,000)<br>-Post-disaster (2006): 9.4/100,000 per day (95% CI: 0.6/100,000 - 18.2/100,000)<br>-In 2007: 10.1/100,000 per day (95% CI: 6.6/100,000 - 13.7/100,000)	The rate of GBV, specifically IPV, increased the year after Hurricane Katrina among internally displaced women and did not return to baseline in the two years following Hurricane Katrina.
Picardo *et al.* 2010[4]	Hurricane Katrina (2005)	Physical and sexual abuse	66 women	*Percentage of women reporting (95% CI)*<br>-Being hit or verbally threatened since hurricane Katrina: 23% (14-34%)<br>-Abuse increased after Katrina by 33% (13-63%)<br>-Abuse decreased after Katrina by 13% (4-37%)<br>-Abuse by new partner 20% (6-51%)<br>-Abuse by same partner 13% (4-39%)	Following Hurricane Katrina, it was not uncommon to experience physical abuse among displaced women. New abuse or increasing abuse were most reported.
Fagen *et al.* 2011[5]	Hurricane Katrina (2005)	Sexual violence	2005: 237 women<br>2006: 215 women	-No significant differences in any of the measures of sexual violence toward women (nine CORE survey items) pre- to post-Katrina.	There is no evidence to suggest a significant difference in sexual violence prevalence before or after Hurricane Katrina among female University of Orleans students.
Harville *et al.* 2011[6]	Hurricane Katrina (2005)	Intimate partner violence	123 women postpartum women	*Adjusted relative risk (RR) for being*<br>-Insulted, sworn, shouted, or yelled at: 1.23, 95% CI: 1.02 - 1.48<br>-Pushed, shoved, or slapped: 5.28, 95% CI: 1.93 - 14.45<br>-Punched, kicked, or beat up: 8.25, 95% CI: 1.68 - 40.47	The results suggest that certain exposures during Hurricane Katrina, such as experiencing damage to one's home during the storm, are associated with an increased risk of IPV.
Schumacher *et al.* 2010[7]	Hurricane Katrina (2005)	Interpersonal violence (physical and psychological)	251 women, 194 men	*Prevalence of IPV*<br>-Percentage of women reporting psychological victimization increased from 33.6% to 45.2% following Hurricane Katrina ($p < .001$).<br>-Reports of physical victimization increased from 4.2% to 8.3% for women ($p = .01$).	There is evidence to suggest that psychological victimization reports among women following Hurricane Katrina increased significantly compared to before the disaster.
Temple *et al.* 2011[8]	Hurricane Katrina (2005)	Teen dating violence (TDV)	584 girls, 464 boys	*Adjusted OR (95% CI) TDV of non-evacuation*<br>-TDV Victimization: 0.62 (0.22–1.77)<br>-TDV Sexual Victimization: 1.27 (0.33–4.86)	There is no evidence to suggest that the odds of experiencing teen dating violence among adolescent girls significantly differed between those who were evacuated due to Hurricane Ike and those who were not evacuated.
Westhoff *et al.* 2008[9]	Hurricane Mitch (1998)	Sexual and domestic violence	54 women	*Prevalence of sexual and domestic violence against internally displaced women*<br>-Forced sex against will: 37.2%<br>-Traded sex for supplies: 25.9%<br>-Partner/husband or another household member hitting: 13.3%	Sexual and domestic violence against internally displaced women following Hurricane Mitch were frequently reported.

Frasier *et al.* 2004[10]	Floods	Physical, verbal, and threatening intimate partner violence	785 women	-Of those affected by the flood, 11 women (4% of the total) reported physical abuse after the flood. 15 women (3%) who were not affected by the flood also reported physical abuse after Floyd.<br>-These results also held for comparisons of pre-flood and post-flood experience of psychological and verbal IPV.	There is no evidence to suggest that the prevalence of IPV significantly differed between women affected by the flood following Hurricane Floyd and those who were not affected. However, the prevalence of IPV in both the affected and unaffected groups is alarmingly high, suggesting IPV to be endemic among the sampled blue-collar female workers.
Allen *et al.* 2021[11]	Severe weather events are defined as floods lasting > 10 days (2006-2014)	Intimate partner violence: physical and sexual violence and emotional abuse	2008: 4,903 women<br>2014: 4,512 women	*Odds ratio (95% CI) of reporting intimate partner violence (IPV)*<br>-Counties experiencing severe weather events vs counties that did not: 1.60 (1.35-1.89)<br><br>*Odds ratio (95% CI) of reporting due to an increase in the number of floods*<br>-IPV in all forms: 1.74 (1.43-2.13)<br>-Physical violence: 2.01 (1.63-2.49)<br>-Sexual violence: 1.48 (1.07-2.05)<br>-Emotional abuse: 1.03 (0.82-1.30)<br><br>*Odds ratio (95% CI) of reporting due to experience of severe floods*<br>-IPV in all forms: 1.60 (1.35-1.89)<br>-Physical violence: 1.91 (1.59-2.29)<br>-Sexual violence: 1.61 (1.22-2.12)	There may be a relationship between severe weather events and experiencing IPV, with increases reported for physical and sexual violence. Reported IPV differed across regions, suggesting different factors (e.g., economic) which may influence IPV experience.
Díaz & Saldarriage 2020[12]	Rainfall shocks (drought and floods)	Physical, sexual and emotional intimate partner violence	15,110 women (640 exposed to flood event, 421 exposed to drought event)	*β of physical IPV after drought event*<br>-Pushed/shook: 0.054 (0.026) (*P*-value<0.05)<br>-Slapped: 0.065 (0.022) (*P*-value<0.01)<br>-Punched: 0.032 (0.028)<br>-Kicked/dragged: 0.018 (0.027)<br>-Moderate P-IPV: 0.081 (0.034) (*P*-value<0.05)<br><br>*β of physical IPV after flood event*<br>-Pushed/shook: -0.001(0.023)<br>-Slapped: 0.016 (0.020)<br>-Punched: 0.028 (0.023)<br>-Kicked/dragged: 0.027 (0.020)<br>-Moderate P-IPV: -0.006 (0.024)	Following the exposure of a drought event, physical IPV increased by 65%, but not for a flood event. As the rise of physical IPV accompanies income decline, economic security may be central in explaining the relationship. Two other factors may also influence the IPV increase including reduction of women's empowerment and reduced emotional wellbeing of men.
Miguel *et al.* 2005[13]	Extreme rainfall (drought or flood)	Witch killing, elderly women murder	67 villages; 5-15 village officials interviewed; rainfall data from the station in the district capital; 1,293 households (15-20 per village)	-Extreme rainfall is associated with 0.085 more witch murders per village per year (significant at 95% confidence), implying that there are twice as many witch murders in years of extreme rainfall as in other years.<br>-Drought and flood both have a similar impact on murders—with point estimates of 0.099 and 0.080, respectively.	There is evidence to suggest that the murder of "witches" (elderly women) in Tanzania largely increased following extreme rainfall events, including droughts and floods. No similar increase in other types of murders was observed during the same time frame.
Cools et al. 2020[14]	Rainfall shocks, drought	Intimate partner violence	*Complete cross-sectional sample*: 149,032 women.<br>*Nine country sample:* 9 countries with repeated surveys.<br>*Event history analysis:* 50,512 women	*Complete cross-sectional sample*<br>-No significant correlation was found between IPV at the α = 2.5% or the 10% level during the last rainy season; the occurrence of floods was associated with an increase in IPV.<br><br>*Nine countries with repeated surveys*	There is no evidence to suggest a positive association between droughts and IPV. The authors suggest that this may be because rainfall shocks do not affect spousal power or because the relatively slow onset of drought (compared to other weather crises, such as storms) affords a calmer response to the distress.

				-No significant correlation between IPV and droughts was found, except for a positive effect of droughts on IPV at the α = 2.5% level.<br><br>*Event history analysis*<br>-Respondents reported when their first experience of IPV occurred. The results suggest a risk reduction of IPV occurring for the first time within the marriage due to droughts.	
Epstein *et al.* 2020[15]	Drought	Intimate partner violence	83,990 partnered women of which 9,019 experienced severe drought and 19639 experienced mild/moderate drought	*Marginal risk difference estimates in percentage points of women in severe drought (compared to not experiencing drought)*<br>-Controlling partner (marginal RD = 3.0, 95% CI: 1.3-4.6; p < 0.001)<br>-Emotional violence (marginal RD = 0.4, 95% CI: -0.5-1.3; p > 0.05)<br>-Physical violence (marginal RD = 0.8, 95% CI: 0.1−1.5; p = 0.019)<br>-Sexual violence (marginal RD = 1.2, 95% CI: 0.4−2.0; p = 0.001)<br><br>*Women living in mild/moderate drought had higher risk of:*<br>-Controlling partner (marginal RD = 0.0, 95% CI: -1.1−1.2; p > 0.05)<br>-Physical violence (marginal RD = 0.7, 95% CI: 0.2−1.1; p = 0.003)<br>-Sexual violence (marginal RD = 0.7, 95% CI: 0.3−1.2; p = 0.001)<br>-No evidence for associations between drought and emotional violence.	The results suggest that drought is associated with increased reports of a controlling partner and experiencing physical and sexual violence among women. This association was strong among more vulnerable populations, such as adolescent girls and unemployed women. There is no evidence to suggest that drought is associated with reported emotional violence. The associations between drought and violence varied across countries, with drought seemingly protective for at least one form of violence in Uganda, Namibia, and Tanzania.
Cooper *et al.* 2021[16]	Extreme rainfall/droughts	Intimate partner violence: physical violence, sexual violence, emotional violence, controlling behaviours	363,428 women from 40 countries	-Models suggest no clear association between drought and the experience of emotional, physical and sexual violence on any of the included continents.<br>-Droughts had a strong association with controlling behaviours on all included continents.	When controlling for spatial autocorrelation, little association between drought and most forms of IPV can be found. Yet, some evidence of drought and controlling behaviour perpetrated by the women's partner can be observed.
Corno *et al.* 2020[17]	Extreme rainfall/drought (referred to as weather shocks)	Early/child marriage (marriage before the age of 18)	400,000 women	-In Sub-Saharan Africa, girls who experience a drought (12-17 yrs) are 0.2–0.26pp more likely to get married in the same year. Effect corresponds to a 2.3–3% increase in the annual hazard of child marriage in response to a drought.<br>-Floods did not affect child marriage in Sub-Saharan Africa.<br>-In India, girls who experience a drought (12-17 yrs) are 0.46–0.47pp less likely to get married in that same year. Effect corresponds to a 4.2%-4.3% decline in the annual hazard of child marriage in response to drought.<br>-Floods reduce child marriage hazard in India.<br>-Model indicates a negative relationship between droughts and child marriage in countries where a dowry needs to be paid by the girls' families.	Short-term changes in economic conditions (e.g., drought/flood) impact the age of marriage. Whether this results in an increase or decrease in child marriage is dependent on marriage payments.
Sekhri & Storeygard 2011[18]	Dry shocks (below-average rainfall)	Crimes against women including dowry deaths, domestic violence, sexual harassment, murder, kidnapping	Not applicable	*β Domestic violence controlled for total crimes and socio-economic factors*<br>-Dry shock: 0.183 (0.0696) (*P*-value<0.01)<br>-Wet shock: 0.002 (0.0340)<br><br>*β Dowry deaths controlled for total crimes and socio-economic factors*<br>-Dry shock: 0.291 (0.0972) (*P*-value<0.01)<br>-Wet shock: 0.074 (0.0557)<br><br>*β Sexual harassment controlled for total crimes and socio-economic factors*	Rainfall shocks have a significant effect on crimes against women. During dry shock, an increase in domestic violence, dowry deaths, and dowry payment is reported. In contrast, a decrease in sexual harassment is reported. Resorting to dowry killings to increase economic returns may be a consumption smoothing mechanism.

				-Dry shock: -0.649 (0.205) (*P*-value<0.01)<br>-Wet shock: 0.187 (0.138)	
Rai *et al.* 2020[19]	Drought and cyclones	Intimate partner violence	31,045: drought affected<br>8,469: cyclone affected<br>39514: total	*Adjusted Odds Ratio (OR) of exposure to cyclones*<br>-Emotional IPV: 1.59, 95% CI: 1.20-2.10<br>-Physical IPV: 1.25, 95% CI: 0.98-1.60<br>-Sexual IPV: 1.26, 95% CI: 0.79-2.01<br><br>*Adjusted Odds Ratio (OR) of exposure to drought*<br>-Emotional IPV: 0.99, 95% CI: 0.82-1.22<br>-Physical IPV: 1.12, 95% CI: 0.98-1.27<br>-Sexual IPV: 0.95, 95% CI: 0.76-1.20	Evidence suggests that exposure to cyclones is associated with an increased prevalence of emotional IPV after adjusting for socio-demographic factors. Non-significant positive associations were observed between cyclone exposure and physical and sexual IPV, as well as between drought exposure and physical IPV. There is no evidence to suggest an association between drought exposure and emotional or sexual IPV.
Carrico *et al.* 2020[20]	Heat waves, dry spells	Early and forced marriage		*Heatwaves, dry spells, and early marriage*<br>-The results suggest a positive linear relationship between heat waves and the risk of marriage in the subsequent year. A one standard deviation increase in the duration of heat waves was associated with a 17% increase in the risk of marrying in the next calendar year (OR = 1.167, standard error = 0.077)<br>-No significant association was found between dry spells and marriage.<br><br>*Heatwaves, dry spells, and conditions of marriage*<br>-There is weak evidence to suggest that heat waves are associated with an increased likelihood that a woman marries a man who endorses IPV (p = 0.15).<br>-The results suggest a significant relationship between dry spells and the risk of marrying a man who supports IPV.	The evidence suggests an increased risk of girls and women marrying in the year of and following heat waves; this trend is strongest amongst women aged 18-23 years old and weakest among those aged 11-14. The results indicate that women and girls who married during heat waves tended to marry poorer men with less education. The results also suggest that women and girls who married during dry spells tended to marry less-educated men who are more inclined to practice intimate partner violence. The authors suggest that these findings indicate the marriage of girls and women to be an economic coping mechanism for Bangladeshi families facing natural disasters.
Sanz-Barbero *et al.* 2018[21]	Heat waves	Intimate partner femicides (IPF), intimate partner violence (IPV)	Population of Madrid	*Relative Risk (RR) after the heatwave*<br>-IPF three days after: 1.40, 95% CI: 1.00 - 1.97<br>-IPV police reports one day after: 1.02, 95% CI: 1.00 - 1.03<br>-IPV helpline calls five days after: 1.01, 95% CI: 1.00 - 1.03<br><br>*Attributable risk (AR) among those exposed*<br>-IPF: 28.8%, 95% CI: 0.3% - 49.2%<br>-IPV police reports: 1.7%, 95% CI: 0.3% - 3.1%<br>-IPV helpline calls: 1.43%, 95% CI: 0.1% - 2.8%	The results suggest a positive association between heat waves and reports of IPV.
Molyneaux *et al.* 2020[22]	Wildfires	GBV with a focus on intimate partner violence	967 included in the analysis, 585 (60%) women	*Rates of participants reports of violence since the 2009 bushfires*<br>-High impact: 30 (7.4%)<br>-Medium impact: 0. (0.0%)<br>-Low impact: 1 (1.0%)<br>-Total: 31 (5.3%)	Women living in areas highly affected by bushfires were observed to experience the greatest prevalence of violence. The results suggest the experience of violence among women following the bushfire are associated with changes in income, as well as post-traumatic stress disorder and symptoms due to exposure to the bushfires.

Supplementary Table 5. Results and main conclusions of the included qualitative records (n=15)

Study	Extreme event	Violence	Results	Main conclusion
**Peer-reviewed**				
Bermudez *et al.* 2019[23]	Hurricane Matthew	Violence against women and children	The study identified multiple, intersecting drivers of violence against women influenced by Hurricane Matthew including: the accumulation of daily stressors, loss of power/control, learned behaviour (intergenerational cycle of abuse), need for behaviour regulation and inequitable gender norms.	Findings suggest multiple and converging drivers of violence in a humanitarian crisis context may be exacerbated and may require interdisciplinary responses due to their intersecting nature.
Thornston *et al.* 2007[24]	Hurricane Katrina (2005)	Sexual assault	Women's vulnerability to sexual assaults may be related to several conditions induced by Hurricane Katrina over different phases of the disaster. This includes the physical, social and structural failure of infrastructures such as a lack of social support, and the breakdown of the criminal justice system. Whilst speculative, the early phases of the disaster tend to show more brutal rapes. In recovery phases, however, rapes seemed to follow a more typical not disaster-related pattern.	Different phases of Hurricane Katrina provided opportunities for the victimisation of women. Assaults on women were facilitated by conditions in disaster shelters and temporary housing, breakdown of governmental leadership, a lack of social support, a loss of power, and exposure to collective groups of men.
Rezwana *et al.* 2020[25]	Cyclone Roanu (2016)	Gender-based violence (physical, sexual or mental harm)	The number and form of violence against women are believed to increase, from an already high baseline level, immediately before, during and after cyclones, starting from the moment the warnings were given. This includes sexual assault and also events of forced marriage.	Cyclones may, directly and indirectly, lead to increased rates of GBV, which in turn makes women more vulnerable during disaster situations. Impacts of GBV may therefore further increase poverty in women and gender inequalities.
Nguyen *et al.* 2018[26]	Typhoon Haiyan (also as Super Typhoon Yolanda) (2013)	Violence against women and girls (including domestic violence, intimate partner violence, sexual violence and incest)	The study suggests that GBV reaches its highest levels, in situations that are exceptional and potentially life-threatening and where the general law system and the main social infrastructures are out of work. This might be provoked by post-disaster stress, but is also shaped by Catholic ideas of gender roles within society.	GBV faced by women and girls is not a result of the disaster alone, but rooted in gender inequalities inherent to society, which may be exacerbated in times of crisis. Effective violence prevention must therefore begin before the disaster happens.
Nguyen & Rydstrom 2018[27]	Typhoon Haiyan (also as Super Typhoon Yolanda) (2013)	Intimate partner violence (e.g., beating women)	Whilst violence against women increased after Typhoon Haiyan, some report that this should be seen as a continuation of existing attitudes in societies that privilege men over women and stimulate a culture of acceptance. There is a reluctance to report violence as police may often side with the perpetrator, and victims may not be believed or may be blamed for the abuse. In both countries, several initiatives to end structural and physical violence include governmental campaigns, and women's organisations informing people about legal protection and rights. In Vietnam, pre-typhoon government strategies had a pre-emptive effect in reducing violence.	There is a need for context-informed research on the dynamics between GBV and climate disasters.
Tanyag *et al.* 2018[28]	Typhoon Haiyan (also as Super Typhoon Yolanda) (2013)	Sexual and gender-based violence	In disaster situations, high economic strain and stress increase the risk for GBV including domestic violence/marital rape. In IDP camps safety can't be guaranteed and sexual and GBV, including rape, happens routinely.	Women and girls are facing long-term harm in the displacement context due to increased levels of sexual and GBV. There is a risk of reinforcing determinants of gendered vulnerability in resilience discourses, including political, cultural, and economic barriers to accessing sexual and reproductive health services, leading to a restriction of body autonomy.
Madhuri *et al.* 2016[29]	Floods	Eve-teasing" (public sexual harassment) and other kinds of violence; verbal, physical, & sexual harassment & domestic violence	Floods lead to a change in patterns of verbal, sexual harassment and domestic violence against women, including high risk due to frustrated/stressed husbands incapable of providing for their families due to the disaster. In relief camps or shelters, public sexual harassment (e.g., eve-teasing) and other forms of harassment and physical violence against women are common occurrences. Long-term, flooding increases the economic dependency of women due to decreases in their sources of income, thus making them more vulnerable to GBV.	Flooding increases the risk for women to experience violence. Protection mechanisms in relief camps are missing and the dislocation makes women even more vulnerable to violence and harassment.

Rashid *et al.* 2000[30]	Floods	Sexual and mental harassment	Flood shelters and relief camps pose a high risk of sexual and mental harassment due to the lack of privacy and physical space. This leads to anxiety in girls with regard to issues of harassment, sexuality, and shame. Commonly prevalent levels of harassment were exacerbated during times of flooding and affecting them in their daily lives. One potential reason for increased harassment described was men loitering around more than usual due to work interruptions.	Adolescent girls show increased, distinct needs during disaster situations as they are at high risk to experience harassment leading to long-term mental harm. Central to their experiences and coping strategies during the floods are notions of honour, shame, purity and pollution, which affect their sexuality and gender relations
Singh *et al.* 2020[31]	Floods	Family conflict and violence	In times of flooding, women experience disturbed family environments posing challenges like increased familial and domestic conflict (including verbal fights), frustration and stress due to loss of income, property damages, food insecurity and illness. The impacts for women are deeply connected to traditional gender roles and inequality within patriarchal societies, which may exacerbate family conflicts and violence.	The combination of disaster situations and patriarchal structures within societies make women prone to experience family conflicts, verbal abuse and violence. Marital conflict and domestic violence, a frequent occurrence in male-dominated societies, become severe in times of disaster.
Forthergill 1999[32]	North Dakota flood (1997)	Domestic violence; women battering	There was an increase in women seeking protection from domestic violence post-flood compared to pre-flood. Two cases were discussed in the paper.<br>*Case 1:* The participant reported that the flood seemed to precipitate violence, the husband had a temper before but was only violent once, then not at all for 19 years of marriage, and then became violent after the floods.<br>*Case 2:* The participant had been in a long-standing abusive relationship and the flood helped her leave. She reports that the husband was away in substance abuse treatment at the time of the flood and managing the flood damage/impacts on her own showed her she was strong enough to leave him.	As illustrated by the two cases, violence occurring after/before a disaster could worsen due to exposure to the disaster, or the disaster may offer opportunities for women to leave the abusive relationship. Resources to battle domestic violence (e.g., counselling, emergency shelters) are important during the disaster period as demands for these services increase.
Esho *et al.* 2021[33]	Extreme rainfall/drought	Early marriage and female genital mutilation (cutting off parts of the female external genitalia for non-medical reasons)	As an adaptive strategy to climatic changes (extreme rainfall/drought), child marriages and FGM practises have increased, resulting in the disempowerment of women and girls. For example, when men had to walk further to feed cattle, they marry younger women to support in home-making duties. As uncut girls cannot be accepted as brides, increases in child marriages also resulted in FGM increases.	The prevalence of child marriage and FGM may be impacted by the intersection between climatic conditions and gender inequality. Strategies should focus on abandoning FGM, keeping girls in school and delaying marriage.
Hossen *et al.* 2021[34]	Drought	Early marriage, physical (e.g., kicking, shaking, pushing), emotional/psychological and sexual violence (e.g., rape), violence in the workplace	Marginalised women experience impacts of drought through dowry costs, early marriage and violence. Many of these vulnerabilities exist pre-drought, embedded in socio-cultural practise, yet they are magnified by environmental impacts. When agricultural production is lost, this may act as an incentive to marry off daughters as well as a way to maintain patriarchal order in society.	Due to the growing impacts of droughts, gendered vulnerabilities increase. Further research should explore how gendered vulnerabilities during drought are influenced by sociocultural constructions.
Parkinson & Zara 2013[35]	Black Saturday bushfires (2009)	Domestic violence	Out of 30 women, 17 experienced violence (9 of which did not experience violence before the fires). People experienced homelessness, unemployment, increased alcohol and drug use acting as stressors for violence. Men faced unattainable demands on their masculinity, leaving them feeling inadequate. Women tended to be silenced with societal pressure to deny and forgive violence. Shared emergency accommodation may expose women to unavoidable contact with abusive partners or ex-partners.	The aftermath of the Black Saturday bushfires display increases in domestic violence. The bushfires highlighted the difficulties in reporting domestic violence and deeply embedded male privilege.
Parkinson *et al.* 2019[36]	Black Saturday bushfires (2009)	Domestic/interpersonal violence	The fires lead to some women experiencing new violence in their relationship, while some women who had experienced violence before reported a sharp increase in the disaster situation. Women also experienced an increased vulnerability which kept or led them back into violent relationships.	While domestic violence against women increased post-disaster, support services are often overburdened with fire-related needs and do not provide adequate services for women in need.
**Grey literature**				
Dwyer and Woolf 2018[37]	Tropical cyclone Winston (2016)	Gendered violence against sexual and gender minorities (including e.g., domestic violence)	SGM were blamed for the tropical cyclone by religious leaders and local communities (their sexual orientation, gender identity or gender expression). The disaster was understood as God's punishment for their "sins". Assumptions underlying mainstream disaster management programs often inadvertently exclude SGM. Post-TC Winston, some gay men indicated to be uncomfortable sharing	One of the priority themes emerging from the story-sharing, community-mapping and traditional *Talanoa* sessions was violence, harassment and trauma. SGM are blamed for

(Oxfam research report)		shelters with heterosexual men due to fear of violence and discrimination. People reported feelings of powerlessness. Reporting family or community members to the authorities was not seen as a practical option as it could lead to longer-term family exclusion or other repercussions. Secret, serene places to console themselves or meet with other SGM were often mentioned by those experiencing prolonged violence. Violence and harassment resulted in stigma, isolation and trauma further limiting community participation of the SGM individuals.	causing TC Winston, experience violence, and are often isolated from social networks post-disaster.

Supplementary Table 6. Results and main conclusions of the included mixed-methods studies (n=6)

Study	Extreme event	Violence	Results	Main conclusion
**Peer-reviewed**				
Houghton *et al.* 2010[38]	Snowstorm	Domestic violence	*Quantitative:*<br>- After the snowstorm, while phone lines were down, domestic violence reporting through Women's Refuge reduced to 4 reports in June.<br>- After communications were restored, reports increased to 25, almost double the monthly average of 14.<br>- Of the women who reported and sought support services from Women's Refuge, 57.2% were first-time reporters.<br>- The most common duration of abuse among clients (65.9%) was between 2 and 10 years.	The June 2006 South Canterbury snowstorm demonstrated an increased need for domestic violence agency services due to stress in the community impacting violence levels. The interviews also revealed increased challenges to providing needed services during weather emergencies requiring improved emergency planning and policy responses.
			Qualitative: Stress was the top reason for the increase in domestic violence after the snowstorm. The reported causes of stress varied from financial burdens related to the storm, loss of income, hard labour to repair damage from the storm, childcare, isolation, and uncertainty. Interviews supported the idea that the extreme weather event likely served as both a trigger for increased violence within already abusive relationships as well as a lower tolerance for abuse and thus increased motivation to report and seek help. The snowstorm illuminated a lack of emergency plans among agencies, limited physical access to safehouses during the storm, inadequate supply of resources, and reduced staffing.	
Asadullah *et al.* 2020[39]	Salinity intrusion<br>Cyclone/storm surge<br>Tidal waters Flood<br>Riverbank erosion	Child marriage	*Quantitative:*<br>- 4 out of every 5 women were married before age 18, with an average age of 16 at marriage and almost 80% of marriages being arranged without their consent.<br>- Women in coastal villages (with higher threats of natural disasters) were more likely to have been married early, been married without a dowry, and for dowries paid to be lower amounts.<br>- The quantitative data suggested a positive association between shocks related to climate events and the incidence of child marriage.	The qualitative and quantitative evidence does not suggest that dowry-related factors are leading to early marriage. Rather, child marriage appears to be a coping strategy adopted by households in response to their increased vulnerability to natural disasters.
			Qualitative: Qualitative surveys found more than two-thirds of respondents having encountered at least one natural disaster event before marriage and related income shocks and vulnerability. Results from in-depth interviews revealed child marriage as a common practice across socio-economic and cultural groups. Multiple themes related to the causes of child marriage were indicated (such as economic vulnerability, coping with risk, family honour, and patriarchal norms), however natural disasters or climate changes were not directly connected to marriage timing by respondents.	
Azad *et al.* 2013[40]	Floods	Domestic violence, sexual violence, harassment	*Quantitative:*<br>- Harassment during and after floods was frequent and included mental, physical, and sexual harassment or violence.<br>- 64 women (35.46%) experienced harassment (n=185) during and after floods over the last five years. 58% were harassed by husbands, 58% by neighbours, 5% by unacquainted persons, 6% by boys or youth, and 3% by brothers-in-law.<br>- 33% of women experienced mental torture, >59% suffered verbal abuse, >34% encountered physical abuse, and 39% were beaten by their husbands.<br>- Women who worked also experienced harassment (83%) mostly by employers but also by co-workers.	Researchers found that floods create conditions that expose women to increased harassment and violence and also limit their ability to cope with the myriad risks posed in disaster settings. While men and women are both exposed to increased risks, women were found to be more vulnerable to flood impacts. Policies should be put in place to prevent violence against women and more generally reduce women's vulnerability such as by introducing home-based industries to reduce financial shocks and exposure to violence outside the home.
			Qualitative: Interviews revealed that sexual violence was rampant during the floods, sexual harassment of women also took place in shelter centres, and pregnant mothers suffered the worst during and after the	

			flooding. Women also faced harassment while seeking relief/assistance from passers-by, neighbours, NGO officers, and government officials.	
Memon *et al.* 2020[41]	Floods	Emotional violence, physical violence, sexual violence	*Quantitative:* 20% of interviewees referred to sexual harassment in camps and most did not report it.<br><br>*Qualitative:* During and after the floods, women were exposed to emotional, physical, and/or sexual violence. Experiences of emotional violence included abuse and ascribed responsibility for disaster-related conditions and hardships, spousal abandonment and increased care-taking or financial burdens, and shaming when women have to carry out socially masculine duties. Women continued to face physical violence from their husbands after being displaced by the floods and expressed a sense of helplessness with no means of escape as well as acceptance of the violence citing increased stress for their male family members because of the floods. Sexual violence was reported in interviews in relation to shelters and camps not being designed to keep women safe. Increased trafficking was also reported in the interviews.	Findings indicated increased emotional, physical, and sexual violence against women and that they are aware of the correlation between this violence and climate stress situations.
Ahmed *et al.* 2019[42]	Flash flooding, cyclones and floods related to cyclones	Child marriage, sexual violence	*Quantitative:* The age at first marriage was younger in the sample village (Alipur) with more vulnerability to flash floods than the village (Chandi) which experienced less disruption from floods.<br>- In Alipur, 86% (46/53) of married daughters were married before age 18, 38% of household heads mentioned a daughter experiencing sexual violence, and 48% reported fears about sexual violence their daughter(s) might face.<br>- In Chandi, 62% (58/93) of married daughters were married before age 18, 15% of household heads mentioned a daughter experiencing sexual violence, and 28% reported fears about sexual violence their daughter(s) might face.<br><br>*Qualitative:* Interviews indicated that household heads: viewed large families as a burden, particularly during extreme weather events; saw early marriage for their daughters as a means to reduce burdens; and reported arranging marriages as a response to experienced or threatened sexual violence that occurs in shelters or other locations during/after extreme weather events.	The arrangement of early/child marriages and the threat of or experiences of sexual violence was common in relation to extreme weather events. This study's findings indicated that early/child marriage was both a type of GBV experienced by girls as a coping mechanism for their families in the wake of economic burdens caused by extreme weather events and as a social protective mechanism against the threat of sexual violence or community response to actual sexual violence experiences.
**Grey literature**				
IFRC[43]	*Philippines:* Typhoon Haiyan (2013)<br>*Indonesia:* Western Nusa Tenggara floods (2017), Aceh Earthquake (2016)<br>*Lao PDR:* Oudomxay floods (2016), Typhoon Ketsana (2009)	Early marriage, domestic violence,	*Philippines:*<br>-After Typhoon Haiyan trafficking for sexual exploitation and abuse increased<br>-There is a need for improved guidelines of evacuation centres<br><br>*Indonesia*<br>-Increase of sexual harassment in shelters<br>-Perpetrators are strangers (17%), husbands (13%) and community members (30%)<br><br>*Lao PDR*<br>-Risk of GBV increasing from the first week to one-month post-disaster<br>-Factors influencing GBV: lack of employment, lack of security, abandonment of children	During natural disaster situations, the risk of GBV is increased. Yet, actors addressing the needs of violence survivors and disaster responders are not working together to appropriately reduce these risks.

Supplementary Table 7. List with articles excluded in full-text screening (n=125)

#	Article	Reason for exclusion
1	Alam, K., & Rahman, M. H. (2014). Women in natural disasters: a case study from southern coastal region of Bangladesh. International journal of disaster risk reduction, 8, 68-82.	Incorrect outcome (does not focus on GBV but women's preparedness for natural disasters)
2	Alston, M. (2015). Women and climate change in Bangladesh. Routledge.	Does not contain primary data/analysis
3	Alston, M., Whittenbury, K., Haynes, A., & Godden, N. (2014). Are climate challenges reinforcing child and forced marriage and dowry as adaptation strategies in the context of Bangladesh?. In Women's Studies International Forum (Vol. 47, pp. 137-144). Pergamon.	Incorrect outcome (does not focus on GBV but adaptive responses to climate challenges)
4	Anastario, M. P. (2007). An analysis of violence victimization and women's mental and reproductive health in two internally displaced populations. Boston College.	Incorrect outcome (does not focus on GBV, but women's mental and reproductive health)
5	Anastario, M. P., Larrance, R., & Lawry, L. (2008). Using mental health indicators to identify post-disaster gender-based violence among women displaced by Hurricane Katrina. Journal of Women's Health, 17(9), 1437-1444.	Incorrect outcome (does not focus on GBV, but on whether mental health indicators can be used to identify GBV)
6	Anastario, M., Shehab, N., & Lawry, L. (2009). Responding to gender-based violence in disasters. Disaster Medicine and Public Health Preparedness	Does not contain primary data/analysis
7	Anastario, M., Shehab, N., & Lawry, L. (2009). Responding to gender-based violence in disasters. Disaster medicine and public health preparedness, 3(3), 138-139.	Does not contain primary data/analysis
8	Anderson Hoffner, L., Simpson, J., Martinez-Fernandez, C., & Patumtaewapibal, A. (2021). Turning up the heat exploring potential links between climate change and gender-based violence and harassment in the garment sector (No. 995126893202676). International Labour Organization. Retrieved from: https://www.ilo.org/wcmsp5/groups/public/---asia/---ro-bangkok/documents/publication/wcms_792246.pdf	Does not contain primary data/analysis
9	Anderson, C. A., & DeLisi, M. (2011). Implications of global climate change for violence in developed and developing countries (p. 249). na.	Incorrect exposure (does not focus on extreme events, but rather temperature increase)
10	Atkinson, H. G., & Bruce, J. (2015). Adolescent girls, human rights and the expanding climate emergency.	Does not contain primary data/analysis
11	Auliciems, A., DiBartolo, L., 1995. Domestic violence in a subtropical environment: police calls and weather and Brisbane. Int. J. Biometeorol	Incorrect exposure (not extreme event)
12	Banford, A., Wickrama, T., Brown, M., & Ketring, S. (2011). The relationship between physical health problems and couple violence and conflict in survivors of the 2004 tsunami: Mediation by marital satisfaction. International Journal of Mass Emergencies & Disasters, 29, 149–170	Incorrect exposure (focus on tsunami)
13	Barnfonden. (2021). Exploring the link between climate change and violence against children. Retrieved from: https://barnfonden.se/app/uploads/2021/03/Investigating-climate-change-and-violence-against-children_FINAL-1.pdf	Does not contain primary data/analysis
14	Bhadra, S. (2021). Exploring dimensions of sexual issues in disasters and conflicts: Need to bridge the gaps between policy and practice. Sexologies.	Does not contain primary data/analysis

15	Bleeker, A., Escribano, P., Gonzales, C., Liberati, C., & Mawby, B. (2021). Advancing gender equality in environmental migration and disaster displacement in the Caribbean. Retrieved from: https://repositorio.cepal.org/bitstream/handle/11362/46737/1/S2000992_en.pdf	Does not contain primary data/analysis
16	Bradshaw, S., & Fordham, M. (2015). Double disaster: Disaster through a gender lens. In Hazards, risks, and disasters in society (pp. 233-251). Academic Press.	Does not contain primary data/analysis
17	Brezina, T., & Kaufman, J. M. (2008). What Really Happened in New Orleans? Estimating the Threat of Violence During the Hurricane Katrina Disaster∗. Justice Quarterly, 25(4), 701-722.	Incorrect outcome (does not focus on GBV, but the general threat of violence)
18	Buttell, F. P., & Carney, M. M. (2009). Examining the impact of Hurricane Katrina on police responses to domestic violence. Traumatology, 15(2), 6-9. (I)	Incorrect outcome (does not focus on GBV, but on whether the police responded to domestic violence)
19	Caldwell, J. 2006. “This is Home.” The Advocate, Sep., no. 12: 32–40.	Full text inaccessible/unavailable
20	Calma, J., (2018). Yes, trans rights are an environmental issues, too. Grist. Retrieved from: https://grist.org/article/transgender-rights-climate-intersectionality/	Does not contain primary data/analysis
21	Camey, I., Sabater, L., Owren, C., Boyer, A., & Wen, J. (2020). Gender-based violence and environment linkages. The Violence of Inequality; Wen, J., Ed.; IUCN: Gland, Switzerland.	Does not contain primary data/analysis (on extreme events and GBV)
22	Care International. Evicted by Climate Change: Confronting the Gendered Impacts of Climate-Induced Discplacement. Retrieved from: https://www.care-international.org/files/files/CARE-Climate-Migration-Report-v0_4.pdf	Does not contain primary data/analysis
23	Care International. We bend, we do not break: Resilient communities dealing with disaster and climate change. Retrieved from: https://www.care-international.org/files/files/publications/reports-issue-briefs/Bend-Not-Break-.pdf	Incorrect exposure (does not focus on extreme events, but climate change more broadly)
24	Caridade, S. M. M., Vidal, D. G., & Dinis, M. A. P. (2022). Climate Change and Gender-Based Violence: Outcomes, Challenges and Future Perspectives. In Sustainable Policies and Practices in Energy, Environment and Health Research (pp. 167-176). Springer, Cham.	Does not contain primary data/analysis
25	Chew L, Ramdas KN. (2005). Caught in the Storm: The Impact of Natural Disasters on Women. New York, NY: The Global Fund for Women.	Does not contain primary data/analysis
26	Clemens, P., Hietala, J. R., Rytter, M. J., Schmidt, R. A., & Reese, D. J. (1999). Risk of domestic violence after flood impact: Effects of social support, age, and history of domestic violence. Applied Behavioral Science Review.	Incorrect outcome (does not focus on GBV, but on domestic violence without a gender aspect)
27	Cohn, E.G., Rotton, J., 1997. Assault as a function of time and temperature: a moderator variable time-series analysis. J. Pers. Soc. Psychol	Incorrect exposure (not extreme event)
28	Consortium on Gender, Security, and Human Rights (2017. Environmental Disasters: Gendered Impacts & Responses Bibliography with Abstracts. Retrieved from: https://genderandsecurity.org/sites/default/files/Environmental_Disasters_Gendered_Impacts_Responses_-_Bibliography_with_Abstracts_-_CGSHR.pdf	Incorrect publication type (overview report only containing abstracts)
29	Curtis, T., Miller, B. C., & Berry, E. H. (2000). Changes in reports and incidence of child abuse following natural disasters. Child abuse & neglect, 24(9), 1151-1162.	Incorrect outcome (does not focus on GBV, but on child abuse without segregating results by sex or gender)

30	D’ooge, C. (2008). Queer Katrina: Gender and sexual orientation matters in the aftermath of the disaster. Katrina and the women of New Orleans, 22-24.	Does not contain primary data/analysis
31	David S. Blakeslee and Ram Fishman (2017): Weather Shocks, Agriculture, and Crime. Evidence from India. J. Human Resources Summer	Incorrect outcome (does not focus on GBV, but crime more broadly)
32	Desai, B. H., & Mandal, M. (2020). Role of climate change in exacerbating sexual and gender-based violence against women: A new challenge for international law. Environmental Policy and Law, (Preprint), 1-21.	Does not contain primary data/analysis
33	Dominey-Howes, D., (2021). ‘You never know if you will be treated properly and with respect’: voices of LGBTIQA+ people who lived through disasters. The Conversation. Retrieved from: https://theconversation.com/you-never-know-if-you-will-be-treated-properly-and-with-respect-voices-of-lgbtiqa-people-who-lived-through-disasters-153190	Does not contain primary data/analysis
34	Dominey-Howes, D., Gorman-Murray, A., & McKinnon, S. (2014). Queering disasters: On the need to account for LGBTI experiences in natural disaster contexts. Gender, Place & Culture, 21(7), 905– 918.	Does not contain primary data/analysis
35	Dominey-Howes, D., Gorman-Murray, A., & McKinnon, S. (2016). Emergency management response and recovery plans in relation to sexual and gender minorities in New South Wales, Australia. International Journal of Disaster Risk Reduction, 16, 1– 11.	Does not contain primary data/analysis
36	Duramy, Bendetta Faedi. (2014). “Women in the Aftermath of the Earthquake.” In Gender and Violence in Haiti: Women’s Path from Victims to Agents, 137–52. New Brunswick: Rutgers University Press.	Full text inaccessible/unavailable
37	Dwyer, E. (2021). The only way is up: Monitoring and encouraging diverse SOGIESC inclusion in the humanitarian and DRR sectors. UN Women. Retrieved from: https://asiapacific.unwomen.org/en/digital-library/publications/2021/03/the-only-way-is-up	Does not contain primary data/analysis
38	Eastin, J. (2018). Climate change and gender equality in developing states. World Development, 107, 289-305.	Incorrect outcome (does not focus on GBV)
39	Eastin, J. (2021). 8 Climate Change, Livelihoods and. Gender, Climate Change and Livelihoods: Vulnerabilities and Adaptations, 94.	Full text inaccessible/unavailable
40	Edwards, B., Gray, M., & Borja, J. (2021). The influence of natural disasters on violence, mental health, food insecurity, and stunting in the Philippines: Findings from a nationally representative cohort. SSM-population health, 15, 100825.	Incorrect outcome (does not focus on GBV, but on family violence without disaggregating by gender or sex)
41	Enarson, E., (2012). Does violence against women increase in disasters? Retrieved from: http://nhma.info/uploads/resources/gender/Does_WAW_Increase-july2011-ee.pdf	Does not contain primary data/analysis
42	Enarson, Elaine. (2006). Women Confronting Natural Disaster: From Vulnerability to Resilience. Boulder: Lynne Rienner Publishers.	Full text inaccessible/unavailable
43	Farmer, A. K., Zelewicz, L., Wachtendorf, T., & DeYoung, S. E. (2018). Scared of the shelter from the storm: Fear of crime and hurricane shelter decision making. Sociological Inquiry, 88(2), 193-215.	Incorrect outcome (does not focus on GBV, but overall fear of victimisation)
44	Eastin, J. (2021). 8 Climate Change, Livelihoods and. Gender, Climate Change and Livelihoods: Vulnerabilities and Adaptations, 94.	Full text inaccessible/unavailable

45	Edwards, B., Gray, M., & Borja, J. (2021). The influence of natural disasters on violence, mental health, food insecurity, and stunting in the Philippines: Findings from a nationally representative cohort. SSM-population health, 15, 100825.	Incorrect outcome (does not focus on GBV, but on family violence without disaggregating by gender or sex)
46	Enarson, E., (2012). Does violence against women increase in disasters? Retrieved from: http://nhma.info/uploads/resources/gender/Does_WAW_Increase-july2011-ee.pdf	Does not contain primary data/analysis
47	Enarson, Elaine. (2006). Women Confronting Natural Disaster: From Vulnerability to Resilience. Boulder: Lynne Rienner Publishers.	Full text inaccessible/unavailable
48	Fordham, Maureen. (2011). "Gender and Disasters." In Encyclopedia of Environmental Health, edited by J. O. Nriagu, 834–38. Burlington: Elsevier.	Full text inaccessible/unavailable
49	Fordham, Maureen. (2012). "Gender, Sexuality and Disaster." In The Routledge Handbook of Hazards and Disaster Risk Reduction, edited by Ben Wisner, J. C. Gaillard, and Ilan Kelman. Oxon: Routledge.	Full text inaccessible/unavailable
50	Gaillard, J. C., Gorman-Murray, A., & Fordham, M. (2017). Sexual and gender minorities in disaster. Gender, Place & Culture, 24(1), 18-26.	Incorrect outcome (does not focus on GBV)
51	Gearhart, S., Perez-Patron, M., Hammond, T. A., Goldberg, D. W., Klein, A., & Horney, J. A. (2018). The impact of natural disasters on domestic violence: An analysis of reports of simple assault in Florida (1999–2007). *Violence and gender*, *5*(2), 87-92.	Incorrect outcome (does not focus on GBV but simple assault)
52	Gender Based Violence AoR. Climate Change and Gender-based Violence: What are the links? UNICEF. Retrieved from: https://gbvaor.net/sites/default/files/2021-03/gbv-aor-helpdesk-climate-change-gbv-19032021.pdf	Does not contain primary data/analysis
53	Ghazvineh, Z. (2019). Natural disasters, social crises and violence against women. Two Quarterly Journal of Contemporary Sociological Research, 8(14), 229-259.	Does not contain primary data/analysis
54	Ginige, K., Amaratunga, D., & Haigh, R. (2014). Tackling women's vulnerabilities through integrating a gender perspective into disaster risk reduction in the built environment. Procedia Economics and Finance, 18, 327-335.	Incorrect outcome (does not focus on GBV)
55	Gorman-Murray, A., Dominey-Howes, D., & McKinnon, S. (2019). LGBTI experiences of disasters in the Antipodes. The Gender Security Project.	Full text inaccessible/unavailable
56	Gorman-Murray, A., McKinnon, S., Dominey-Howes, D., Nash, C. J., & Bolton, R. (2018). Listening and learning: Giving voice to trans experiences of disasters. Gender, Place & Culture, 25(2), 166-187.	Incorrect outcome (does not focus on GBV)
57	Habtezion, S. (2013). Overview of linkages between gender and climate change. Policy Brief. United Nations Development Programme, New York, 20. Retrieved from: https://www.undp.org/sites/g/files/zskgke326/files/publications/Gender_Climate_Change_Training%20Module%201%20Overview.pdf	Does not contain primary data/analysis
58	Hamel, L., Firth, J., Brodie, M. (2015). New Orleans Ten Years After The Storm: The Kaiser Family Foundation Katrina Survey Project. Retrieved from: https://www.kff.org/other/report/new-orleans-ten-years-after-the-storm-the-kaiser-family-foundation-katrina-survey-project/	Incorrect outcome (does not focus on GBV)
59	Haskell, B. (2014). Sexuality and natural disaster: Challenges of LGBT communities facing Hurricane Katrina. SSRN Electronic Journal.	Does not contain primary data/analysis
60	Henke, A., & Hsu, L. C. (2020). The gender wage gap, weather, and intimate partner violence. Review of Economics of the Household, 18(2), 413-429.	Incorrect exposure (does not focus on extreme events, but overall temperature)

61	Horton, L. (2015). Disaster through a gender lens: A case study from Haiti. In Disaster Research (pp. 154-170). Routledge.	Does not contain primary data/analysis
62	Houghton, R. (2009). Domestic violence reporting and disasters in New Zealand. Regional Development Dialogue, 30(1), 79-90.	Full text inaccessible/unavailable
63	Houghton, R. (2009). Everything became a struggle, absolute struggle': post-flood increases in domestic violence in New Zealand. Women, gender and disaster: Global issues and initiatives, 99-111.	Does not contain primary data/analysis
64	Hyder, Tina, and Johanna Mac Veigh. (2007). "Gender-Based Violence against Children in Emergencies: Save the Children UK's Response." Gender and Development 15 (1): 81–93.	Incorrect exposure (does not focus on extreme events, but emergency situations more broadly)
65	Ikeda, K (1995) Gender differences in human loss and vulnerability in natural disasters: a case study from Bangladesh. Indian Journal of Gender Studies 2(2), 171–193	Incorrect outcome (does not focus on GBV, but general gender differences)
66	International Planned Parenthood Federation (2021). The climate crisis and sexual and reproductive health and rights. Retrieved from: https://www.ippf.org/sites/default/files/2021-03/IPPF%20position%20paper%20The%20climate%20crisis%20and%20sexual%20and%20reproductive%20health%20and%20rights_Jan2021.pdf	Does not contain primary data/analysis
67	James, K., Breckenridge, J., Braaf, R., & Barrett Meyering, I. (2014). Responding to domestic violence in the wake of disasters: exploring the workers' perceptions of the effects of cyclone Yasi on women. In Issues of Gender and Sexual Orientation in Humanitarian Emergencies (pp. 113-124). Springer, Cham.	Incorrect outcome (does not focus on GBV but on the ability of agencies to respond to GBV)
68	Jansen, H. A., Nguyen, T. V. N., & Hoang, T. A. (2016). Exploring risk factors associated with intimate partner violence in Vietnam: results from a cross-sectional national survey. International journal of public health, 61(8), 923-934.	Incorrect exposure (does not focus on extreme events)
69	Joshi, P. C. (2012). Contextualising domestic violence in post-disaster situation: qualitative and quantitative analysis. South Asian Anthropologist, 12(1), 37-43.	Full text inaccessible/unavailable
70	Joshi, P. C. (2012). Disaster and domestic violence: the alcohol connection. Man in India, 92(3-4), 551-559	Full text inaccessible/unavailable
71	Klomp, J., & Bulte, E. (2013). Climate change, weather shocks, and violent conflict: A critical look at the evidence. Agricultural Economics, 44(s1), 63-78.	Does not contain primary data/analysis
72	Knight, K., & Welton-Mitchell, C. (2013). Gender identity and disaster response in Nepal. Forced Migration Review, (42), 57.	Does not contain primary data/analysis
73	Kumala Dewi, L. P. R., & Dartanto, T. (2019). Natural disasters and girls vulnerability: is child marriage a coping strategy of economic shocks in Indonesia?. Vulnerable children and youth studies, 14(1), 24-35.	Incorrect outcome (does not focus on GBV)
74	L. Juran, J. Trivedi Women, gender norms, and natural disasters in Bangladesh Geogr. Rev., 105 (2015), pp. 601-611	Does not contain primary data/analysis
75	Lai, B. S., Osborne, M. C., Lee, N., Self-Brown, S., Esnard, A. M., & Kelley, M. L. (2018). Trauma-informed schools: Child disaster exposure, community violence and somatic symptoms. Journal of affective disorders, 238, 586-592.	Incorrect outcome (does not focus on GBV, but on community violence without disaggregating by gender or sex)
76	Lammiman, C. (2019). The gender dimensions of the 2013 Southern Alberta floods. In Emerging Voices in Natural Hazards Research (pp. 27-55). Butterworth-Heinemann.	Incorrect outcome (does not focus on GBV, but broader gender dimensions)

77	Larkin, B. (2019). Pride and prejudice: LGBTIQ community responses to disaster events worldwide. Australian Journal of Emergency Management, The, 34(4), 60-66.	Does not contain primary data/analysis
78	Leap, W. L., Lewin, E., & Wilson, N. (2007). Queering the disaster: a presidential session. North American Dialogue, 10(2), 11-14.	Does not contain primary data/analysis
79	Lebeau, J.L., 1994. The oscillation of police calls to domestic disputes with time and the temperature humidity index. J. Crime Justice	Incorrect exposure (not extreme event, but temperature humidity index)
80	Luetke, M., Judge, A., Kianersi, S., Jules, R., & Rosenberg, M. (2020). Hurricane impact associated with transactional sex and moderated, but not mediated, by economic factors in Okay, Haiti. Social Science & Medicine, 261, 113189.	Incorrect outcome (does not focus on GBV but transactional sex)
81	Madkour, A. S., Johnson, C. C., Clum, G. A., & Brown, L. (2011). Disaster and youth violence: the experience of school-attending youth in New Orleans. Journal of Adolescent Health, 49(2), 213-215.	Incorrect outcome (not on GBV, youth violence without disaggregation by gender or sex)
82	McGinn, T., Bhabha, J., Garfield, R., Johnson, K., Luchsinger, G., Oddy, L., ... & Searle, L. (2015). Shelter from the storm: a transformative agenda for women and girls in a crisis-prone world. Columbia: United Nations Population Fund.	Does not contain primary data/analysis
83	McKinnon, S., Gorman-Murray, A., & Dominey-Howes, D. (2017). Disasters, queer narratives, and the news: how are LGBTI disaster experiences reported by the mainstream and LGBTI media?. Journal of homosexuality, 64(1), 122-144.	Incorrect outcome (not GBV, focus on media reporting on the experience of lesbian, gay, bisexual, transgender, and intersex people)
84	McLean, I. (2007). Climatic effects on incidence of sexual assault. Journal of Forensic and Legal Medicine, 14(1), 16-19.	Incorrect exposure (no extreme event, but gender weather conditions including temperature and rainfall)
85	Mian, L.H., Namasivayam, M. (2017). Sex, rights, gender in the age of climate change. Kuala Lumpur: Asian-Pacific Resource & Research Centre for Women	Does not contain primary data/analysis
86	Michael, R.P., Zumpe, D., 1986. An annual rhythm in the battering of women. Am. J. Psychiatr.	Incorrect exposure (not extreme event, but ambient temperature)
87	Moreno-Walton, L., Avegno, J., & Lee, B. (2012). The Effect of Hurricane Katrina on Violence against Women in Orleans Parish. Journal of Emergency Medicine, 43(5), 920.	Does not contain primary data/analysis
88	National Sexual Violence Resource Center. (2021). Sexual Violence in Disasters. Retrieved from: https://www.nsvrc.org/sites/default/files/2021-11/sexual_violence_in_disasters_final508_0.pdf	Does not contain primary data/analysis
89	Madkour, A. S., Johnson, C. C., Clum, G. A., & Brown, L. (2011). Disaster and youth violence: the experience of school-attending youth in New Orleans. Journal of Adolescent Health, 49(2), 213-215.	Incorrect outcome (not on GBV, youth violence without disaggregation by gender or sex)
90	Nelson, V., Meadows, K., Cannon, T., Morton, J., & Martin, A. (2002). Uncertain predictions, invisible impacts, and the need to mainstream gender in climate change adaptations. Gender & Development, 10(2), 51-59.	Incorrect outcome (not on GBV, but broader gendered impacts)

91	Norwegian Red Cross. "That nevers happens here" Sexual and gender based violence against men, boys, and including LGTBIQ+ persons in humanitarian settings. Retrieved from: https://www.rodekors.no/globalassets/_rapporter/thatneverhappenshere_uu.pdf?mc_phishing_protection_id=28048-c7t8vm70s0vafoouhkkg	Does not contain primary data/analysis
92	Ozawa, K. (2012). Relief activities for LGBTI people in the affected areas. Voices from Japan, 26, 21-22.	Full text inaccessible/unavailable
93	Pasten, R., Figueroa, E., & Colther, C. (2020). Not a Dream Wedding: The Hidden Nexus Between Climate Change and Child Marriage (No. wp508).	Incorrect exposure (not extreme event, but climate change broadly)
94	Petchesky, R. P. (2016). Biopolitics at the crossroads of sexuality and disaster: The case of Haiti. In The Ashgate Research Companion to the Globalization of Health (pp. 191-212). Routledge.	Does not contain primary data/analysis
95	Picardo, C. W., Burton, S. V., & Naponick, J. (2007, April). Gender-based violence experiences in reproductive-aged women displaced by Hurricane Katrina. In Obstetrics and Gynecology (Vol. 109, No. 4, pp. 63S-63S).	Full text inaccessible/unavailable
96	Pincha C & Krishna H (2008). Aravanis: voiceless victims of the tsunami, humanitarian Exchange Magazine, no. 41.	Incorrect outcome (not GBV, not on violence against Aravanis)
97	Popova, O., Otrachshenko, V., & Tavares, J. (2019). Extreme temperature and extreme violence across age and gender: Evidence from Russia (No. 382). GLO Discussion Paper.	Incorrect outcome (does not focus on GBV, but general violence)
98	Rahman, M (2013) Climate change, disaster and gender vulnerability: a study on two divisions of Bangladesh. American Journal of Human Ecology 2(2), 72–82	Does not contain primary data/analysis
99	Ranson, Matthew (2014) Crime, weather, and climate change. Journal of Environmental Economics and Management	Incorrect outcome (not GBV, violence not disaggregated by gender or sex)
100	Rao, N., Lawson, E. T., Raditloaneng, W. N., Solomon, D., & Angula, M. N. (2019). Gendered vulnerabilities to climate change: insights from the semi-arid regions of Africa and Asia. Climate and Development, 11(1), 14-26.	Incorrect outcome (not on GBV, but general gendered vulnerabilities)
101	Rees, S., & Wells, R. (2020). Bushfires, COVID-19 and the urgent need for an Australian Task Force on gender, mental health and disaster. Australian & New Zealand Journal of Psychiatry, 54(11), 1135-1136.	Does not contain primary data/analysis
102	Richards, G. (2010). Queering Katrina: gay discourses of the disaster in New Orleans. Journal of American Studies, 44(3), 519-534.	Incorrect outcome (not GBV, not on violence against queer people)
103	Rotton, J., & Cohn, E. G. (2001). Temperature, routine activities, and domestic violence: A reanalysis.	Does not contain primary data/analysis
104	Rydstrom, H. (2019). Crises, ruination and slow harm: Masculinised livelihoods and gendered ramifications of storms in Vietnam. In Climate hazards, disasters, and gender ramifications (pp. 213-229). Routledge.	Full text inaccessible/unavailable
105	Rydström, Helle. (2020) "Disasters, ruins, and crises: Masculinity and ramifications of storms in Vietnam." Ethnos 85.2: 351-370.	Does not contain primary data/analysis on extreme events and GBV
106	Schutte, F. H., & Breetzke, G. D. (2018). The influence of extreme weather conditions on the magnitude and spatial distribution of crime in Tshwane (2001–2006). South African Geographical Journal Suid-Afrikaanse Geografiese Tydskrif, 100(3), 364-377.	Does not contain primary data/analysis

107	South East Healthy Communities Partnership. (2012). The Relationship Between Climate Change and Violence: A Literature Review. Retrieved from: https://enliven.org.au/wp-content/uploads/2018/06/SEHCP-Climate-Change-and-Violence-Report-2012.pdf	Does not contain primary data/analysis
108	Straight, B., Hilton, C. E., Onicescu, G., Needham, B., Naugle, A., Owuor, O. C., ... & Kelempu, C. S. (2019). Climate Change and Invisible Suffering: Transgenerational Impacts of Traumatic Maternal Experiences of Extreme Drought.	Incorrect outcome (does not focus on GBV, but other traumatic maternal experiences)
109	Thuringer, C. (2016). Left out and behind: Fully incorporating gender into the climate discourse. New Security Beat. Retrieved from: https://www.newsecuritybeat.org/2016/08/left-behind-fully-incorporating-gender-climate-discourse/	Does not contain primary data/analysis
110	Trinh, T. A., & Zhang, Q. (2021). Adverse shocks, household expenditure and child marriage: evidence from India and Vietnam. Empirical economics, 61(3), 1617-1639.	Does not contain primary data/analysis
111	True, J. (2013). Gendered violence in natural disasters: Learning from New Orleans, Haiti and Christchurch. Aotearoa New Zealand Social Work.	Incorrect exposure (not extreme event)
112	UN Women. Climate Change, Disasters and Gender-based Violence in the Pacific. Retrieved from: https://www.uncclearn.org/wp-content/uploads/library/unwomen701.pdf	Does not contain primary data/analysis
113	UNISDR. Background paper: issues of vulnerability with specific reference to gender in the Asia Pacific - post-2015 framework for disaster risk reduction consultations. Retrieved from: https://www.preventionweb.net/files/34051_backgroundpaperonissuesofvulnerabil.pdf	Does not contain primary data/analysis
114	Valerio, K. A. (2014). Storm of violence, surge of struggle: Women in the aftermath of Typhoon Haiyan (Yolanda). Asian Journal of Women's Studies, 20(1), 148-163.	Does not contain primary data/analysis
115	Vinyeta, K., Whyte, K., & Lynn, K. (2016). Climate change through an intersectional lens: gendered vulnerability and resilience in indigenous communities in the United States. United States Department of Agriculture. Retrieved from: https://www.fs.fed.us/pnw/pubs/pnw_gtr923.pdf	Does not contain primary data/analysis
116	Wachholz, S. (2013). 'At risk': Climate change and its bearing on women's vulnerability to male violence. In Issues in green criminology (pp. 183-207). Willan.	Does not contain primary data/analysis
117	Whittenbury, K. (2013). Climate change, women's health, wellbeing and experiences of gender based violence in Australia. In Research, action and policy: Addressing the gendered impacts of climate change (pp. 207-221). Springer, Dordrecht.	Does not contain primary data/analysis
118	WHO (World Health Organization). (2009). Gender, Climate Change and Health: Draft Discussion Paper. Retrieved from: https://www.who.int/globalchange/publications/reports/final_who_gender.pdf	Does not contain primary data/analysis
119	Wilson, J., Phillips, B., & Neal, D. M. (1998). Domestic violence after disaster. The gendered terrain of disaster, 115-123.	Full text inaccessible/unavailable
120	Women Deliver. (2021). The Link Between Climate Change and Sexual and Reproductive Health and Rights. Retrieved from: https://womendeliver.org/wp-content/uploads/2021/02/Climate-Change-Report.pdf	Does not contain primary data/analysis
121	World Health Organization. (2014). Gender, climate change and health. World Health Organization. Retrieved from: https://apps.who.int/iris/bitstream/handle/10665/144781/9789241508186_eng.pdf	Does not contain primary data/analysis
122	Xu, R., Xiong, X., Abramson, M. J., Li, S., & Guo, Y. (2021). Association between ambient temperature and sex offense: A case-crossover study in seven large US cities, 2007–2017. Sustainable Cities and Society, 69, 102828	Incorrect exposure (not extreme event, but ambient temperature)

123	Yamashita, A. (2012). Beyond Invisibility: Great East Japan Disaster and LGBT in Northeast Japan. FOCUS, 69.	Does not contain primary data/analysis
124	Yamashita, A., Gomez, C., & Dombroski, K. (2017). Segregation, exclusion and LGBT people in disaster impacted areas: experiences from the Higashinihon Dai-Shinsai (Great East-Japan Disaster). Gender, Place & Culture, 24(1), 64-71.	Does not contain primary data/analysis
125	Report on Database of Sexual Violence Prevalence and Incidence Related to Hurricanes Katrina and Rita (2006). Retrieved from: https://www.nsvrc.org/sites/default/files/Publications_NSVRC_Reports_Report-on-Database-of-Sexual-Violence-Prevalence-and-Incidence-Related-to-Hurricane-Katrina-and-Rita.pdf	Incorrect outcome (does not focus on GBV, reports on domestic violence without disaggregating by gender or sex)

Supplementary Table 8. Excluded articles on gender-based violence and earthquakes and tsunamis retrieved in the search process (n=26)

Study	Study design	Study Period	Country	Type of extreme event	Type or definition of GBV	Population source	Results/Conclusion
**Peer-reviewed studies**							
1. Bradley *et al.* 2021[44]	Mixed-methods study<br><br>Quantitative using cross-sectional survey<br><br>Qualitative using in-depth interviews and stakeholder interviews	2018-2019	Nepal	Earthquake<br>(2015 Nepal earthquake)	Any act of GBV that results in or is likely to result in physical, sexual, or psychological harm or suffering to women and/or girls<br>*Perpetrator:* spouse, ex-spouse, family member, stranger, known person	Women and men living in Kathmandu and Morang Districts impacted by the 2017 floods and/or 2015 earthquake.<br><br>*N participants:* 880 survey respondents, 53 in-depth interviews, 20 stakeholder interviews<br>*Age:* 18-49 yrs	Of female respondents, 36% reported experiencing IPV personally. Of 318 respondents reporting experiencing or perpetrating IPV, 35% report it occurring in the past 0-11 months. This suggests an increase from pre-2015 norms in comparison to Nepalese DHS (2016) statistics of 7% of women experiencing IPV ever and 3% in the last year.
2. Campbell *et al.* 2016[45]	Quantitative, cross-sectional study using a computer-assisted self-interview device	2011-2013	Haiti	Earthquake<br>(2010 Haitian earthquake)	Intimate partner violence (physical and /or sexual assaults)<br>*Perpetrator:* former or current intimate partner	Haitian national women that attended local hospitals or clinics<br><br>*N participants:* 208<br>*Age:* 18-44 yrs	Abuse and violence pre-disaster (71.22%) and post-disaster (75%) was high, p=0.266.
3. Cerna-Turoff et *al.* 2020[46]	Quantitative, cross-sectional study using a survey	2012	Haiti	Earthquake<br>(2010 Haitian earthquake)	Physical, emotional and sexual violence<br>*Perpetrator:* parent, caregiver, adult relative, authority figure, anyone in the community	Girls that were part of the Haiti Violence Against Children Survey<br><br>*N participants:* 635<br>*Age:* 13-17 yrs	The odds ratio of any form of violence against girl after displacement due to the earthquake: 0.84 (95 % CI: 0.52–1.33, p = 0.500)
4. Chan *et al.* 2011[47]	Quantitative, cross-sectional study using a survey	2008	China	Earthquake<br>(2008 Sichuan earthquake)	Phycological aggression and physical violence<br>*Perpetrator:* spouse, ex-spouse, family member, stranger, known person	Chinese women in temporary shelters for survivors that were married, cohabiting or had a child.<br><br>*N participants:* 186<br>*Age:* >18 yrs old	After the earthquake, all types of family violence increased.<br>*Psychological aggression (all)*<br>pre-earthquake prevalence: 10.5%<br>post-earthquake prevalence: 19.3%<br>*Physical violence (all)*<br>pre-earthquake prevalence: 5.0%<br>post-earthquake prevalence: 6.6%
5. Irshad *et al.* 2012[48]	Qualitative study, using an ethnographic approach, in-depth interviews, group discussions, and observation	2008	Pakistan	Earthquake<br>(2005 Pakistan Earthquake)	Abandonment, child marriage, violence, abuse<br>*Perpetrator:* Husband	Women and men rendered paraplegic by spinal cord injuries from the earthquake.<br><br>*N participants:* 73<br>*Age:* >16 yrs	A majority of female respondents' husbands had remarried or were keen to do so because of their wives' disability post-earthquake. A large number of them married girls as young as 13 which appeared connected to a deliberate strategy of ensuring child-bride could be manipulated and controlled. For some women receiving disability stipends contributed to distrust and violence or abuse from their husbands. There was an increase in marrying off girls (as young as

							13) to older men as replacements for their disabled wives or as social protection due to the death of a parent in the earthquake.
6. Fisher *et al.* 2010[49]	Qualitative study, using semi-structured interviews	2004	Sri Lanka	Tsunami (2004 Sri Lanka Tsunami)	Rape, physical abuse, sexual abuse, domestic violence, harassment, murder, sexual exploitation & manipulation, child marriage<br>*Perpetrator:* Strangers, spouses, family members, authority figures, relief providers	Representatives of community and nongovernmental organisations (NGOs), international NGOs and UN agencies, government<br><br>*N participants:* 60<br>*Age:*	Domestic violence was considered the most prevalent & sustained form of post-disaster violence against women by 4/5 respondents. 2/3 of respondents thought relationships had become more violent. There was an increase in marriages of young women and girls to replace deceased wives or lessen the family financial burden.
7. Kolbe *et al.* 2010[50]	Quantitative study, cross-sectional using a survey	2009-2010	Haiti	Earthquake (2010 Haitian earthquake)	Sexual violence<br>*Perpetrator:* Spouse, boyfriend, ex-partner, neighbours, friend or former friend, criminals	People participating in a 2009 pre-earthquake and 2010 post-earthquake survey administered in the Port-au-Prince area.<br><br>*N participants:* 1,732<br>*Age:* mean 24 yrs, median 22 yrs	Estimation that 5,280 women (0.8%; 95% CI 0.5–1.4) and 5,209 girls < 18 (0.7%; 95% CI 0.4–1.1) were sexually assaulted after the earthquake. This included rape with penetration, unwanted sexual touching, oral sex and forced witnessing of sexual acts.
8. Lai *et al.* 2020[51]	Quantitative study, cross-sectional household survey	2012	Haiti	Earthquake (2010 Haitian earthquake)	Physical, emotional, and sexual violence<br>*Perpetrator:* Parents, family, adult caregivers, authority figures,	Youth surveyed in Haiti Violence against Children Survey (VACS).<br><br>*N participants:* 1457 females, 1459 males<br>*Age:* 13-24 yrs	High rates of violence were reported by youth post-earthquake. Females reported in the past year: any violence victimization (49.94%); parent/adult caregiver physical violence (22.03%); public authority figure physical violence (10.20%); emotional abuse (27.80%); & sexual violence (23.01%).
9. Logie *et al.* 2016[52]	Qualitative study, using focus groups and in-depth interviews	Not reported	Haiti	Earthquake (2010 Haitian earthquake)	IPV, survival sex work, gender-based sexual violence<br>*Perpetrator:* Strangers, partners	Youth living in Leogane and identifying as internally displaced due to the 2010 earthquake<br><br>*N participants:* 60 (30 females, 30 males)<br>*Age:* 18-24 yrs	Women reported sexual violence was commonplace and viewed insecure housing conditions (tent cities especially) as unsafe. Women reported GBSV occurring in connection with other poverty-related crimes. Poverty contributed to women's engagement in survival sex work. Poverty also reduced their power in relationships and increased their vulnerability to IPV.
10. Rao *et al.* 2020[53]	Quantitative study, analysis of National Family Health Surveys part of DHS	1998-99, 2005-06, 2015-16	India	Tsunami (2004 Indian Ocean Tsunami)	IPV (physical, sexual, and emotional violence)<br>*Perpetrator:* Spouse or intimate partner. Other perpetrators were also included in the Wave 1 survey.	Women (1998-99) & ever-married women (2005-06, 2015-16) who responded yes to the NFHS survey question of whether they experienced physical violence in the last 12 months.<br><br>*N participants:*<br>*Age:* 15-49 yrs	In Wave 1 (1999), 1772 (45.68%) women reported IPV; in Wave 2 (2006), 2850 (21.22%) women reported IPV; and in Wave 3 (2016) 2432 (31.31%) women reported IPV. Wave 1 survey included violence from non-intimate partners but subsequent surveys did not which could explain the drop. When controlled for the state of residence, the tsunami was

							associated with a higher risk of IPV in 3 or 4 states: women had 98% higher odds of experiencing IPV (p < 0.001) in TamilNadu, 85% higher (p<0.001) in Andhra Pradesh, and 41% (p<0.05) in Kerala.
11. Rahill *et al.* 2015[54]	Qualitative study, using focus groups		Haiti	Earthquake (2010 Haitian earthquake)	Non-consensual sex, enforced with purposeful injury, strangulation, & aim to "crush the uterus" *Perpetrator:* multiple, unknown non-intimate partners/perpetrators	Women residents of Cité Soleil who survived the earthquake and its aftershocks (along with hurricanes and cholera) *N participants:* 2 focus groups of 16 women *Age:* 19-52 yrs	All participants in the focus groups experienced sexual violence highlighting purposeful injury (including the use of broken marbles, rubber bands and other objects).
12. Rees *et al.* 2005[55]	Qualitative study, using focus groups and storyboard methodology	2005	Sri Lanka	Tsunami	Sexual and gender based violence, trafficking *Perpetrator:* Strangers, intimate-partners	Community worker women in Colombo and an additional regional location *N participants:* 88 women *Age:* Not reported	Participants reported an increased vulnerability to GBV
13. Sakurai *et al.* 2017[56]	Quantitative study, using data from cohort epidemiological survey JECS	2011	Japan	Earthquake (2011 Great East Japan Disaster)	Domestic violence (mental/emotional & physical) *Perpetrator:* Intimate partners	Pregnant females in Miyagi Prefecture *N participants:* 7600 *Age:*	The JECS did not compare to DV rates prior to the earthquake. No significant difference was found in DV incidence during the 3- to 9- month study following the earthquake. The incidence of physical DV was higher in the north coastal area (5.9%) than nationwide (1.5%, $P<0.0001$) or in the inland (1.3%, $P=0.0007$) area.
14. Sloand *et al.* 2017[57]	Qualitative study, using descriptive/correlational, focus groups, interviews, and situational analysis methodology	2011-2013	Haiti	Earthquake (2010 Haitian earthquake)	Physical, psychological, and sexual violence *Perpetrator:* boyfriend, ex-boyfriend, other partners, family members, non-family members, authority figures, or other individuals	Internally displaced adolescent girls living in Port Au Prince tent camps *N participants:* 78 *Age:* 12-17 yrs	There was no statistically reported increase in sexual abuse pre- and post-earthquake, but 61% of the girls did not respond to questions about abuse either before or after the earthquake. However, the odds of an adolescent Haitian girl being sexually abused increased significantly post-earthquake and 30% of girls reported a family member as the perpetrator of violence post-earthquake.
15.Sohrabizadeh *et al.* 2016[58]	Qualitative study, using in-depth structured interviews and field observations	2014	Iran	Earthquakes and floods	Domestic violence (physical, psychological, sexual), community violence (psychological violence & sexual harassment), early/forced marriages *Perpetrator:* husbands, sons, fathers, other male family members	Affected women and key informants *N participants:* 15 (8 affected women) *Age:* 17-55 yrs	Participants reported experiencing physical abuse, psychological violence, early/forced marriages, and sexual violence within their relationships following the disaster. In the community, they experienced increased psychological violence & sexual harassment.
16.Sohrabizadeh *et al.* 2017[59]	Qualitative study, using in-depth unstructured interviews	NR	Iran	Twin Earthquakes	Early/forced marriages, restricted access to education, increased control	Affected people living in destroyed regions of easter Azerbaijan	The twin earthquakes exacerbated the violence faced by women.

					by men, domestic violence<br>*Perpetrator:* father, husband, brother, uncle	*N participants:* 13 (11 women, 2 men)<br>*Age:* 17-60 yrs	
17. Standing *et al.* 2016[60]	Qualitative study, using social media and discussions with key informants	NR	Nepal	Earthquake<br>(2015 Nepal earthquake)	Violence against women and girls, trafficking, rape, mental torture, abuse, harassment<br>*Perpetrator:*	*N/A*<br><br>*N participants:*<br>*Age:*	Following the earthquake, there was an increase in trafficking and violence, especially if girls were separated from their families.
18. Subedi *et al.* 2020[61]	Qualitative study, using cross-sectional analysis of DHS data	2012	Haiti	Earthquake<br>(2010 Haitian earthquake)	Child abuse<br>*Perpetrator: parents or family members*	Sampled households with at least one child<br><br>*N participants:* 8351 households<br>*Age:* 2-14 yrs	The relative risk for severe physical abuse associated with earthquake-related damage to the home was notably higher for older females (RR=0.84, 95% CI: 0.97-1.42), although not statistically significant.
19. Tanoue *et al.* 2019[62]	Quantitative study, using questionnaires	2011-2014	Japan	Earthquake<br>(2011 Great East Japan Disaster)	Intimate partner violence<br>*Perpetrator:* intimated partner	Pregnant women in the 2nd or 3rd trimester who responded to JECS questionnaire<br><br>*N participants:* 79,222<br>*Age:* <25->35 yrs	Reports of mental IPV experienced during pregnancy significantly decreased after peaking following the disaster in the southern coastal region (19.4% in FY 2011, 13.1% in FY 2012, 13.3% in FY 2013, p = 0.05, trend p = 0.04). There was no significant difference in the north coastal area. For physical IPV, there was no significant difference in the south and in the north coastal region, it significantly decreased after peaking post-earthquake (2.7%, 1.5%, 1.3%; p = 0.08, trend p = 0.03). Mental and physical IPV prevalence was higher every year in the inland regions.
20. Tearne *et al.* 2021[63]	Qualitative study, using semi-structured key informant interviews and focus group discussions	2016	Nepal	Earthquake<br>(2015 Nepal earthquake)	Abduction; trafficking; domestic, physical, & sexual violence<br>*Perpetrator:*	Adolescents & adults from three disaster-affected areas of Nepal<br><br>*N participants:* 62 (35 adolescents, 48.6% female; 27 adults, 55.6% female)<br>*Age:* 13-19; 23-58 yrs	Participants emphasised that the earthquake and extreme stress exacerbated pre-existing problems related to domestic & sexual violence. Inadequate safety and consideration of gender were frequently cited as risk factors for violence.
21. Yoshihama *et al.* 2018[64]	Quantitative study, cross-sectional using surveys	2011-2012	Japan	Earthquake<br>(2011 Great East Japan Disaster)	Domestic violence (physical, verbal, emotional, sexual, financial, neglect), sexual assault, sexual harassment<br>*Perpetrator:* intimate partner (45 cases), other (26 cases; all but one were known to victim i.e. family, neighbour, residents of the evacuation centre, co-worker, etc.)	Professionals, practitioners, NGO members, volunteers, and others involved in disaster response who were in a position to witness/hear about incidents of violence against women & children<br><br>*N participants:* 82<br>*Age:* <5 to >70 yrs	Regarding DV or IPV, a notable increase in frequency or severity was reported after the disaster and very few cases reported new violence post-disaster. Around 2/3 of non-IPV incidents took place in a locale to which victims had been evacuated due to the disaster. Quid pro quo assaults related to relief distribution were reported frequently. 11 cases reported violence against minors: 5 by strangers, 10 taking place at relocation space.

22. Weitzman *et al.* 2016[65]	Quantitative study, cross-sectional using surveys	Unexposed: 2005, 2006<br>Exposed: 2012	Haiti	Earthquake<br>(2010 Haitian earthquake)	IPV (physical & sexual violence)<br>*Perpetrator:* intimate partners	Respondents of DHS survey (1 random woman per household) who were not widowed at the time of the survey<br><br>*N participants:* 2,535 unexposed; 6,287 exposed<br>*Age:* 15-49 yrs	Physical IPV rose (0.4% probability increase in any violence) in regions devastated by the earthquake and fell (2% decrease in the probability that violence occurred 'sometimes')in moderately impacted regions. Sexual IPV rates fell everywhere but at a rate 300% greater in the moderately impacted regions. Probabilities of IPV were higher among women living in a displacement camp, who lost a household member, or who were in a household impacted by the cholera outbreak
**Grey literature & not peer-reviewed work**							
23.GLHRC/SEROVie. 2011[66]	Qualitative research, using interviews	NR	Haiti	Earthquake (2010 Haitian earthquake)	Rape, sexual exploitation, sexual violence, corrective rape, harassment, physical abuse, hate crimes, discrimination, arbitrary arrests<br><br>*Perpetrator:* Family members, strangers, authority figures	LGBT Haitians accessing services from SEROvie NGO<br><br>NR	LGBT people are seen as gender non-conforming experienced an increased risk of gender-based violence in the wake of the disaster. Rape of lesbians, gay men, and transgender women; sexual violence and corrective rape in IDP camps; sexual exploitation, physical abuse, or denied access to emergency relief; abuse by police; and more were documented by SEROvie.
24. Human Rights Watch 2014[67]	Qualitative research, using interviews	2010-2011	Haiti	Earthquake (2010 Haitian earthquake)	Survival sex, sexual violence	Women & girls living in 15 displacement camps in 7/12 earthquake-affected communes in Port-au-Prince<br><br>*N participants:* 128<br><br>*Age:* >14 yrs	Food insecurity & poverty in the camps made women & girls vulnerable to the need to engage in survival sex. 6 of the interviewed pregnant women (3 of whom were 14-15 yrs) reported their pregnancies resulted from rapes. Conditions in the camp & reduced protection from social networks/community increased vulnerability to sexual violence.
25. SERVOvie 2010[68]	Qualitative research, using focus group discussion	2010	Haiti	Earthquake (2010 Haitian earthquake)	Medical neglect, harassment, denial of aid distribution, attacks, gang rape, arbitrary arrests, sexual violence<br><br>*Perpetrator:* strangers, authority figures	LGBT youth & sex workers in Port-au-Prince<br><br>*N participants:* 30	Participants described medical discrimination; harassment, violence, & abuse in the camps; being denied resources; being blamed for the earthquake; being attacked on the street by community members and police; arbitrary arrests; gang rapes; and other forms of GBV in connection with their sexuality and/or gender presentation.
26. Fontanez *et al.* 2019F	Qualitative research, using in-depth interviews	2019	United States	Natural Disaster (2018)<br><br>Not further specified	Direct and non-direct harassment of transgender people	Displaced, transgender individuals that stayed in shelters that were recruited using crowdsourcing (e.g. social media outlets	Transgender people experienced direct and non-direct harassment in shelters.<br>They tend to stay away from shelters

	PhD thesis (unpublished)	*Perpetrator:* other displaced people staying at the shelters.	for recruitment)<br><br>*N participants:* 12<br>*Age range:* 23-61 yrs	because of fear of assault or discrimination.

Supplementary Table 9. Critical appraisal of included cross-sectional and ecological studies (n=25) using the New Castle Ottawa Scale.

Study	Selection (max 5 stars)				Comparability (max 2 stars)	Outcomes (max 3 stars)		Total	
	Representativeness of the sample	*Sample size*	*Non-respondents*	*Ascertainment of the exposure (risk factor)*	*The subjects in different outcome groups are comparable, based on the study design or analysis. Confounding factors are controlled.*	*Assessment of the outcome*	*Statistical test*	*Total*	*AHRQ standard quality*
Ahmed *et al.* 2019[42*]	★		★	★		★		4	Poor
Allen *et al.* 2021[11]	★	★	★	★ ★	★ ★	★	★	9	Good
Anastario *et al.* 2009[3]	★			★	★	★	★	5	Fair
Asadullah *et al.* 2020[39*]				★		★		2	Poor
Azad *et al.* 2013[40*]				★		★		2	Poor
Carrico *et al.* 2020[20]	★			★★	★★	★	★	7	Good
Cools *et al.* 2020[14]	★	★		★★	★★	★	★	8	Good
Cooper *et al.* 2021[16]	★	★	★	★ ★	★ ★	★	★	9	Good
Corno *et al.* 2020[17]	★	★	★	★ ★	★ ★	★	★	9	Good
Díaz & Saldarriage 2020[12]	★	★	★	★ ★	★ ★	★	★	9	Good
Epstein *et al.* 2020[15]	★	★		★★	★★	★	★	7	Good
Fagen *et al.* 2011[5]						★		1	Poor
Frasier *et al.* 2004[10]	★		★	★★	★	★		6	Poor
Harville *et al.* 2011[6]			★	★★	★	★	★	5	Fair
Houghton *et al.* 2010[38*]						★★		2	Poor

Memon *et al.* 2020[41*]	★					★		2	Poor
Miguel *et al.* 2005[13]	★	★		★★	★★	★★	★	9	Good
Molyneaux *et al.* 2020[22]	★	★		★★	★	★	★	7	Fair
Picardo *et al.* 2010[4]	★			★	★	★	★	5	Fair
Rai *et al.* 2020[19]	★	★	★		★★	★	★	7	Fair
Sanz-Barbero *et al.* 2018[21]	★			★	★	★	★	5	Fair
Schumacher *et al.* 2010[7]	★		★	★	★★	★	★	7	Good
Sekhri & Storeygard 2011[18]	★			★	★	★	★	5	Fair
Temple *et al.* 2011[8]	★				★★	★		4	Poor
Westhoff *et al.* 2008[9]	★			★		★		3	Poor

[*] These studies have mixed-methods and are assessed in quality for their quantitative and qualitative part separately.

Each ★ represents a point for a given quality indicator.
AHRQ, Agency for Healthcare Research and Quality; quality categorized as good, fair or poor.

Thresholds for converting the Newcastle-Ottawa scales to AHRQ standards (good, fair, and poor):
Good quality: 3 or 4 stars in selection domain AND 1 or 2 stars in comparability domain AND 2 or 3 stars in outcome/exposure domain
Fair quality: 2 stars in selection domain AND 1 or 2 stars in comparability domain AND 2 or 3 stars in outcome/exposure domain
Poor quality: 0 or 1 star in selection domain OR 0 stars in comparability domain OR 0 or 1 stars in outcome/exposure domain

Supplementary Table 10. Critical appraisal of included qualitative studies (n=19) using the Critical Appraisal Skills Programme (CASP) tool.[69]

Study	Section A: Are the results valid?						Section B: What are the results?		
	Was there a clear statement of the aims of the research?	Is a qualitative methodology appropriate?	Was the research design appropriate to address the aims of the research?	Was the recruitment strategy appropriate to the aims of the research?	Was the data collected in a way that addressed the research issue?	Has the relationship between the researcher and participants been adequately considered?	Have ethical issues been taken into consideration?	Was the data analysis sufficiently rigorous?	Is there a clear statement of findings?
Ahmed *et al.* 2019[42*]	Yes	Yes	Yes	No	Can´t Tell	No	Yes	Yes	Yes
Asadullah *et al.* 2020[39*]	Yes	Can´t Tell	No	No	Can´t Tell	Yes	Yes	No	Yes
Azad *et al.* 2013[40*]	No	Yes	Can´t Tell	Can´t Tell	Yes	No	No	Yes	Yes
Bermudez *et al.* 2019[23]	Yes	Yes	Yes	No	Yes	Can´t Tell	Yes	Can´t Tell	Yes
Esho *et al.* 2021[33]	Yes	Yes	Yes	Can't Tell	Can't Tell	No	No	Can't Tell	Yes
Forthergill 1999[32]	Yes	Yes	Yes	No	Can't Tell	No	No	Can't Tell	Yes
Hossen *et al.* 2021[34]	Yes	Yes	Yes	Can't Tell	Can't Tell	No	No	Yes	Yes
Houghton *et al.* 2010[38*]	Yes	Yes	Yes	Can´t Tell	Yes	No	Can´t Tell	Can´t Tell	Yes
Madhuri *et al.* 2016[29]	Yes	Yes	Yes	Yes	Yes	No	Yes	No	No
Memon *et al.* 2020[41*]	Yes	Yes	Yes	Yes	Yes	Yes	Yes	Can´t Tell	Yes
Nguyen *et al.* 2018[26]	Yes	Yes	Can´t Tell	Can´t Tell	Yes	Can´t Tell	Yes	Can´t Tell	Can´t Tell
Nguyen & Rydstrom 2018[27]	Yes	Yes	Yes	Can't Tell	Yes	No	No	Can't Tell	Yes

Parkinson & Zara 2013[35]	Yes	Yes	Yes	No	Can't Tell	No	Can't Tell	Yes	Yes
Parkinson *et al.* 2019[36]	Yes	Yes	Yes	Yes	Yes	Can´t Tell	Yes	Yes	Yes
Rashid *et al.* 2000[30]	Yes	Yes	Yes	Can´t Tell	Yes	Yes	Yes	No	No
Rezwana *et al.* 2020[25]	Yes	Yes	Yes	Yes	Yes	Yes	Yes	No	Yes
Singh *et al.* 2020[31]	Yes	Yes	Yes	Yes	Yes	Yes	Yes	Yes	Can´t Tell
Tanyag *et al.* 2018[28]	Yes	Yes	Yes	Yes	Yes	No	Yes	Can´t Tell	Yes
Thornston *et al.* 2007[24]	Yes	Yes	Can't Tell	No	Can't Tell	No	No	Yes	Yes

[*] These studies have mixed-methods and are assessed in quality for their quantitative and qualitative part separately.

Supplementary Table 11. Critical appraisal of included Grey Literature (n=2) using the Authority, Accuracy, Coverage, Objectivity, Date, Significance (AACODS) checklist.

#	Checklist question	IFRC[43]	Dwyer *et al.* 2018[37]
1	*Authority – identifying who is responsible for the intellectual content*	Can't tell	Yes
2	*Accuracy – is there a stated aim or brief, methodology, peer-review, reputable and representative*	Yes	Yes
3	*Coverage – are the limits clearly stated*	No	Yes
4	*Objectivity – is the authors' standpoint clear? Does the work seem balanced?*	Can't tell	Yes
5	*Date – is the item dated, relevant and contemporary*	Yes	Yes
6	*Significance – is the item meaningful; does it add context?*	Yes	Yes

References

1. Moher D, Liberati A, Tetzlaff J, Altman DG. Preferred reporting items for systematic reviews and meta-analyses: The PRISMA statement. *BMJ*. 2009;339(7716):332-336. doi:10.1136/bmj.b2535
2. Page MJ, McKenzie JE, Bossuyt PM, et al. The PRISMA 2020 statement: an updated guideline for reporting systematic reviews. *BMJ*. 2021;372. doi:10.1136/BMJ.N71
3. Anastario M, Shehab N, Lawry L. Increased Gender-based Violence Among Women Internally Displaced in Mississippi 2 Years Post–Hurricane Katrina. *Disaster Med Public Health Prep*. 2009;3(1):18-26. doi:10.1097/DMP.0B013E3181979C32
4. Picardo CW, Burton S, Naponick J, Katrina Reproductive Assessment Team. Physically and sexually violent experiences of reproductive-aged women displaced by Hurricane Katrina. *J La State Med Soc*. 2010;162(5):282, 284-288, 290. Accessed February 14, 2022. https://europepmc.org/article/med/21141260
5. JL F, W S, PB A. Why not the University of New Orleans? Social disorganization and sexual violence among internally displaced women of Hurricane Katrina. *J Community Health*. 2011;36(5):721-727. doi:10.1007/S10900-011-9365-7
6. EW H, CA T, H T, None XX, P B. Experience of Hurricane Katrina and reported intimate partner violence. *J Interpers Violence*. 2011;26(4):833-845. doi:10.1177/0886260510365861
7. JA S, SF C, FH N, M T, K C, S G. Intimate partner violence and Hurricane Katrina: predictors and associated mental health outcomes. *Violence Vict*. 2010;25(5):588-603. doi:10.1891/0886-6708.25.5.588
8. JR T, P van den B, JF T, J N, C T, DH F. Teen dating violence and substance use following a natural disaster: does evacuation status matter? *Am J Disaster Med*. 2011;6(4):201-206. doi:10.5055/AJDM.2011.0059
9. Westhoff WW, Lopez GE, Zapata LB, Corvin JAW, Allen P, Mcdermott RJ. Reproductive Health Education and Services Needs of Internally Displaced Persons and Refugees Following Disaster. *Am J Heal Educ*. 2008;39(2):95-103. doi:10.1080/19325037.2008.10599021
10. PY F, L B, E H, et al. Disaster down East: using participatory action research to explore intimate partner violence in eastern North Carolina. *Health Educ Behav*. 2004;31(4 Suppl). doi:10.1177/1090198104266035
11. Allen EM, Munala L, Henderson JR. Kenyan Women Bearing the Cost of Climate Change. *Int J Environ Res Public Heal 2021, Vol 18, Page 12697*. 2021;18(23):12697. doi:10.3390/IJERPH182312697
12. Díaz JJ, Saldarriaga V. A Drop of Love? Rainfall Shocks and Spousal Abuse: Evidence from Rural Peru. *SSRN Electron J*. Published online July 25, 2020. doi:10.2139/SSRN.3662976
13. Miguel E. Poverty and Witch Killing. *Rev Econ Stud*. 2005;72(4):1153-1172. doi:10.1111/0034-6527.00365
14. Cools S, Flatø M, Kotsadam A. Rainfall shocks and intimate partner violence in sub-Saharan Africa: *https://doi.org/101177/0022343319880252*. 2020;57(3):377-390. doi:10.1177/0022343319880252
15. Epstein A, Bendavid E, Nash D, Charlebois ED, Weiser SD. Drought and intimate partner violence towards women in 19 countries in sub-Saharan Africa during 2011-2018: A population-based study. *PLOS Med*. 2020;17(3):e1003064. doi:10.1371/JOURNAL.PMED.1003064
16. Cooper M, Sandler A, Vitellozzi S, et al. Re-examining the effects of drought on intimate-partner violence. *PLoS One*. 2021;16(7):e0254346. doi:10.1371/JOURNAL.PONE.0254346
17. Corno L, Hildebrandt N, Voena A. Age of Marriage, Weather Shocks, and the Direction of Marriage Payments. *Econometrica*. 2020;88(3):879-915. doi:10.3982/ECTA15505
18. Sekhri S, Storeygard A, Larson N, Gayle W-R, Stern S, Qian N. The Impact of Climate Variability on Crimes against Women: Dowry Deaths in India *.
19. Rai A, Sharma AJ, Subramanyam MA. Droughts, cyclones, and intimate partner violence: A disastrous mix for Indian women. *Int J Disaster Risk Reduct*. 2021;53:102023. doi:10.1016/J.IJDRR.2020.102023
20. Carrico AR, Donato KM, Best KB, Gilligan J. Extreme weather and marriage among girls and women in Bangladesh. *Glob Environ Chang*. 2020;65:102160.

doi:10.1016/J.GLOENVCHA.2020.102160
21. Sanz-Barbero B, Linares C, Vives-Cases C, González JL, López-Ossorio JJ, Díaz J. Heat wave and the risk of intimate partner violence. *Sci Total Environ*. 2018;644:413-419. doi:10.1016/J.SCITOTENV.2018.06.368
22. Molyneaux R, Gibbs L, Bryant RA, et al. Interpersonal violence and mental health outcomes following disaster. *BJPsych Open*. 2020;6(1). doi:10.1192/BJO.2019.82
23. Bermudez LG, Stark L, Bennouna C, et al. Converging drivers of interpersonal violence: Findings from a qualitative study in post-hurricane Haiti. *Child Abuse Negl*. 2019;89:178-191. doi:10.1016/J.CHIABU.2019.01.003
24. Thornton WE, Voigt L. Disaster Rape: Vulnerability of Women to Sexual Assaults During Hurricane Katrina. :23.
25. Rezwana N, Pain R. Gender-based violence before, during and after cyclones: slow violence and layered disasters. *Disasters*. Published online 2020. doi:10.1111/DISA.12441
26. Nguyen HT. Gendered Vulnerabilities in Times of Natural Disasters: Male-to-Female Violence in the Philippines in the Aftermath of Super Typhoon Haiyan: *https://doi.org/101177/1077801218790701*. 2018;25(4):421-440. doi:10.1177/1077801218790701
27. Nguyen H, Rydström H. Climate Disaster, Gender, and Violence: Men’s Infliction of Harm upon Women in the Philippines and Vietnam. *Womens Stud Int Forum*. 2018;71:56-62. doi:10.1016/J.WSIF.2018.09.001
28. Tanyag M. Resilience, Female Altruism, and Bodily Autonomy: Disaster-Induced Displacement in Post-Haiyan Philippines. *https://doi.org/101086/695318*. 2018;43(3):563-585. doi:10.1086/695318
29. Madhuri. The impact of flooding in Bihar, India onwomen: a qualitative study. *Asian women*. 2016;32(1).
30. SF R, S M. Female adolescents and their sexuality: notions of honour, shame, purity and pollution during the floods. *Disasters*. 2000;24(1):54-70. doi:10.1111/1467-7717.00131
31. Singh D. Gender relations, urban flooding, and the lived experiences of women in informal urban spaces. *https://doi.org/101080/1225927620201817263*. 2020;26(3):326-346. doi:10.1080/12259276.2020.1817263
32. Fothergill A. An exploratory study of woman battering in the Grand Forks flood disaster: implications for community responses and policies. *Int J Mass Emerg Disasters*. Published online 1999.
33. Esho T, Komba E, Richard F, Shell-Duncan B. Intersections between climate change and female genital mutilation among the Maasai of Kajiado County, Kenya. Published online 2021. doi:10.7189/jogh.11.04033
34. Hossen MA, Benson D, Hossain SZ, Sultana Z, Rahman MM. Gendered Perspectives on Climate Change Adaptation: A Quest for Social Sustainability in Badlagaree Village, Bangladesh. *Water 2021, Vol 13, Page 1922*. 2021;13(14):1922. doi:10.3390/W13141922
35. Parkinson D, Zara C. The hidden disaster: domestic violence in the aftermath of natural disaster. *Aust J Emerg Manag*. Published online 2013.
36. D P. Investigating the Increase in Domestic Violence Post Disaster: An Australian Case Study. *J Interpers Violence*. 2019;34(11):2333-2362. doi:10.1177/0886260517696876
37. Carnaby E, Saladoka J, Vulavou I, et al. Down By The River Addressing the Rights, Needs and Strengths of Fijian Sexual and Gender Minorities in Disaster Risk Reduction and Humanitarian Response . *Oxfam Res Reports*. Published online 2018. Accessed February 14, 2022. www.oxfam.org.au
38. Houghton R, Wilson T, Smith W, Johnston D. Houghton: Domestic Violence Reporting “If There Was a Dire Emergency, We Never Would Have Been Able to Get in There”: Domestic Violence Reporting and Disasters. *Int J Mass Emerg Disasters*. 2010;28(2):270-293.
39. Asadullah MN, Islam KMM, Wahhaj Z. Child marriage, climate vulnerability and natural disasters in coastal Bangladesh. *J Biosoc Sci*. Published online 2020:1-20. doi:10.1017/S0021932020000644
40. Azad AK, Hossain KM, Nasreen M. Flood-induced vulnerabilities and problems encountered by women in northern Bangladesh. *Int J Disaster Risk Sci 2013 44*. 2014;4(4):190-199.

doi:10.1007/S13753-013-0020-Z
41. Memon FS. Climate Change and Violence Against Women: Study of A Flood-Affected Population in The Rural Area of Sindh, Pakistan. *Pakistan J Women's Stud Alam-e-Niswan*. 2020;27(1):65-85. doi:10.46521/PJWS.027.01.0039
42. Ahmed KJ, Haq SMA, Bartiaux F. The nexus between extreme weather events, sexual violence, and early marriage: a study of vulnerable populations in Bangladesh. *Popul Environ 2019 403*. 2019;40(3):303-324. doi:10.1007/S11111-019-0312-3
43. International Federation of Red Cross and Red Crescent Societies. *Gender-Based Violence Prevention and Response during Natural Disasters Research Results and Recommendations Philippines Indonesia Lao PDR*.
44. Bradley T, Martin Z, Upreti BR, Subedu B, Shrestha S. Gender and Disaster: The Impact of Natural Disasters on Violence Against Women in Nepal: *https://doi.org/101177/00219096211062474*. Published online December 28, 2021. doi:10.1177/00219096211062474
45. Campbell DW, Campbell JC, Yarandi HN, et al. Violence and abuse of internally displaced women survivors of the 2010 Haiti earthquake. *Int J Public Health*. 2016;61(8):981-992. doi:10.1007/S00038-016-0895-8/TABLES/7
46. Cerna-Turoff I, Kane JC, Devries K, Mercy J, Massetti G, Baiocchi M. Did internal displacement from the 2010 earthquake in Haiti lead to long-term violence against children? A matched pairs study design. *Child Abuse Negl*. 2020;102:104393. doi:10.1016/J.CHIABU.2020.104393
47. Chan KL, Zhang Y. Female Victimization and Intimate Partner Violence After the May 12, 2008, Sichuan Earthquake. *Violence Vict*. 2011;26(3):364-376. doi:10.1891/0886-6708.26.3.364
48. Irshad H, Mumtaz Z, Levay A. Long-term gendered consequences of permanent disabilities caused by the 2005 Pakistan earthquake. *Disasters*. 2012;36(3):452-464. doi:10.1111/J.1467-7717.2011.01265.X
49. Fisher S. Violence against women and natural disasters: Findings from post-tsunami sri lanka. *Violence Against Women*. 2010;16(8):902-918. doi:10.1177/1077801210377649
50. Kolbe AR, Hutson RA, Shannon H, et al. Mortality, crime and access to basic needs before and after the Haiti earthquake: a random survey of Port-au-Prince households. *https://doi.org/101080/136236992010535279*. 2010;26(4):281-297. doi:10.1080/13623699.2010.535279
51. Lai BS, Osborne MC, De Veauuse-Brown N, Swedo EA, Self-Brown S, Massetti GM. Violence victimization and negative health correlates of youth in post-earthquake Haiti: Findings from the cross-sectional violence against children survey. *J Affect Disord*. 2020;270:59-64. doi:10.1016/J.JAD.2020.03.050
52. Logie CH, Daniel CA, Ahmed U, Lash R. 'Life under the tent is not safe, especially for young women': understanding intersectional violence among internally displaced youth in Leogane, Haiti. *Glob Health Action*. 2017;10(sup2). doi:10.1080/16549716.2017.1270816
53. Rao S. A natural disaster and intimate partner violence: Evidence over time. *Soc Sci Med*. 2020;247:112804. doi:10.1016/J.SOCSCIMED.2020.112804
54. Rahill GJ, Joshi M, Lescano C, Holbert D. Symptoms of PTSD in a sample of female victims of sexual violence in post-earthquake Haiti. *J Affect Disord*. 2015;173:232-238. doi:10.1016/J.JAD.2014.10.067
55. Rees S, Pittaway E, Bartolomei L. Waves of violence: women in post-tsunami Sri Lanka. *Polit Sci*. Published online 2005.
56. Sakurai K, Nishigori H, Nishigori T, et al. Incidence of Domestic Violence Against Pregnant Females After the Great East Japan Earthquake in Miyagi Prefecture: The Japan Environment and Children's Study. *Disaster Med Public Health Prep*. 2017;11(2):216-226. doi:10.1017/DMP.2016.109
57. Sloand E, Killion C, Yarandi H, et al. Experiences of violence and abuse among internally displaced adolescent girls following a natural disaster. *J Adv Nurs*. 2017;73(12):3200-3208. doi:10.1111/JAN.13316
58. Sohrabizadeh S. A Qualitative Study of Violence Against Women after the Recent Disasters of

Iran. *Prehosp Disaster Med*. 2016;31(4):407-412. doi:10.1017/S1049023X16000431
59. Sohrabizadeh S, Jahangiri K, Jazani RK, Babaie J, Moradian MJ, Rastegarfar B. Women’s Challenges and Capabilities in Disasters: A Case Report of the Twin Earthquakes of Eastern Azerbaijan, Iran. *PLoS Curr*. 2017;9. doi:10.1371/CURRENTS.DIS.2CFF3D6E9E0C3A597F873BF29E712370
60. Standing K, Parker S, Bista S. Grassroots responses to violence against women and girls in post-earthquake Nepal: lessons from the field. *https://doi.org/101080/1355207420161194562*. 2016;24(2):187-204. doi:10.1080/13552074.2016.1194562
61. Subedi S, Davison C, Bartels S. Analysis of the relationship between earthquake-related losses and the frequency of child-directed emotional, physical, and severe physical abuse in Haiti. *Child Abuse Negl*. 2020;106:104509. doi:10.1016/J.CHIABU.2020.104509
62. Tanoue K, Nishigori H, Watanabe Z, et al. Interannual Changes in the Prevalence of Intimate Partner Violence Against Pregnant Women in Miyagi Prefecture After the Great East Japan Earthquake: The Japan Environment and Children’s Study. *J Interpers Violence*. 2021;36(21-22):10013-10028. doi:10.1177/0886260519881517
63. Tearne JE, Guragain B, Ghimire L, Leaning J, Newnham EA. The health and security of women and girls following disaster: A qualitative investigation in post-earthquake Nepal. *Int J Disaster Risk Reduct*. 2021;66:102622. doi:10.1016/J.IJDRR.2021.102622
64. Yoshihama M, Yunomae T, Tsuge A, Ikeda K, Masai R. Violence Against Women and Children Following the 2011 Great East Japan Disaster: Making the Invisible Visible Through Research. *Violence Against Women*. 2019;25(7):862-881. doi:10.1177/1077801218802642
65. Weitzman A, Behrman JA. Disaster, Disruption to Family Life, and Intimate Partner Violence: The Case of the 2010 Earthquake in Haiti. *Sociol Sci*. 2016;3:167-189. doi:10.15195/V3.A9
66. IGLHRC/SEROVie. *The Impact of the Earthquake, and Relief and Recovery Programs on Haitian LGBT People*.; 2011. Accessed February 14, 2022. https://outrightinternational.org/sites/default/files/504-1.pdf
67. Human Rights Watch. Failure to Protect Women’s and Girls’ Right to Health and Security in Post-Earthquake Haiti | HRW. Accessed February 14, 2022. https://www.hrw.org/report/2011/08/19/nobody-remembers-us/failure-protect-womens-and-girls-right-health-and-security
68. In Post-Earthquake Haiti, Activists Fight Violence Based on Gender and Sexuality. Accessed February 14, 2022. https://observers.france24.com/en/20101114-post-earthquake-haiti-activists-fight-violence-based-gender-sexuality
69. CASP CHECKLISTS - CASP - Critical Appraisal Skills Programme. Accessed August 12, 2021. https://casp-uk.net/casp-tools-checklists/
